# easyCLIP analysis of RNA-protein interactions incorporating absolute quantification

**DOI:** 10.1038/s41467-021-21623-4

**Published:** 2021-03-10

**Authors:** Douglas F. Porter, Weili Miao, Xue Yang, Grant A. Goda, Andrew L. Ji, Laura K. H. Donohue, Maria M. Aleman, Daniel Dominguez, Paul A. Khavari

**Affiliations:** 1 Program in Epithelial Biology, Stanford University, Stanford, CA USA; 2 Stanford Program in Cancer Biology, Stanford University, Stanford, CA USA; 3 Department of Pharmacology, University of North Carolina at Chapel Hill, Chapel Hill, NC USA; 4 Department of Chemistry, University of North Carolina at Chapel Hill, Chapel Hill, NC USA; 5 Department of Genetics, Stanford University, Stanford, CA USA; 6 Lineberger Comprehensive Cancer Center, University of North Carolina at Chapel Hill, Chapel Hill, NC USA; 7 Veterans Affairs, Palo Alto Healthcare System, Palo Alto, CA USA

**Keywords:** RNA sequencing, Cancer

## Abstract

Quantitative criteria to identify proteins as RNA-binding proteins (RBPs) are presently lacking, as are criteria to define RBP target RNAs. Here, we develop an ultraviolet (UV) cross-linking immunoprecipitation (CLIP)-sequencing method, easyCLIP. easyCLIP provides absolute cross-link rates, as well as increased simplicity, efficiency, and capacity to visualize RNA libraries during sequencing library preparation. Measurement of >200 independent cross-link experiments across >35 proteins identifies an RNA cross-link rate threshold that distinguishes RBPs from non-RBPs and defines target RNAs as those with a complex frequency unlikely for a random protein. We apply easyCLIP to the 33 most recurrent cancer mutations across 28 RBPs, finding increased RNA binding per RBP molecule for KHDRBS2 R168C, A1CF E34K and PCBP1 L100P/Q cancer mutations. Quantitating RBP-RNA interactions can thus nominate proteins as RBPs and define the impact of specific disease-associated RBP mutations on RNA association.

## Abstract (web summary)

It is important to develop quantitative criteria to identify RNA-binding proteins and target RNAs. Here, the authors present a method to quantify cross-linking and apply it to recurrent cancer mutations in RNA-binding proteins.

## Introduction

Approaches to quantify protein–RNA cross-links on a per-molecule basis are not widely available, leading to confusion both as to what constitutes an RNA-binding protein (RBP) and to the quantitative impact of disease-associated RBP mutations. For example, only roughly half of the proteins either in the RBP census^1^ or with an RNA-binding Gene Ontology (GO) term are considered RBPs by both sources. Landmark proteomic efforts from multiple groups have identified many potential novel RBPs^2–7^; some, such as sequestosome-1^8^, were subsequently verified and studied by cross-linking immunoprecipitation (CLIP), while the vast majority of which have not yet been evaluated by non-proteomic approaches^9^. Many important proteins studied in a different context have been categorized as also binding RNA, yet few or no experiments have been published on their functions in RNA binding. For example, proteins important to cancer, such as BRCA1, SMAD3-4, SPEN, CHD2, and JUN, have been categorized as RBPs, yet are not generally studied as such, raising the question as to whether they actually act in that role. Addressing this question for such proteins, and for additional potentially novel RBPs, has been hindered by the lack of a test that quantitates RNA interaction events per protein molecule to provide a global cutoff level of RNA binding to nominate a protein as an RBP.

Currently, there is no general method to estimate absolute RNA–protein interaction frequencies and a quantitative test is needed to assess whether any nonrandom interaction with an RNA exists. The frequencies of RNA–protein complexes, per-cell and per-interaction partner, would enable the fundamental characterization of RNA–protein interaction networks. Determining the targets of an RBP by conventional approaches, such as enrichment over negative control immunopurification or by clustering of cross-links^10^, are ultimately but indirectly determining if the absolute count of an RNA–protein complex in the cell is abnormally high. Defining RNA–protein interaction events per cell and per protein in absolute quantities, in contrast, may provide a framework for describing a global and widely reproducible view of RNA–protein interactions.

A number of RBPs are mutated in human cancers; however, the impact of such mutants on their association with RNA has not been quantitated. Most tumors have aberrant splicing without apparent mutational cause^11,12^, indicating that there must be unknown mutations within or affecting RNA metabolism pathways that are collectively common. There are widespread RBP expression changes in tumors, and the alternative splicing of tumor cells is predicted to affect cancer hallmarks, with some tumor types reverting to a more undifferentiated splicing pattern^13^. Many cancer-associated genes are potentially RBPs and some RBPs contain recurrent missense mutations. More generally, recurrent mutations that are not exceptionally frequent are of unknown significance^14^.

Here, we report a refinement of current CLIP protocols, termed easyCLIP. easyCLIP quantifies RNA cross-links per protein and provides visual confirmation of each step. easyCLIP enables the calculation of the distribution of cross-linking for the average protein and we propose a quantitative threshold for whether a protein is an RBP. Establishing a distribution for non-RBPs enables the definition of specific target RNAs for any RBP as those interactions with a frequency per protein are unlikely to occur with a randomly selected protein. easyCLIP is applied to the top 33 most frequent missense mutations across 28 RBPs, identifying quantitative changes in RNA binding in specific RBPs that are recurrently mutated in cancer. easyCLIP represents a method with built-in verifications that enables quantification of the number of RNA cross-links per protein in wild-type (WT) and disease-associated mutant RBPs.

## Results

### RNA-binding proteins associated with cancer

**Fig. 1:**
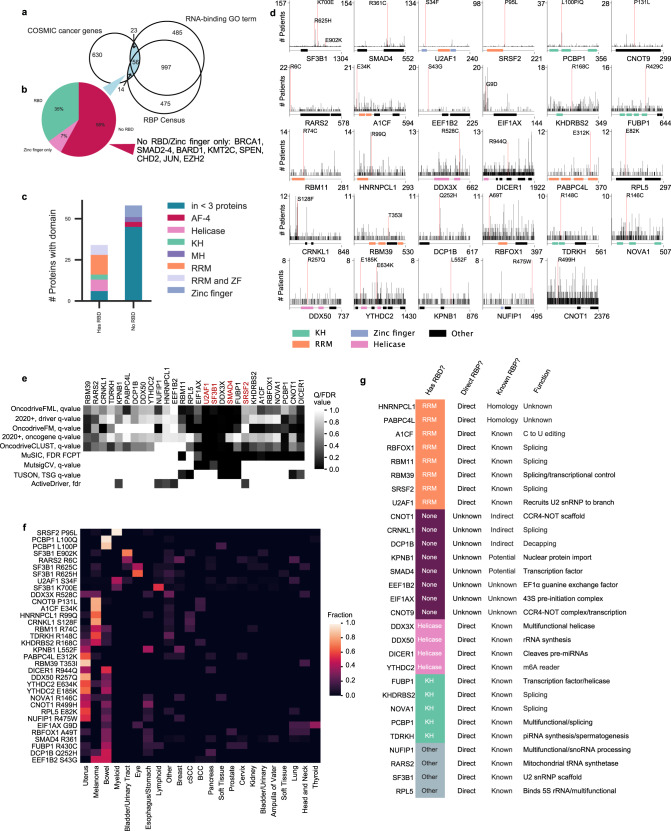
Ambiguity in the classification of RBPs and recurrent missense mutations in RBPs. RBD, RNA-binding domain. **a** Overlap between proteins with a GO term of RNA binding, the RBP census^1^, and genes implicated in cancer by COSMIC^15^. **b** RBPs implicated in cancer with or without a clear RNA-binding domain. **c** Domains in RBPs implicated in cancer. **d** The most recurrent missense mutations in RBPs across all cancer types, from TCGA data. **e** Significance value for oncogenic or tumor suppressor gene potential for RBPs with the most frequent missense mutations in cancer. **f** Distribution of the given missense mutations by RBP across cancer type. **g** Features of RBPs with the most frequent missense mutations in cancer.

Two lists of RNA-binding proteins (GO terms and census) were compared with the COSMIC (Catalog of Somatic Mutations in Cancer) list of cancer-associated genes^15^ to identify 93 RBPs associated with cancer (Fig. 1a), of which 51 had no clear structured RNA-binding domain (RBD) (Fig. 1b). Proteins without an RBD did not have a common set of domains (Fig. 1c). Notable inclusions in this list that are not well-established direct RBPs include BRCA1, BARD1, SMAD2-4, SOX2, KMT2C, SPEN, CHD2, JUN, and EZH2. The Cancer Genome Atlas (TCGA) data^16^ was used to rank all recurrent missense mutations in RBPs by overall frequency (Fig. 1d), which recovered the top three most studied mutations in well-established RBPs in cancer as the top three most frequent (in SF3B1, U2AF1, and SRSF2), aside from the putative RBP SMAD4, supporting the usage of raw frequency as a basis for oncogenic potential. Statistical tests for cancer driver and tumor suppressor activities for the top 29 proteins gave a range of significant values^14^ (Fig. 1e). The tissue specificity of these missense mutations had an expected enrichment in tumor types with a higher single-nucleotide polymorphism rate, but showed variation (Fig. 1f). Of those proteins with recurrent missense mutations and RNA-binding GO terms, many are long-established direct binders, while others are included on the basis of interactome capture datasets^2,3,6^ (Fig. 1g). Further analysis was conducted on RBPs identified by phase separation methods^4,5,7^ and additional datasets (Supplementary Fig. 1). To understand the role of RBPs in cancer, a method was needed that (1) distinguished RBPs from non-RBPs, (2) reliably produced CLIP-seq data so that it could be applied to a large protein set, and (3) provided a general definition of nonrandom interactions.

### Library preparation by easyCLIP

**Fig. 2:**
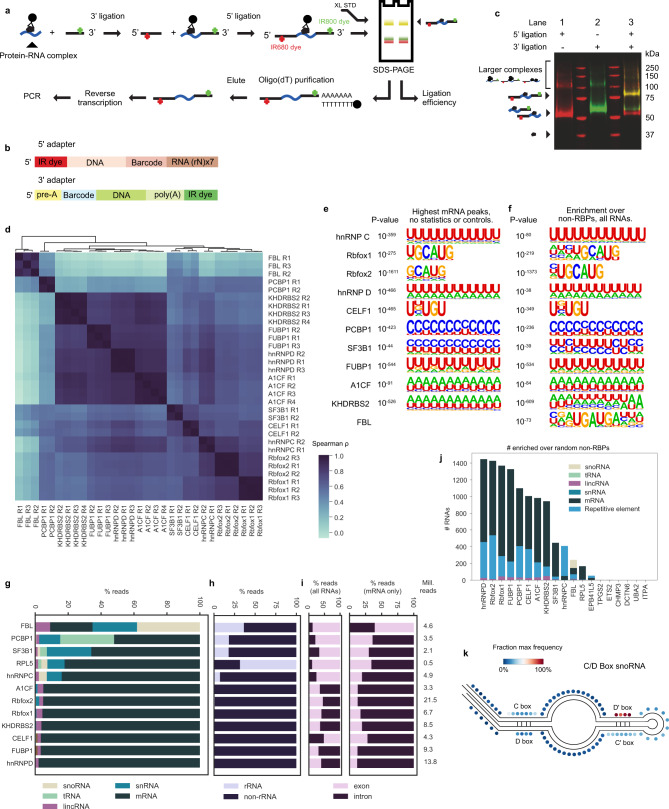
The easyCLIP protocol. **a** easyCLIP schematic. **b** Diagrams of the 5′ and 3′ adapters with IR680 dye (LI-COR) and IR800-CW dye, respectively. **c** A nitrocellulose membrane representing the gel in panel (**a**), in which CLIP libraries produced for the protein hnRNP C are visualized by the dyes on the adapters, the 5′ in red and the 3′ in green. The size of uncross-linked hnRNP C noted at left. “Larger complexes” contain ligated adapter and hnRNP C protein; they likely reflect the presence of additional proteins, but their exact nature is unknown. The experiment was performed three times with similar results. **d** Spearman’ *ρ* values for reads per gene from replicates of 11 RBPs, excluding RPL5 as it has only one definitive target RNA (5S rRNA). Endogenous hnRNP C, RBFOX2, and FBL were immunopurified, HA-PCBP1 was stably integrated, and the others were transiently expressed as HA-tagged forms. PCBP1 was expressed in HCT116 cells and the others in 293Ts. **e** The most significant motif identified de novo by HOMER for the top mRNA peaks. Motif enrichment significance computed by HOMER (hypergeometric test with *p* value corrected for multiple testing). **f** The most significant motif identified de novo by HOMER for all peaks significantly enriched over randomly chosen non-RBPs. **g** Fraction of easyCLIP reads of the indicated RBP mapping to RNAs of the given ENSEMBL biotype. **h** Fraction of reads mapping to rRNA. **i** Fraction of reads across all RNA types (left) or across mRNA (right) that map to introns or exons. **j** Number of significantly enriched (FDR < 10^−4^) categories of RNAs, relative to randomly selected non-RBP controls. “Mill. reads” denotes the number of uniquely mapping read pairs. **k** Fraction of FBL cross-link locations normalized to their average length across all C/D-box snoRNAs; one dot represents one nucleotide in the normalized snoRNA.

To generate a simpler and faster way of producing CLIP-seq datasets, a method was developed using on-bead ligations^17–19^ of 3′ adapters (termed L3) and 5′ adapters (termed L5), each with a different fluorescent dye (Fig. 2a, b). After running a sodium dodecyl sulfate-polyacrylamide gel electrophoresis (SDS-PAGE) gel and transferring to a membrane, single- and dual-ligated RNA were clearly visible (Fig. 2c and Supplementary Fig. 2). RNA was extracted from the membrane using proteinase K, purified using oligonucleotide(dT) beads to capture the poly(A) sequence on the L3 adapter, eluted, reverse transcribed, and input directly into PCR. Major differences from HITS-CLIP (high-throughput sequencing of RNA isolated by CLIP) include the usage of a chimeric DNA–RNA hybrid for efficient ligation, the purification of complexes from a membrane by oligo(dT), and the direct visualization of ligation efficiencies and finished libraries by infrared dyes (Supplementary Data 1 and please see Table 1 for how each step is verified). Since the only steps after the gel extraction are an oligonucleotide(dT) purification and reverse transcription (RT) before PCR, there are decreased opportunities for experimental failure after the diagnostic step of gel imaging. It is a caveat to all cross-linking-based studies that cross-linking is proportional to binding frequency through a poorly understood factor of complex cross-linking efficiency.

**Table 1:** easyCLIP troubleshooting.

Step	Verification
RNAse digestion	Verified by fluorescence on nitrocellulose (NC): under-digested sample runs at the top of the gel and over-digested sample has no smear upward in the minimal region.
5′ and 3′ ligations	Assessed by protein shifts and total fluorescence on NC. With more difficulty, the RNA and adapter shift methods are usable for exact determinations if the protein shift pattern is too complex. For RNA and adapter shifts, it is best to have library sizes >10 fmol.
Nitrocellulose to reverse transcription (RT)	The extraction from NC and oligonucleotide(dT) purification can be assayed by dot blotting the oligonucleotide(dT) eluate on nylon. Purified RNA must contain the L3 adapter, so fluorescence from the L5 adapter corresponds to the completed library.
Reverse transcription	Verified by comparison of qPCR amplification with input to RT. PCR reactions amplify more quickly the more complex the input sample. The L5 adapter fluorescence input to RT corresponds to the completed library. If L5 fluorescence input is ~1000 s, the input is in the femtomolar range, which should be visibly in the exponential PCR phase before cycle 14. It should never be necessary to amplify >~16 cycles. Completed libraries before RT can also be visualized by the RNA shift method.
Amount of input RNA	Determination of the 5′ ligation efficiency by any method and a quantified standard to translate fluorescence values into RNA molecule numbers allow for a determination of the total amount of input RNA.

easyCLIP was benchmarked against eCLIP (enhanced CLIP) in the manner in which eCLIP was benchmarked against iCLIP (individual nucleotide resolution CLIP), using Rbfox2. easyCLIP was more efficient (Supplementary Fig. 3A), and Rbfox2 libraries were reproducible (Supplementary Figs. 3–6), including deletions from cross-linking (Supplementary Fig. 4), and correlated with Rbfox2 eCLIP (Supplementary Figs. 4–6), including matching the pattern seen with eCLIP at NDEL1 (Supplementary Fig. 4), indicating that easyCLIP captures similar information. easyCLIP was then used to generate data for 11 additional known RBPs, chosen as representatives (FBL, which associates with C/D-box small nucleolar RNA (snoRNA), and other noncoding RNA (ncRNA), hnRNP C), at random (CELF1, hnRNP D), or for their relevance to cancer (the others). Endogenous hnRNP C, RBFOX2, and FBL were immunopurified, PCBP1 was stably integrated outside its genomic locus, and the others were transiently transfected in a manner to generate low expression (“Methods”), usually below or equal to endogenous protein (Supplementary Fig. 7).

easyCLIP libraries produced high-quality data in each case (Fig. 2d–k and Supplementary Data 2–5). Directly entering sequences under the tallest peaks for all messenger RNA (mRNA)-binding RBPs into a de novo motif discovery program^20^ resulted in the top motifs being the expected motifs in all cases (Fig. 2e), indicating high easyCLIP signal-to-noise ratios. Results agreed with in vitro motif selection in all cases, and 9/10 had a top-3-enriched motif in >50% of peaks (Supplementary Fig. 8). Using the MACS2 peak calling algorithm on easyCLIP and ENCODE (Encyclopedia of DNA Elements) project eCLIP peaks generated favorable comparisons, with good motif coverage, peak numbers (Supplementary Fig. 9), and replicate consistency (Supplementary Fig. 10). Using enrichment over controls also recovered all motifs (Fig. 2f and Supplementary Fig. 8). Cross-linked RNA types matched expectations (Fig. 2g–h), except the transfer RNA (tRNA) binding by PCBP1, addressed below. Under false discovery rate (FDR) <10^−4^ vs random non-RBPs (discussed below), target RNA numbers (Fig. 2j) and the total number of unique mapped reads were typical for CLIP (Supplementary Fig. 3D, E). Analysis of deletions indicated that a similar level of cross-link position replicates reproducibility and cross-link-induced base deletions as other CLIP methods^21^ (Supplementary Fig. 10). Using deletions allows FBL cross-linking positions within snoRNAs to be visualized in detail (Fig. 2k), and matched previous reports^22^, suggesting that easyCLIP may offer an advantage over iCLIP/eCLIP-like methods for short RNAs, where reads with reverse transcriptase stop near the 3′ end are unmappable. Together, these results indicate easyCLIP effectively captures RNA-binding information.

### Estimating absolute RNA quantities

**Fig. 3:**
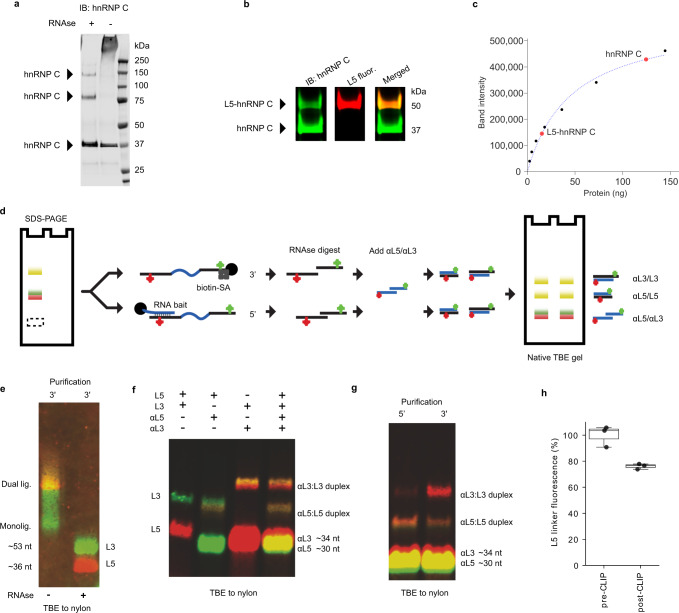
A method to quantify the absolute number of ligated RNA–protein complexes. **a** Fraction of hnRNP C UV cross-linked to RNA, including hnRNP C that cannot be collapsed to a monomeric band by RNAse. The experiment was performed twice with similar results. **b** Ligation of the L5 adapter creates a novel band of monomeric hnRNP C-RNA-L5. The experiment was performed three times with similar results. **c** Absolute quantification of the monomeric hnRNP C and L5-hnRNP C band using a standard curve of GST-hnRNP C quantifying the number of L5 molecules in the complex and thereby fluorescence per molecule. Black dots comprise the standard curve and red dots endogenous hnRNP C. **d** Schematic of the method to estimate the amount of fluorescence per L5 molecule lost in the CLIP procedure. **e** RNA purified as in **d** may be RNAse treated to collapse signal into adapter bands. The sizes of oligos are approximated with the dye molecule counting as 6 nt. The experiment was performed once. **f** The method in **d** applied to known amounts of free L5 and L3 adapter; note that adapters are completely shifted. The experiment was performed three times with similar results. **g** Application of the method in **d** to visualize fluorescence of hnRNP C-cross-linked RNA after a CLIP procedure. Visualization was performed once, not including the quantified samples below. **h** Fluorescence loss of L5 from the CLIP procedure in replicate experiments (*n* = 3). The box limits denote the 25–75% quartile range, the center line is the median, and whiskers show extreme values.

easyCLIP was tested for its ability to quantitate the total amount of RNA cross-linked to a protein. Prior work has ligated 3′ adapter molecules labelled with infrared dyes to count RNAs^23^, but this method does not account for unligated RNA, and is only accurate if there are no changes in dye fluorescence during the procedure or from imaging conditions. When cells were ultraviolet (UV) cross-linked, hnRNP C immunopurified, and RNA digested, a series of bands were visible (Fig. 3a), spaced at roughly the size of an hnRNP C dimer. When an ~15 kDa fluorescent adapter was ligated to highly digested hnRNP C-cross-linked RNA, a new band ~15 kDa above monomeric hnRNP C, containing adapter and hnRNP C, appeared (Fig. 3b). The amount of protein in this band was determined by quantitative western blotting (Fig. 3c and Supplementary Fig. 11A) after evaluating the antibody and standards (Supplementary Fig. 11B–G). Because the cross-linked band contains an equal number of protein and RNA molecules (Fig. 3b), quantification of the protein in the cross-linked band relates adapter fluorescence values in this band into an absolute molecule number. Quantification of fluorescence per molecule using a single preparation of cross-linked, quantified hnRNP C as an aliquoted standard can be used to translate fluorescence values to RNA quantities when fluorescence loss is low, and the ligation efficiency is approximated. This approach enabled absolute RNA quantities to be calculated for RNA cross-linked to protein.

### Fluorescence loss

To examine loss in adapter fluorescence from CLIP over the course of the method, antisense oligonucleotides to L5 and L3 were labelled with reciprocal dyes, hereafter termed αL5 and αL3, and used to shift their cognate adapter. Such antisense oligonucleotides shift the adapter molecules up in a native gel and produce bands with a 1:1 ratio of antisense and sense oligonucleotide (Fig. 3d). L5 and L3 were purified from proteinase K extract and RNAse digested to free adapters (Fig. 3e). 100% of L5 and L3 adapters were shifted (Fig. 3f) and the method was applied to RNAse-digested CLIP oligonucleotides (Fig. 3g). By comparing the ratio of αL5 to L5 for fresh L5 vs L5 extracted from the nitrocellulose membrane in CLIP, the L5 fluorescence loss from CLIP could be determined to consistently be only ~20% (Fig. 3h).

### Ligation efficiency

**Fig. 4:**
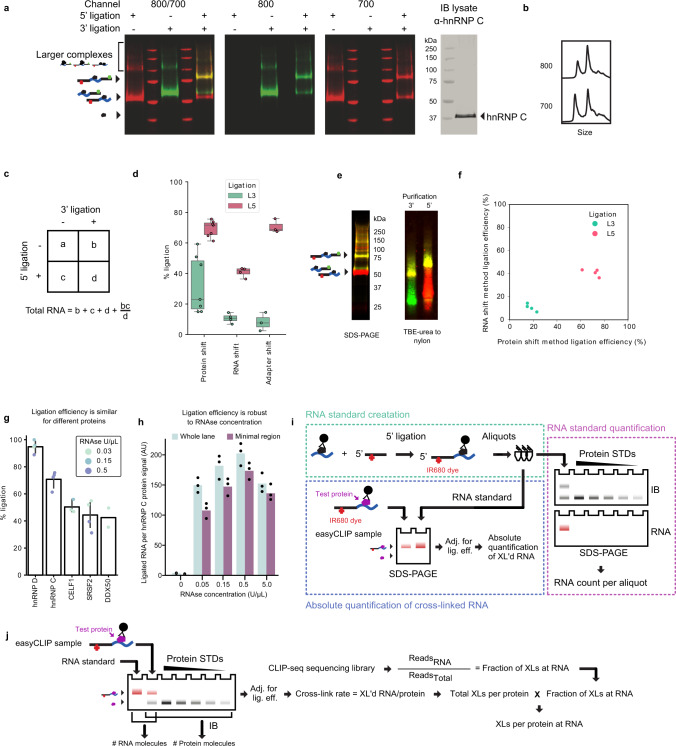
Estimating ligation efficiencies. **a** Products of single and dual ligations of the RBP hnRNP C; fluorescent markers ligated to RNA (left three panels) or a western blot of cell lysate (right panel). “Larger complexes” contain ligated adapter and hnRNP C protein; they likely reflect the presence of additional proteins, but their exact nature is unknown. The experiment was performed three times with similar results. IB: immunoblot. **b** Chromatographs generated from the fluorescence gel image in panel (**a**), far-right lane. **c** Calculations to determine total bound RNA from the three observed values and the assumption of statistical independence in ligation efficiencies to determine the fourth value. **d** Ligation efficiencies estimated by the protein shift method (*n* = 7), by RNA shift (*n*= 4), or by shifting free adapters (*n*= 3). Center line, median; bounds of box, 25–75% quartile range; whiskers, maxima, and minima. **e** Ligation efficiency by shifted RNA. The experiment was performed four times with similar results. **f** Correlation between ligation efficiencies determined by the protein shift method or the RNA shift method for four biological replicates. **g** Five representative RBPs have 5′ ligation efficiencies of 42–95%, as determined by protein shift. DDX50 binds rRNA, while the others are mRNA-binding proteins. hnRNP D binds AU-rich RNA, CELF binds UGU, hnRNP C binds poly(U), and SRSF2 binds a GA-rich motif. Fifty percent is a reasonable approximation for the 5′ ligation efficiency in general. Data are mean ± 95% CI. **h** The 5′ ligation efficiency is robust to RNAse concentration (*n* = 3 independent samples). RNAse concentrations of 0.05–5.0U/µL resulted in <2-fold changes in ligated RNA. This is likely due to the RNAse and the ligase having similar limits on RNA length for a 5′ fragment. Bars represent the mean. AU arbitrary units. **i** Diagram of the method of absolute RNA quantification. The amount of observed ligated RNA is multiplied by two based on the observed general ligation efficiency in order to determine the number of cross-linked RNA molecules. When a quantitative immunoblot is performed on the same gel for the uncross-linked protein, the RNA molecule count may be divided by the protein molecule count to derive the cross-link rate. **j** Method to combine sequencing data and cross-link rates to determine the cross-links to an RNA (or class of RNAs, region of RNAs, etc.) per protein molecule.

Three methods were used to estimate easyCLIP ligation efficiency. First, both fluorescent L5 and L3 adapters were ligated and visualized as single vs dual adaptor size shifts (Fig. 4a). By quantifying the amount of fluorescence signal in the single- and dual-ligated protein–RNA complexes, efficiency estimates can be obtained for both 5′ and 3′ (Fig. 4b, c). Based on the premise that the two ligations are independent events, the total amount of cross-linked RNA was also obtained, including unlabelled RNA (Fig. 4c), and L5 ligation efficiencies estimated (Fig. 4d). Cross-linking molecule number were estimated by replicate (Supplementary Data 6). For a second method, RNA was extracted from nitrocellulose using proteinase, purified using either L5 or L3, run on a gel, and transferred to a nylon membrane. Higher molecular weight bands on nitrocellulose, due to variation in cross-linked protein, were collapsed into two simple fluorescent smears, corresponding to mono-ligated and dual-ligated RNA (Fig. 4e). The logic of Fig. 4a–c was applied to the protein-free RNA in Fig. 4e to estimate ligation efficiencies, which were lower but also consistent between replicates (Fig. 4d, f). Because the shifted bands in Fig. 3g have a 1:1 ratio of L:αL oligonucleotides, quantifying antisense oligonucleotides also quantified their respective adapters. The development of an antisense oligonucleotide-based method to quantify low femtomole amounts of adapter necessitated optimization (Supplementary Figs. 12 and 13). For example, diluent had effects on fluorescence (Supplementary Fig. 12) and there was a systematic test of the effects of salt, carrier, and polyethylene glycol (PEG) to retain fluorescence, prevent sample loss from adhesion, account for signal interference, elute complexes from beads, and preserve complexes on a gel (Supplementary Figs. 12C–H and 13A–E), and testing with known concentrations of L5 and L3 adapter (Supplementary Fig. 13F–I). Altogether, these results supported the use of aliquots of a quantified standard to convert fluorescence to molecule number. From these three methods, ligation rates are stable between experiments at roughly 50 ± 20%. This 50 ± 20% adapter ligation efficiency was true for 4/5 tested proteins, with the exception displaying a higher efficiency (Fig. 4g). Since the 50% estimate can only underestimate by a factor of two, the 50% estimate is likely reliable for most RBPs to within a factor of two. The ligation rate was also stable over two orders of magnitude of RNAse digestion (Fig. 4h and Supplementary Fig. 14), which may be explained by the RNA ligase and RNAse having similar length requirements for substrates. The method of obtaining cross-linked RNA molecule counts was depicted (Fig. 4i and Supplementary Fig. 15), as were determination of cross-link rates (the RNA molecules cross-linked per total protein molecules) and combination with sequencing data (Fig. 4j). Taken together, these data identify ligation efficiency estimates in easyCLIP.

### Cross-link rates for RBPs

**Fig. 5:**
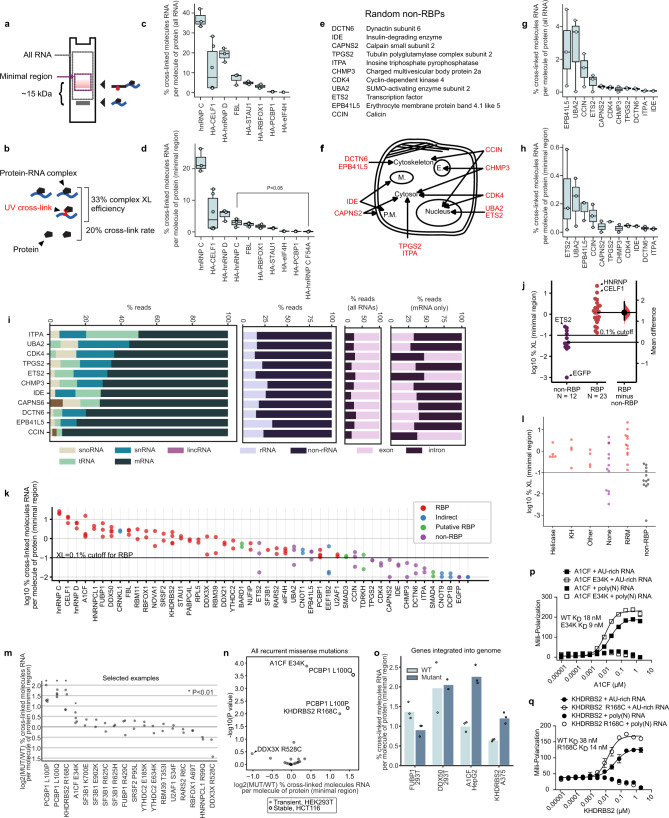
RNA cross-link rates for RBPs and non-RBPs are both diverse and distinct. Boxplots show quartiles, center line shows the median, and whiskers show the maxima and minima. **a** Schematic of the minimal region and total cross-linked RNA. **b** Definition of terms “cross-link rate” and “complex cross-link efficiency”. **c** Protein cross-link rates of RBPs (CELF1 *n* = 6, PCBP1 *n* = 4, RBFOX1 and STAU1 n = 2, others *n*= 3). **d** The percent cross-linking to RNA for the indicated proteins, including only RNA within the minimal region (replicate numbers as above). **e** Eleven randomly selected proteins that are not known to bind RNA in vivo or in vitro. **f** Subcellular locations of the randomly selected non-RBPs, as annotated on Uniprot^5^. **g** UV cross-link rates to RNA for randomly selected, HA-tagged non-RBPs, measuring all RNA (EPB41L5, UBA2, ETS2, and CCIN *n* = 3, others *n* = 2). **h** UV cross-link rates to RNA for randomly selected non-RBPs, including only RNA in the minimal region (EPB41L5, UBA2, ETS2, and CCIN *n*= 3, others *n*= 2). **i** The fraction of easyCLIP reads that map to RNAs of the given category. **j** Comparison of minimal region cross-link rates between RBPs (maroon) and non-RBPs (EGFP included, violet); vertical line indicates 95% confidence interval. **k** Minimal region cross-link rates for RBPs with missense mutations in cancer, along with other RBPs, putative RBPs, and non-RBPs. TDRKH is listed as a putative RBP here because the cross-link rate was determined in 293T cells, which do not express the only known target of TDRKH, piRNA. **l** Minimal region cross-link rate by RBD. Red dots indicate RBPs, purple indicates proteins that may or may not have a direct RNA interaction, and gray dots indicate non-RBPs. **m** Selected examples of changes in overall RNA binding for recurrent cancer missense mutations in RBPs. **n** Volcano plot of changes in overall RNA binding for recurrent cancer missense mutations in RBPs. All except PCBP1 are transiently expressed in HEK293T cells. **o** Cross-linking for selected proteins after stable integration into the genome in the indicated cell type (bars denote mean). **p**–**q** Fluorescence polarization assays of bacterially expressed WT and mutant A1CF and KHDRBS2 show ~2-fold increase in affinity for AU-rich RNA. Representative experiments are shown. The experiment was repeated three times with two different protein preparations for each protein pair, all showing a similar increase in affinity for the mutant RBPs. Data are mean ± s.d.

To further enhance quantitation, two measures of RNA cross-linked to protein were determined, (a) all RNA and (b) minimal region RNA (Fig. 5a). “All RNA” reflects the cross-linked RNA on the nitrocellulose membrane at the minimum size for a small protein–L5 complex (~30 kDa) and everything larger. The “minimal region” RNA measurement was taken from the region corresponding to the size for the dominant protein band cross-linked to small RNA fragments and ligated to L5, a region more likely to correspond to direct cross-linking events (Fig. 5a). We also distinguish between cross-link rates and complex cross-link efficiencies (Fig. 5b). For “all RNA”, cross-link rates were first determined for the RBPs hnRNP C (37%), FBL (7%, Supplementary Fig. 11H–I), hnRNP D (19%), Rbfox1 (40%), CELF1 (21%), STAU1 (4.9%), PCBP1 (0.5%), and eIF4H (0.3%) (Fig. 5c and Supplementary Fig. 16). Cross-links in the minimal region (Fig. 5d) were determined for RBPs hnRNP C (22%), FBL (2%), Rbfox1 (18%), CELF1 (11%), hnRNP D (5%), STAU1 (1.2%), PCBP1 (0.2%), and eIF4H (0.2%). Viral integration led to modestly higher expression levels and lower cross-link rates (Supplementary Fig. 17). STAU1 is known to be a very poor cross-linker^24^, so its rate may be taken as a representative. The accuracy of this method was tested by calculating the cross-link rate of hnRNP C by quantitative western blotting of immunopurified hnRNP C (Supplementary Fig. 18), which was within ~10%. It was next determined if easyCLIP could detect a loss in RNA-binding affinity caused by the F54A mutant of hnRNP C, a mutation in the RNA-binding surface of the RRM (RNA-recognition motif) that elevates the RRM’s in vitro *K*_D_^25^. The mutant was dramatically less cross-linked (Fig. 5d).

### Cross-linking rates distinguish RBPs and non-RBPs

Quantification of cross-link rates may identify a numerical threshold for distinguishing RBPs from non-RBPs and for determining when an RBP has lost or gained RNA-binding activity. To derive a distribution of cross-link rates for non-RBPs, non-RBP proteins were randomly chosen using a randomizing selection algorithm (Fig. 5e, f). Selected non-RBPs, hemagglutinin (HA)-tagged and transiently transfected in 293Ts in 10 cm plates (~2 × 10^7^ cells), had total RNA cross-link values of 0.03–4% (Fig. 5g and Supplementary Fig. 19), with rates correlated with protein size (Supplementary Fig. 20A). Reducing counts to minimal region RNA dropped all cross-link rates of all but four to <0.1% (Fig. 5h). Deletion rates indicated that purified RNA was cross-linked (Supplementary Fig. 10) and gel visualization of RNA cross-linked to protein indicated that RNA was cross-linked to the purified protein (Supplementary Fig. 19). Strangely, two of the high % cross-linkers, CCIN and EPB41L5, generated largely empty sequencing datasets, indicating their high cross-link rate is an artifact. Different putative non-RBPs produced distinct RNA interactions (Fig. 5i and Supplementary Fig. 20). Unlike RBPs, non-RBPs show little variation from ~25% intronic reads (Fig. 5i). These results indicate that cross-link rates derived from a minimal region are typically <0.1% for non-RBPs and >0.1% for RBPs (Fig. 5j), while determining if the sequencing library is empty may help eliminate artifacts. These results are in agreement with previous estimates of the fraction of RBP immunoblot signal co-purified with RNA after cross-linking being in the range <1–10% for at least several RBPs^5,26–28^. These metrics can be used to aid in defining what proteins are RBPs. For example, the hnRNP C^F54A^ mutant had a minimal region cross-link rate of 0.1%, consistent with losing most direct affinity for RNA but still joining an RNA-binding complex.

### Cancer-associated RBPs

The most frequent missense mutations in potential RBPs in cancer were identified using TCGA data^16^ (Fig. 1d). Cross-link rates were determined for 33 recurrent mutations across 28 known or potential RBPs, tagged and transiently expressed in 293T cells (Fig. 5j, k and Supplementary Figs. 7 and 21–22), plus additional proteins. All expected direct RBPs cross-linked above 0.1% (Fig. 5j, k). BRCA1, CRNKL1, and BCLAF1 would pass our cutoffs, but too small an amount of protein could be purified for confident assignment, and BRCA1 libraries were largely empty, indicative of an artifact. As a group, this larger dataset supports a 0.1% cutoff for RBPs (Fig. 5j) and the type of RBD did not have a strong effect on cross-link rate (Fig. 5l). Four recurrent mutations had effects on cross-linking: L100P and L100Q of PCBP1, A1CF^E34K^, and KHDRBS2^R168C^ (Fig. 5m, n). Interestingly, all four demonstrated increases in binding, consistent with these recurrent mutations potentially being gain of function. We are not aware of a previous example of a cancer-associated mutation increasing protein binding to RNA. To study this, we integrated several proteins into the genome (Supplementary Fig. 17); KHDRBS2^R168C^ in melanoma cells and A1CF^E34K^ integrated into HepG2 showed even larger increases (~2-fold) in cross-link rate vs WT controls (Fig. 5o). Integrated FUBP1^R429C^ and DDX3X^R528C^ showed decreased cross-link rates vs WT (Fig. 5o and Supplementary Fig. 17). Increased cross-linking for mutant KHDRBS2 and A1CF was confirmed in vitro, with mutant forms binding RNA ≥2-fold better than WT (Fig. 5p, q). PCBP1^L100P^ aggregated out of solution upon cleavage of the purification tag (Supplementary Fig. 16D). These data indicate that recurrent cancer mutations in specific RBPs may be associated with differences in RNA cross-link rates to their corresponding associated protein.

### Defining specific interactions of RBPs and non-RBPs

**Fig. 6:**
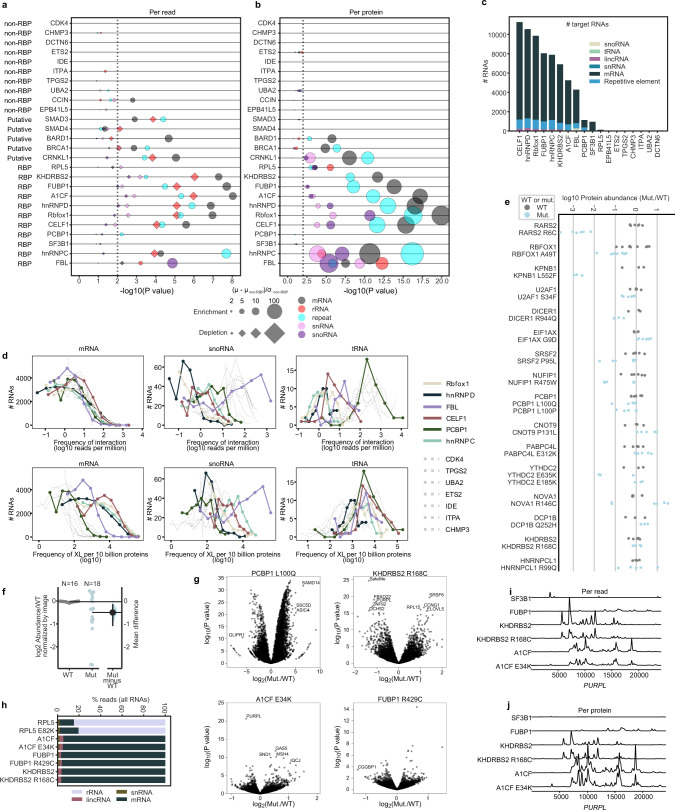
easyCLIP characterizes in vivo RNA–protein interaction landscapes. **a** The enrichment of selected RNA categories relative to randomly selected non-RBPs, on a per-read basis. All reads (per million) at the given RNA category were summed for each replicate and the average across replicates is *µ*. The average *µ* for randomly selected non-RBPs is *µ*_non-RBP_, and the standard deviation of *µ* within that group is *σ*_non-RBP_. The size of the markers denotes the enrichment or depletion of a given category as (*µ*−*µ*_non-RBP_)/*σ*_non-RBP_. *P* values are two-sided *t* tests vs the randomly selected non-RBPs (no multiple hypothesis adjustment). **b** The enrichment of selected RNA categories relative to randomly selected non-RBPs, on a per-protein basis. The calculation was the same as per read except that XLs per protein was used instead. **c** The number of significantly enriched (FDR < 10^−4^) RNAs of the given category for interactions per protein, relative to randomly selected non-RBP controls. **d** Histograms of cross-links per protein for RBPs and randomly selected non-RBPs; points indicate the left edge of each histogram bin; lines are for easier visualization. (Top) The *x*-axis is the rate of cross-linking per million cross-linked molecules (i.e., reads per million) to a given RNA converted to a log10 scale, and *y*-axis is the number of RNAs at that cross-link rate. (Bottom) The *x*-axis is the rate of cross-linking per 10 billion protein molecules to the given RNA (i.e., reads per million, per 10^4^ proteins), converted to a log10 scale, and the *y*-axis is the number of RNAs at that cross-link rate. **e** Protein abundances of WT and mutant RBPs. **f** Recurrent missense mutations and RBP protein levels (*n* = 16 WT, *n* = 18 mutant proteins). For the estimation of difference, the center is the mean and the vertical line indicates the 95% confidence interval. **g** Volcano plots of changes in easyCLIP reads per RNA induced by recurrent missense mutations in RBPs. *DCSH2* and *A1CF* were increased in E34K above the plotted *y* values. **h** Categories of RNAs cross-linked to missense mutant RBPs. **i** Cross-linking across the *PURPL* lncRNA is increased per read in KHDRBS2 and A1CF mutants. SF3B1 and FUBP1 are included for comparison. PCBP1 had no cross-linking to *PURPL*. Data are smoothed and plots have the same *y*-scale. **j** Cross-linking across the *PURPL* lncRNA on a per-protein basis. RNA-seq (Table 8) suggests *PURPL* lncRNA may be less abundant in cells with R168C KHDRBS2. Together, these results are consistent with a model where affinity for *PURPL* actually increases (resulting in similar cross-linking per protein), while *PURPL* RNA levels decrease, resulting in a loss in per-read cross-link frequency.

One of the goals of this study was to enable target RNAs to be defined for a protein of interest as those interactions with a frequency per protein or per cross-link unlikely to occur with a randomly selected protein. To address this issue, easyCLIP libraries for ten of the random non-RBPs were prepared. Using the resulting distribution of RNA interactions for random proteins, it is possible to directly estimate how unusual any RNA–protein interaction pair is. This method was first applied to frequencies per cross-link (per read) and RNA biotypes. RBPs tended to have a significant reduction in the fraction of reads mapping to ribosomal RNA (rRNA), and an increase in reads mapping to mRNA, relative to non-RBPs (Fig. 6a). Assuming that the interactions of non-RBPs are largely random, it was expected that RBPs contain similar random interactions, plus their evolved interactions. That is, the depletion or rRNA and small nuclear RNA (snRNA) in Fig. 6a is probably an artifact, not a representation of RBPs avoiding rRNA or snRNA. A more realistic view might be to use the cross-link rate to convert to cross-links per protein (Fig. 6b). This view appears more accurate in several regards. First, RBPs are not generally depleted for rRNA or snRNA interactions. Second, effect sizes are generally larger and significances higher—RBPs are more different from non-RBPs. Third, FBL has a more expected distribution, targeting snRNA and showing a greater enrichment for snoRNA and rRNA. Fourth, a number of other RBPs show more correct biological distributions (RPL5 binds rRNA, FUBP1/CELF1/RBFOX1 enrich for snRNA rather than being depleted).

Target RNAs were next identified as those bound per read at an unusually high rate. The success of this method is supported by the identification of the expected motif for all RBPs as the top motif (Fig. 2f), and target RNA types fit expectations (Fig. 2j). For example, FBL targeted snoRNA, hnRNPs targeted mRNA, and non-RBPs had few target RNAs. The sparse data for non-RBPs limits this method when applied to mRNA. Finally, target RNAs were identified as those bound per protein at an unusually high rate. Most mRNA-binding proteins appear to target most expressed mRNA at some level (Fig. 6c). The rate of cross-linking per protein was plotted for mRNAs (Fig. 6d, left), snoRNAs (middle), or tRNA (right), which suggested some fundamental results. First, the distribution of cross-linking across mRNAs, in reads per million, is similar between RBPs and non-RBPs (top left), but RBPs have more frequent mRNA partners per protein. snoRNA presents a different picture (middle). If looked at only by reads per million, it would seem that either randomly selected proteins target snoRNA or else RBPs somehow avoid it. Per protein, however, mRNA-binding RBPs and non-RBPs are equally likely to contact snoRNA—consistent with only FBL having specific interactions with snoRNA (bottom middle). This helps explain the tRNA binding by PCBP1 (Fig. 2g). Like snoRNAs, tRNAs make up a disproportionate share of the libraries of non-RBPs (top right), but per protein all RBPs and non-RBPs have the same distribution (bottom right). The distribution of tRNA binding by PCBP1 is actually just that of a non-RBP, consistent with a lack of evolved interaction with tRNA. Taken together, these data indicate that easyCLIP enables identification of target RNAs for RBPs of interest.

### Recurrent cancer mutations tend to destabilize RBPs

A puzzling result of RBP cancer mutants was that, of the RBPs for which relative expression levels were determined, mutants were less expressed (*P* < 0.05, two-sided *t* test, Fig. 6e, f). The apparent destabilization effect was particularly strong for RARS2^R6C^ (Fig. 6e), a mutation that causes mitochondrial encephalopathy^29^ and is found in cancer (Fig. 1f). The role that such potential RBP destabilization may play in cancer remains to be explored.

### Recurrent cancer mutations alter RNA–protein interactions

**Fig. 7:**
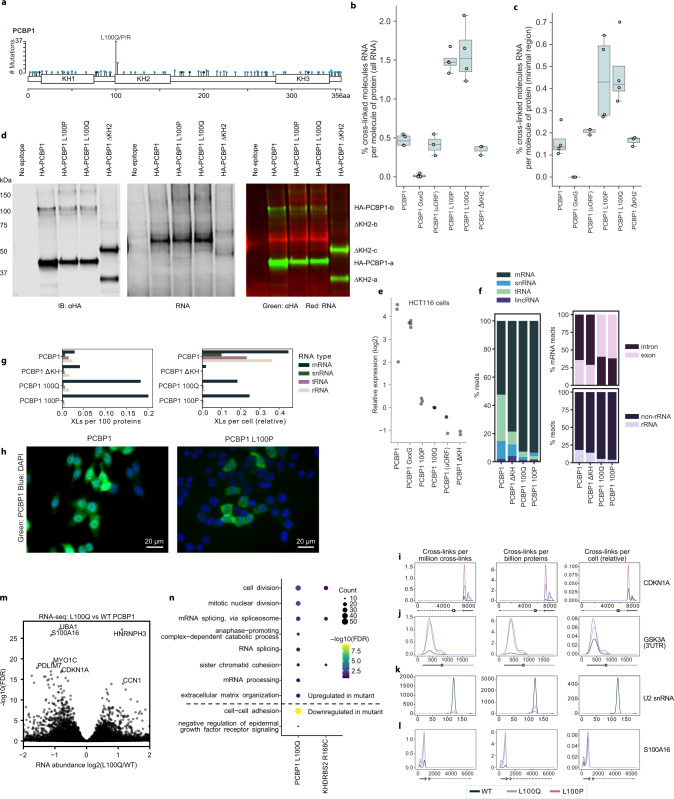
easyCLIP analysis of cancer-associated missense mutations in PCBP1. Boxplots show quartiles, center line shows the median, and whiskers show the maxima and minima; if a value is beyond 1.5 times the interquartile range, it is plotted without a whisker. **a** Locations of missense (blue) and nonsense (black) mutations in PCBP1. **b** Cross-link rates of wild-type and mutant HA-tagged PCBP1 (PCBP1 WT, GxxG, L100P, and L100Q *n* = 4, others *n* = 3). **c** Cross-link rates of wild-type and mutant PCBP1, including only RNA signal in the minimal region (PCBP1 WT, GxxG, L100P, and L100Q *n* = 4, others *n* = 3). PCBP1 (uORF) represents wild-type PCBP1 expressed from a vector with a uORF to lower expression levels. **d** Visualization of immunopurified HA-PCBP1 by immunoblot, and cross-linked RNA by L5 adapter fluorescence. Experiment was performed three times with similar results. **e** Expression levels of wild-type and mutant HA-PCBP1 in HCT116 lysate, as determined by immunoblot and normalized to PCBP1 L100Q. All dots are semitransparent black, resulting in a gray appearance when on their own and a blacker appearance when overlapping. **f** Distribution of reads between the indicated RNA types for PCBP1 and PCBP1 mutants. **g** Binding to the indicated RNA types on a per-protein or per-cell basis. **h** PCBP1 shifts from the nucleus to the cytoplasm in PCBP1 L100P mutants. The experiment was performed twice with similar results. Scale bar approximate. **i**–**l** PCBP1 and L100P/Q PCBP1 mutant cross-linking at the indicated RNAs. Signal was smoothed by fitting to a Gaussian kernel to enable visualization. The left column is reads per million; the middle, cross-links per billion proteins; the right, cross-links per cell. Dotted line, intron; thin solid line, UTR; thick solid line, CDS; arrows, direction of CDS. **m** DESeq2 estimates of RNA abundance between HCT116 cells expressing similar levels of L100Q and WT PCBP1, and with endogenous PCBP1 knocked-down by shRNA. **n** GO terms enriched in RNAs with increased (above the dotted line) or decreased (below the dotted line) RNA abundance in cells expressing mutant or wild-type RBPs. Terms are FDR < 0.15 across at least one cell type.

easyCLIP libraries were sequenced for normal and mutant forms of PCBP1, A1CF, KHDRBS2, FUBP1, RPL5, RARS2, and SMAD4. The effect of the recurrent missense mutations on cross-linking to individual RNAs (Fig. 6g) and RNA categories (Fig. 6h) was evaluated, with PCBP1 discussed separately (Fig. 7). On a per-read basis, differences were generally slight between RNA categories (aside from PCBP1), but there were numerous differences in individual RNAs (Fig. 6g and Supplementary Data 7). While the mutant vs WT changes induced between proteins were not generally correlated (highest *R*^2^ = 0.02), the lncRNA *PURPL* (p53 upregulated regulator of p53 levels^30^) was one of the five most significant changes for both KHDRBS2 and A1CF (Fig. 6g, i, j).

### L100 mutations of PCBP1

PCBP1 has recurrent missense mutations in L100, commonly L100Q, seen most frequently in colon cancer^16,31^ (Figs. 1f and 7a). PCBP1 is highly multifunctional beyond RNA binding^32,33^ and was expected to cross-link less than the average RBP, which was true (Fig. 7b–d). To test if cross-linking was specific, GxxG loop mutations were introduced in all KH domains of PCBP1, which remove the affinity of KH domains for RNA^34^. “GxxG PCBP1” no longer cross-linked (<0.01%, Fig. 7b, c). The first and second KH domains of the closely related PCBP2 form an intramolecular dimer, in which the β1 and α3 elements of both KH1 and KH2 bury hydrophobic residues against the other domain^35^. L100, in β1 of KH2, is part of this dimerization surface^35^, suggesting that the L100 mutants might alter conformation to impair association with RNA. Surprisingly, the opposite effect was observed: PCBP1^L100P/Q^ was threefold more cross-linked to RNA (Fig. 7b, c). PCBP1^L100P/Q^ was dramatically destabilized (Fig. 7e and Supplementary Fig. 23A). Expressing PCBP1 from a vector containing an upstream open-reading frame (ORF) (uORF) that lowered expression to below that of PCBP1^L100P/Q^ (Fig. 7b, c) did not equalize the cross-link rate to the level observed with the L100 mutants (Fig. 7b, c), ruling out expression levels as the cause of differential RNA binding. Interestingly, when the entire KH domain-containing L100 (KH2) was removed, cross-linking was approximately the same as WT (Fig. 7b–d), yet ∆KH2 PCBP1 was also destabilized (Fig. 7e and Supplementary Fig. 23A). easyCLIP thus demonstrated that the cancer-associated PCBP1^L100P/Q^ quantitatively increased RNA binding.

easyCLIP-seq data demonstrated that, in addition to enhancing RNA binding compared to WT, mutant PCBP1^L100P/Q^ bound different types of RNAs. For example, PCBP1^L100P/Q^ had a much smaller fraction of reads mapping to snRNA than WT (Fig. 7f), and PCBP1^L100P/Q^ greatly increased its association with mRNA per protein (Fig. 7g). It was therefore hypothesized that PCBP1^L100P/Q^ might be more cytoplasmic than WT PCBP1. This was confirmed by microscopy (Supplementary Fig. 23b and Fig. 7h). The quantifications done by easyCLIP enabled three different views of RNA–protein interactions (Fig. 7i–l). Cross-linking to snRNA by PCBP1^L100P^ is reduced per protein, but on a per-cell basis collapses almost completely (Fig. 7g, k). Although mutant PCBP1 interacts more often with mRNA per protein, per cell it is more similar (Fig. 7g, i, j). Altogether, these data highlight the complexity of RNA–protein interactions, and the importance of analyzing CLIP-seq data beyond traditional read count distributions.

To further explore the impacts of mutant RBPs in cancer, we analyzed the transcriptomic effects of recurrent missense mutations in PCBP1 and KHDRBS2 (Supplementary Fig. 24 and Supplementary Data 8). Both mutants led to >1000 RNAs changing levels at least 1.4-fold at FDR < 0.05 (Supplementary Fig. 24E, F). PCBP1 downregulation promotes tumorigenesis by decreased expression of *CDKN1A*^36^. Interestingly, PCBP1^L100Q^ bound more to the *CDKN1A* 3′-untranslated region (UTR) than WT PCBP1, and *CDKN1A* was the eighth most significantly downregulated RNA in PCBP1^L100Q^-expressing cells (Fig. 7i, m). This could not be explained by simple differences in PCBP1 protein abundance (Supplementary Fig. 24A). RNAs significantly upregulated by PCBP1^L100Q^ were enriched for GO terms involving cell division and extracellular matrix organization, while downregulated RNAs were highly enriched for genes involved in cell–cell adhesion (Fig. 7n). RNAs with a 3′-UTR PCBP1 peak location were more likely to be destabilized in response to PCBP1^L100Q^ expression (Supplementary Fig. 25A). Within the RNAs whose levels were decreased with PCBP1^L100Q^ that also display increased PCBP1^L100Q^ association (Supplementary Fig. 25B) were 32 cell adhesion genes, nearly all having 3′-UTR peaks with increased PCBP1^L100Q^ binding (Supplementary Fig. 25C, D). These results are consistent with a possible model in which L100P/Q might contribute to tumorigenesis via increased binding to the 3′-UTR of the RNAs that encode specific cell adhesion proteins, causing their destabilization.

## Discussion

Here, we present easyCLIP as a refinement of the CLIP method that may be useful for quantifying RNA per-protein cross-link rates and for production of CLIP-seq libraries. It introduces a quantitative non-isotopic approach for absolute quantification designed to facilitate comparison between CLIP datasets obtained in any laboratory and to provide direct visualization of the success of library preparation steps. easyCLIP is designed to help address a challenge in conventional CLIP protocols in which a time-consuming workflow is performed without ready availability of visual quality verification at key steps. Like all CLIP approaches, easyCLIP relies on UV cross-linking as a proxy for in vivo associations^10^. easyCLIP allows multiplexing based on two adapters and determines ligation efficiency, two features that streamline the workflow and enable its quantitative robustness. Finally, on a practical level, easyCLIP’s low technical experimental failure rate and non-isotopic labelling features are designed to increase the general usability of CLIP-seq.

Limitations of the method include the fact that some proteins may shift their RNA-binding properties under different conditions^37,38^, complementary DNAs terminating with an RT break may be lost, ncRNAs like tRNA may have different cross-link/ligation efficiencies, and that variations in complex cross-link efficiency mean that 2% vs 5% cross-link rates in two different RBPs might both reflect 100% occupancy. Cross-link numbers from transient expression may also differ from those obtained by other expression methods. It would be difficult to compare the easyCLIP cutoff of 0.1% with statistical enrichment cutoffs in tandem mass spectrometry experiments used to determine RBPs, but RBPs identified via such methods may have cross-link rates in the <1–10% range^5^, consistent with similar effective cutoffs. easyCLIP suggests that there is some overlap between RBPs and non-RBPs in cross-link rate, which poses challenges for experiments unpaired with sequencing. Using both sequencing and cross-link rates may therefore be more useful than using either alone.

easyCLIP data suggest that a UV cross-link rate of >0.1% may be a feature of RBPs that is uncommon for non-RBPs. In some non-RBP cases, a rate may be near 0.1% due to technical artifact; however, sequencing the CLIP libraries will reveal these cases. Random non-RBPs have distinct binding profiles in RNA and are not easily distinguished from RBPs based on sequencing alone, although certain features, such as a 3:1 exon:intron ratio, are characteristic of non-RBPs. easyCLIP enables the definition of nonrandom interactions with RNA by reference to random non-RBPs and doing so better matches the known biology of RBPs.

In three cases, recurrent cancer-associated missense mutations in RBPs increased binding to RNA: KHDRBS2, A1CF, and PCBP1. Although these mutations are not individually highly frequent, our data suggest that they, and others in the long tail of cancer mutations, have molecular phenotypes. PCBP1 easyCLIP results are consistent with a model where the L100P/Q mutations impair the stabilizing effect of KH2 and have a gain of function for KH2 with regards to location and RNA binding. PCBP1 protein is often downregulated in cancer, which aids in tumorigenesis^39^. Data here suggest that L100P/Q mutations may contribute to tumorigenesis at least partly by destabilizing PCBP1. However, PCBP1^L100P/Q^ is primarily observed at high frequency in bowel adenocarcinoma, raising the question as to the mechanism of its potential cell-type-enriched mutagenesis and possible functional impacts. PCBP1 has been proposed to suppress tumors by binding mRNA and stabilizing tumor suppressor mRNAs, repressing translation of oncogenic mRNAs, and inhibiting oncogenic splicing^39^. In this regard, cell–cell adhesion genes and cell cycle genes appear especially affected by PCBP1^L100Q^. To our knowledge, data presented here are the first demonstration that a disease-associated mutation in an RBP results in increased RNA association. easyCLIP helped identify these alterations and may be helpful in applying CLIP to quantify RNA cross-link rates per molecule of protein in additional future studies, including those examining RNA–protein dynamics in response to an array of stimuli.

## Methods

### Cultures

*Escherichia coli* BL21 cultures used to express recombinant protein were grown in standard LB broth at 37 °C with appropriate antibiotics. HEK293T (Takara Bio) and A375 (ATCC) cells were grown in Dulbecco’s modified Eagle’s medium (DMEM) media (Thermo Fisher, #11995-065) with 10% fetal bovine serum (FBS) and 1% penicillin–streptomycin at 37 °C under 5% CO_2_. HCT116 cells (ATCC) were grown in McCoy’s 5A (modified) medium (Thermo Fisher, #16600108), supplemented with 10% FBS and 1% penicillin–streptomycin, and grown at 37 °C under 5% CO_2_. HepG2 cells (ATCC) were grown in Eagle’s minimum essential medium (ATCC 30-2003), supplemented with 10% FBS and 1% penicillin–streptomycin, and grown at 37 °C under 5% CO_2_.

### L5 linker labelling

IRDye 680RD DBCO (0.5 mg) (LI-COR, 429 nmol) was resuspended in 42.9 µL phosphate-buffered saline (PBS) for a concentration of 10 mM. The L5 linkers (Azide-DNA-RNA oligonucleotides) were ordered from IDT (Integrated DNA Technologies) and resuspended in PBS. Oligonucleotides were run through a Zymo RNA-clean-and-concentrator kit (purification was required for labelling), using ~7 µg oligonucleotide per column, and eluting at ~0.5 mg/mL (~40 µM) in water. Before binding to columns, we added ethanol to a final concentration of 67% instead of the 50% recommended by the manufacturer. During column purifications, washes were performed using an 85% ethanol in water solution made fresh each time, in place of the kit’s wash buffer. Five microliters of 10 mM dye (~50 nmol) was added to 10–150 µg purified oligonucleotide (~1–12 nmol) in PBS for a total volume of 200 µL and reacted for 2 h at 37 °C. Oligonucleotides were then run again through a Zymo clean-up kit and eluted in water. Concentrations were determined by *A*_260_ ratio using an approximate *ε* = 368,050 M^−1^. Oligonucleotides were diluted to 10 nM in ligation buffer (50 mM Tris pH 7.5, 10 mM MgCl_2_, 16.7% PEG400), 1 µL was blotted onto a nylon membrane, and fluorescence was measured in an Odyssey CLx machine (LI-COR). This was typically ~15,000 fluorescence units/fmol for full labelling.

### Western blot protein quantification

Following the method of Janes^40^, purified gluthathione *S*-transferase (GST)-tagged protein standards were run alongside the samples to be quantified. Purified GST-hnRNPC2 and purchased FBL (Prospec, cat. enz-566) were diluted in protein dilution buffer (0.5× PBS, 0–5% glycerol, 0.05% Tween-20, 0.15 mg/mL BSA) to 200 ng/µL. Twofold dilutions down from 20 to 100 ng/µL were made for a total of eight concentrations; this solution was then delivered as 14 µL aliquots to multiple striptube aliquots and frozen at −80 °C. When running gels, 10 µL from each concentration was combined with 10 µL loading buffer (3.6× NuPAGE loading buffer with 10% β-mercaptoethanol), heated at 75 °C for 15 min, and loaded on a 4–12% NuPAGE gel. Standards were therefore present at ~3–2000 ng per lane. Immunoblotting against the HA epitope was performed with 1:3000 αHA conjugated to Alexa Fluor 488 and incubating for 1 h at room temperature in PBS blocking buffer (LI-COR); images were taken in a GE Typhoon scanner (532 nm laser, 526SP filter, 500 PMT, 200 µm resolution). When small aliquots of immunopurification beads were loaded on a gel, BSA was first added to 0.2 mg/mL to prevent absorption. Primary antibodies were diluted 1:1000 and secondary antibodies diluted 1:3000 for immunoblots, including nonquantitative immunoblots, unless otherwise noted.

### Creation of cross-linked hnRNP C standard

Four replicates of 906–1600 µg of HCT116 lysate from cross-linked cells were added to ~20 µL Protein G Dynabeads (Thermo Fisher, cat. #10003D) coupled with 25 µL (5 µg) anti-hnRNP C (4F4) antibody per replicate. Immunoprecipitation was carried out at 4 °C for ~1 h, followed by the standard easyCLIP protocol for cross-link rate determination. The RNAse digestion was performed with half of the samples treated with 0.1 U/µL RNAse ONE for 10 min, and the other half of the samples were treated with 0.05 U/µL RNAse ONE for ~5 min. The polynucleotide kinase (PNK) reaction was 14 min at 37 °C. The ligation was performed overnight (17 h) with 20 pmol L5 (barcode 23) and 2 µL high concentration of T4 RNA ligase (NEB). Samples were combined, and ~20 aliquots comprising 2.5% of the beads (~10 ng hnRNP C each, ~400 ng total purified) in ~15 µL 1.6× NuPAGE buffer were frozen in dry ice and kept long term at −80 °C. Immunoblotting was performed with ~1:3000 αhnRNP C conjugated to AF790 (Santa Cruz Biotechnology, sc-32308 AF790), which is visible on the 800 nm channel in a LI-COR Odyssey scanner, in PBS blocking buffer (LI-COR) for ~1 h at room temperature.

### Sequencing library creation: hnRNP C and FBL

HEK293T cells were grown to 30–90% confluency in petri dishes in DMEM with 10% FBS, media were removed by vacuum, cells were washed with 4 °C PBS, and UV cross-linked (254 nm) in 10 or 15 cm plates in a Stratalinker at 0.3 J/cm^2^. After cross-linking, 1 mL 4 °C lysis buffer (15 cm plates) or 0.5 mL lysis buffer (10 cm plates) was added to each plate, cells were harvested with a rubber spatula, and frozen in dry ice. CLIP lysis buffer was as in Zarnegar et al.^23^, except the concentration of Triton X-100 was 1% (see Supplementary Data 1 for all buffers used for CLIP). For each hnRNP C replicate, 4 µg hnRNP C1/C2 antibody (4F4, Santa Cruz Biochnology #sc-32308) and 20 µL Dynabeads Protein G for immunoprecipitation (Thermo Fisher, #10003D) were coupled for 1 h at room temperature before adding 600 µg of clarified HEK293T lysate and immunopurifying at 4 °C for 45–60 min. For FBL, two replicates of 4 mg clarified lysate were combined with 20 µL Fibrillarin antibody (Bethyl, #A303-891A) and 20 µL Protein G Dynabeads; immunopurification was at 4 °C for 1 h. The easyCLIP assay was performed as described in Supplementary Data 1.

### Cloning

Cloning primers are included in Supplementary Data 1 (“cloning primers” tab). Stitching reactions were performed with NEBuilder HiFi DNA Assembly (NEB, cat. # E262L) into pLEX-based vectors.

### Figure 2 cell culture

hnRNP C and FBL were purified using antibodies to the endogenous protein (Santa Cruz Biotechnology sc-32308, Bethyl A303-891A), using a >50% confluent 10 cm or 15 cm plate with HEK293T cells per replicate. RBFOX2 was purified using antibodies to the endogenous protein (Bethyl, A300-864A) and 20 million HEK293T cells. PCBP1 was stably integrated outside its genomic locus in HCT116 cells (harvesting one ~70% confluent 15 cm plate per replicate). The others were transiently transfected into HEK293T cells growing in 10 cm plates (~2 × 10^7^ cells) with a pLEX vector bearing a uORF to lower expression. Expression levels were low compared to endogenous protein (Supplementary Fig. 7).

### Figure 5 cell culture

Proteins included in Fig. 2 were expressed and purified in the same way. PCBP1 was stably integrated outside its genomic locus in HCT116 cells (harvesting one ~70% confluent 15 cm plate per replicate). The recurrently mutated proteins were transiently transfected into HEK293T cells growing in 10 cm plates (~2 × 10^7^ cells) or 15 cm plates (~3 × 10^7^ cells) with either a pLEX vector bearing a uORF to lower expression or a vector modified from pLEX to remove vector sequences to boost copy number, increase expression, or form viral particles. HA-STAU1 and randomly selected non-RBPs were expressed from a pLEX vector. Expression was 18–28 h before harvesting. WT and mutant proteins were always expressed, harvested, and processed together. Expression levels were low compared to endogenous protein (Supplementary Fig. 7).

### easyCLIP: library creation

The full easyCLIP protocol and all buffers are described in Supplementary Data 1. After harvesting, cells were thawed and lyzed with a microtip sonicator six times for 5 s each (10% power), with samples cooled by placement in dry ice between sonications. Lysates were then clarified by spinning at 14 k.r.c.f. for 10 min at 4 °C and the supernatant was transferred to a new tube. Concentrations were determined using bicinchoninic acid (see bicinchoninic acid section). To visualize protein expression levels, 15 µg of clarified lysates were used for western blotting. For immunopurification, typically 20 µL of anti-HA beads per sample were washed with NT2 buffer and then with CLIP lysis buffer. Samples were diluted to 1–4 mg/mL during immunopurification, typically ~2 mg/mL. Immunopurification was 40 min to 1 h at 4 °C. Samples were then washed once with high stringency (H. Str.) buffer (10 min), high salt (H. Salt) buffer (10 min), low salt buffer, and finally NT2 buffer, each with 1 mL. Samples were then stepped down with another wash to ~200 µL NT2 buffer. RNAse digestion was performed by diluting 2 µL of 100 U/µL RNAse ONE to 1 U/µL in NT2 buffer, then diluting this to 0.025 U/µL in NT2 buffer with 16% PEG, and adding 60 µL of this to each sample. The digestion was performed for 8–12 min at 30 °C with intermittent shaking. The digestion mixture was removed from the beads and 1 mL H. Str. Buffer was added. Samples were then washed twice with 1 mL NT2 buffer before being stepped down to ~200 µL NT2 buffer. Samples were then processed in the order (1) kinase, (2) 5′ ligation, (3) L5 barcodes combined, (4) phosphatase, and (5) 3′ ligation, or in the order (1) phosphatase, (2) 3′ ligation, (3) L3 barcodes combined, (4) kinase, and (5) 5′ ligation. Processing details and oligonucleotide sequences are in Supplementary Data 1. In either case, all samples were typically combined before being loaded into a single lane of a 4–12% NuPAGE Bis-Tris gel, run at 200 V for ~45 min, and transferred to nitrocellulose at 400–500 mA for ~25 min. Membranes were then placed in PBS and immediately imaged in an Odyssey CLx machine. Membranes were cut using scalpels and put in 375 µL PK buffer with 25 µL Proteinase K and incubated for 40–60 min with shaking at 45–55 °C. In some cases, 2 µL of extracted RNA was then spotted on nylon and imaged. PK mixtures were added directly to 20 µL oligonucleotide(dT) beads and mixed at room temperature for 20 min. Alternatively, 2 M KCl was added and SDS was spun out, then 20 µL oligonucleotide(dT) beads were added, and the samples were mixed at 4 °C for 20 min. Beads were washed once with biotin IP buffer, once with NT2 buffer, transferred to a PCR tube, nd then washed 3–4 times with PBS buffer. Samples were eluted in 14.4 water with 15 pmol RT primer by heating at 95 °C for 3 min and transferred to a new tube. RT was performed by incubating for 40 min at 53 °C and 10 min at 55 °C, or in some cases for 40 min at 53 °C only. RT product was then used directly for PCR as described in Supplementary Data 1. A protocol describing library preparation and cross-link rate determination can be found at Protocol Exchange^41^.

### Ligation efficiency test by protein shift

The ligation efficiency test with hnRNP C was performed in three replicates. hnRNP C was purified by incubating 600 µg of clarified HEK293T lysate with 4 µg anti-hnRNP C1/C2 antibody for 1.5 h at 4 °C^23^. Beads were RNAse digested and dephosphorylated, before being split 2:1. The split corresponding to 200 µg lysate was PNK phosphorylated and 5′ ligated as described in the easyCLIP protocol. The split corresponding to 400 µg was 3′ ligated, before being split in half. One 3′ ligated split was PNK phosphorylated and 5′ ligated as described in the easyCLIP protocol. All samples were then run on a 4–12% SDS-PAGE gel (NuPAGE), transferred to nitrocellulose, and visualized. The amount of RNA that was neither 5′ nor 3′ ligated was determined by the following reasoning. First, let *P*5 be the probability of a 5′ ligation, and *P*3 be the probability of a 3′ ligation. Let *a* = RNA with no ligation; *b* = RNA with a 3′ ligation only; *c* = RNA with a 5′ ligation only; and *d* = RNA with a 5′ and 3′ ligation. Let *T* = the total amount of RNA. It follows that:

1 $$b \times c = (T \times P3(1 - P5))\times(T \times P5(1 - P3))$$

2 $$a \times d = (T \times(1 - P5)(1 - P3))\times(T \times P5 \times P3)$$

Rearranging terms shows that *a* × *d* = *b* × *c*. Since *d*, *b*, and *c* are determined by direct visualization of fluorescence, it follows that the RNA with no ligation (*a*) is also known.

### CLIP analysis: peak location finding

Scripts used for CLIP analysis are available at github.com/dfporter/easyCLIP. For the peak locations used for motif finding in Fig. 2f, peaks were defined by averaging signal across the genomic locus. For each RNA, reads spanning the genomic locus were converted into an array with the length of the genomic locus and each value representing the count of 5′ read ends mapping to that position. The values were smoothed by convolution using a box with length 50 for loci of at least 2000 nucleotides, length 20 for 20–2000 nucleotides, and length 10 for <200 nucleotides. Artifacts were removed by discarding an RNA if there existed a 2-nucleotide interval in the 100 nucleotides centered around (and including) the peak that contains >80% of the total signal in that 100 nucleotide window. If reads mapped to multiple RNAs, but only one was an exon, reads were assigned to the exon. If reads overlapped with the exons of multiple RNAs, they were assigned a priority in the order rRNA, snRNA, small Cajal body-associated RNA, snoRNA, tRNA, and mRNA. If this priority list did not result in a single RNA having priority, the reads were considered ambiguous and not used for peak finding. If the smoothed array had a single maximum, it was taken to be the peak location. If there were multiple maxima (equal heights) and no maxima had more than a two-nucleotide gap from another maxima, the peak was taken as the average position between the first and last maxima. If any maximum was more than two nucleotides from another maximum, the RNA was considered to have no peak.

### CLIP analysis: statistics

Inputs to statistical analysis were either reads per million or reads per ten billion proteins, both treated the same. To speed up analysis, for the randomly selected non-RBPs constituting background, if a replicate had no reads it was assigned one-tenth the minimum positive count present in that dataset (i.e., if a dataset had one million reads, zeros were replaced with 0.1 reads per million). The average count across replicates for each protein was determined, resulting in a sample of eight values taken from the null distribution (one for each of the proteins CDK4, CHMP3, DCTN6, ETS2, IDE, ITPA, TPGS2, and UBA2). If *σ*^2^/*µ* was >2 for these samples, they were fit to a negative binomial, and they were fit to a Poisson if *σ*^2^/*µ* was <2. *P* values were calculated accordingly before finally adjusting all *P* values for each protein by the Benjamini–Hochberg method into FDR equivalents.

Additional methods are described in Supplementary Data 1 and in Supplementary Methods.

### Reporting summary

Further information on research design is available in the Nature Research Reporting Summary linked to this article.

## Supplementary information

Supplementary Information

Reporting Summary

Supplementary Datas 1

Supplementary Data 2

Supplementary Data 3

Supplementary Data 4

Supplementary Data 5

Supplementary Data 6

Supplementary Data 7

Supplementary Data 8

Description of Additional Supplementary Files

## Data Availability

Plasmids will be provided upon request. High-throughput sequencing data are given under the accessions “GSE154168”, “GSE162366”, and “GSE131210”. Source data are provided with this paper.

## Source data

Source Data

## Code availability

Software is available under github.com/dfporter/easyCLIP.
