# Supplementary material for: easyCLIP analysis of RNA-protein interactions incorporating absolute quantification
Source: Nat Commun. 2021 Mar 10;12:1569. doi: 10.1038/s41467-021-21623-4 (PMC7946914; doi:10.1038/s41467-021-21623-4)
Supplement: Supplementary file 1 — Supplementary Information [file 41467_2021_21623_MOESM1_ESM.pdf]

Supplementary Info

easyCLIP Analysis of RNA-Protein Interactions Incorporating Absolute Quantification

Authors: Douglas F. Porter[1,2], Weili Miao[1,2], Xue Yang[1,2], Grant A. Goda[3,4], Andrew L. Ji[1,2], Laura K. H. Donohue[1,5], Maria Aleman[3], Daniel Dominguez[3,6], Paul A. Khavari[1,2]*

Affiliations:

[1]Program in Epithelial Biology, Stanford University, Stanford, CA 94305

[2]Stanford Program in Cancer Biology, Stanford University, Stanford, CA 94305

[3]Department of Pharmacology, University of North Carolina at Chapel Hill, Chapel Hill, North Carolina 27599, United States

[4]Department of Chemistry, University of North Carolina at Chapel Hill, Chapel Hill, North Carolina 27599, United States

[5]Department of Genetics, Stanford University, Stanford, CA 94305

[6]Lineberger Comprehensive Cancer Center, University of North Carolina at Chapel Hill, Chapel Hill, North Carolina 27599, United States

*Correspondence to: khavari@stanford.edu

Supplementary Methods

Fluorescence polarization expression constructs

Modified pGEX6 bacterial expression (GE Healthcare) constructs harboring: HIS-GST-PCBP1 or HIS-GST-PCBP1 L100P (Human PCBP1 full coding region was used (UniprotID:Q15365)) HIS-GST-A1CF or HIS-GST-A1CF E34K (Human A1CF residues 1-325 (UniprotID:Q9NQ94) were used), HIS-GST-HALO-KHDRBS2 or HIS-GST-HALO-KHDRBS2 R168C (Human KHDRBS2 residues 1-200 (UniprotID:Q5VWX1)).

Protein purification for fluorescence polarization

Rosetta Cells (MilliporeSigma) were transformed and cultures were grown in LB broth until the optical density reached ~0.6 (typically 1-2 liters per transformant). Cultures were brought to 16° and induced with Isopropyl β-d-1-thiogalactopyranoside overnight (~16 hrs). Cells were then harvested and lysed in lysis buffer (25 mM Tris-HCl, 200 mM NaCl, 3 mM MgCl2, 500 units/per 1L culture Benzonase Nuclease, 2.5 mM phenylmethylsulfonyl fluoride). Cell pellets were then sonicated, incubated for 30 minutes on ice, and centrifuged at 37 krcf for 35 minutes at 4°. Supernatants were passed through a 0.45 micrometer filter and GST-tagged proteins were purified using GST-trap FF columns (GE) on AKTA Pure System or using glutathione-conjugated agarose resin (Pierce) in batch. Protein was eluted with elution buffer (50 mM Tris-HCl, 20 mM glutathione, pH 7.0). Protein was dialyzed against 4 L of storage buffer (20 mM HEPES, 5% glycerol, 100 mM NaCl) and concentrated to ~50 µM. Purity was assessed by PAGE and Coomassie stain.

Fluorescence polarization assay

RNA oligos labeled at the 3' end with 6-FAM were purchased from Integrated DNA Technologies. The RNA sequences used were 5'-CCCCCCCCCCCCCCCC-6FAM-3' (PCBP1), 5'-AUUAAAUUAAAUUU-6FAM-3' (A1CF and KHDRBS2), and 5'-NNNNNNNNNNNNNNNN-6FAM-3' (CTRL RNA). The preferred binding motifs were derived from Dominguez *et al.*[1] PCBP1 RNA was diluted to 50 nM in PCBP binding buffer (50 mM Sodium Acetate, 150 mM Magnesium Acetate, 5 mM Glutathione, 0.01% triton and 10 µg/mL BSA at pH 6.5). KHDRBS2 and A1CF RNA were diluted to 50 nM in binding buffer (50 mM Sodium Acetate, 150 mM Magnesium Acetate, 5 mM Glutathione, 0.01% Triton and 10 µg/mL BSA at pH 7.4). Recombinant proteins were serially diluted in respective binding buffers as indicated above. Proteins were incubated with respective cognate RNA or control RNA (5 nM final) for 15 minutes at 4° in a reaction volume of 35 µL in 384-well plate format. Fluorescence polarization was read with a PHERAstar plate reader (BMG Labtech) at 26-27°. Binding experiments were performed in duplicate and repeated 3 times, except PCBP1 binding was repeated twice. Binding constants were derived by fitting data a 4-parameter logistic curve.

RNA-seq Library Preparation

Lentivirus (pLEX-based) expressing wild-type or R168C KHDRBS2, with a uORF to lower expression, was produced as described for shRNA production. Similarly, lentivirus expressing wild-type or R429C FUBP1 with a uORF, PCBP1 wild-type with a uORF, and both PCBP1 wild-type and PCBP1 L100Q without a uORF were produced as described for shRNA production. A375 and HEK293T cells were grown in DMEM with 10% FBS.

HCT116 cells were grown in McCoy's 5A media with 10% FBS. 293T cells were sequentially infected with shRNA lentivirus (if any were used) targeting the endogenous 3'UTR, selected using Puromycin or Blasticidin for at least 3 days, then infected with lentivirus expressing protein or empty vector control (which lack the endogenous 3'UTR). HCT116 cells were infected with protein-expressing vector first, followed by shRNA, and harvested 3 days after shRNA infection without selection, as the essential nature of PCBP1 caused fluctuating expression levels with longer knock-downs. Qiagen RNeasy Mini Plus kit (Cat # 74134) was used to extract RNA, poly(A) libraries were constructed using NEBNext Ultra II RNA Library Prep Kit for Illumina, and libraries were sequenced on an Illumina NovaSeq 6000 using paired-end sequencing.

RNA-seq Analysis
RNA-seq libraries were sequenced on an Illumina NovaSeq PE150 at a depth of 25 million reads per sample. Paired end reads were mapped to the hg38 reference genome with GRCh38 Ensembl annotations using STAR aligner[2] (version 2.5.4b) followed by generation of genes by samples counts matrices with RSEM (version 1.3.0). BAM files generated from RSEM[3] were further analyzed with the featureCounts() function in Rsubread (v 1.32.4) to generate exons by samples counts matrices in R 3.5.1. Genes by counts matrices were further analyzed with the DESeq2 package[4] (v 1.24.0) in R 3.6.1 to calculate differential expression and associated p-values across samples. Each cell line was analyzed separately. Differential exon usage from exons by samples counts matrices was determined using the DEXSeq[5,6] (v 1.30.0) using recommend parameters based on tutorials available on Bioconductor. RNA-seq differential expression heatmaps were generated with the pheatmap package (v 1.0.12) in R using log2 normalized transcript counts using the function normTransform() in DESeq2. Gene ontology (GO) analysis was performed using DAVID v6.8[7] (Huang et al., 2009) on the top differentially expressed genes with adjusted p-value < 0.05 as calculated using DESeq2.

Virus infection with shRNA
Lentivirus was produced in Lenti-X 293Ts by transfecting 5 µg p8.91 vector, 1.6 µg pMDG vector, and 5 µg target vector into an >60% confluent 10 cm plate using Lipofectamine 3000 (ThermoFisher L3000015). Medium was changed after incubation overnight, and virus harvested after two and three days of expression. The two harvests were concentrated using Lenti-X Concentrator (Takara, 631231), combined and resuspended in 500 µL PBS. For infection, 500,000 HCT116 cells per well were seeded into 6 well plates. 30 µL virus (6% of the yield from a 10 cm plate) was added per well, followed by

polybrene. Media was changed the next morning. On the third day Puromycin was added, and selection performed for two days.

Virus infection for protein expression

Lentivirus was produced as described for shRNA production. 100,000 HCT116 cells were seeded per well of a 6-well plate, followed by 1-10 µL virus and polybrene. Puromycin was added on the third day after transfection and cells selected for two days.

Comparison with RBFOX2 eCLIP

RBFOX2 eCLIP replicates and input controls were download from GEO (GSE77629) as BigWig files, which were then converted to bedgraph files, and coordinates converted from hg19 to hg38 using liftOver. The few regions generating some problematic mapping in coordinate conversion were then identified and those regions were excluded from comparisons with easyCLIP. eCLIP files were then converted back to BigWig. Since the eCLIP files were in reads per million, signal from easyCLIP replicate bam files was normalized to per million before comparison.

eCLIP peaks were obtained from the published list, and we followed the authors in subsetting to peaks with SMInput normalized p-values (log10) above 8 and CLIPper p-values (log10) above 5. 1000 random eCLIP peaks were expanded by 1000 bp on each side of the peak; signal within each region was smoothed in 200 nt windows and evaluated by spearman correlation between replicates of easyCLIP, eCLIP and eCLIP input controls. For easyCLIP peaks, we subset to peaks with a gene-based P value (exon or intron) below 0.01. We expanded the peak position by 1000 bp on each side and subset to peaks with some position of easyCLIP signal with a read pileup density of at least 4 reads per million in that window. Spearman correlations were calculated the same as for eCLIP peaks.

Microscopy of transiently transfected cells

8-well plastic chamber slides ((Lab-Tek Permanox, Sigma #C7182) were coated with 0.01% poly-L-lysine (Sigma #P4707) for 15 minutes, then washed twice with PBS before use. HCT116 cells were plated in 24-well plates and grown for at least a day before transfection. 1 µg plasmid, 1 µL Lipofectamine 3000, and 2 µL P3000 reagent were mixed together in Opti-MEM in wells of a 96-well plate, and then added to HCT116 cells growing in 24-well plates. After six hours, the media was changed. The next day cells were moved to chamber slides and allowed to grow for at least another 24 hours before imaging. Cells were washed once with PBS, then fixed for 10 minutes in 4% formaldehyde (in PBS) at room temperature, rinsed three times with PBS, and then permeabilized with PBS containing 0.5% Triton X-100 and 10% goat serum. After permeabilization, cells were stained for at least 1 hour at room temperature with HA Tag Monoclonal Antibody 16B12 conjugated to Alexa Fluor 488 (ThermoFisher #A21287) at 1:250 dilution in PBS containing 0.05% Triton X-100 and 1% goat serum. After staining, cells were washed three times with PBS containing 0.05% Triton X-100, and the slide chamber removed.

After drying the cells by aspiration, one drop of DAPI mounting solution was added to each well and a coverslip was added and sealed with acetone.

AAVS1 microscopy of PCBP1 integrants
4-well plastic chamber slides (Lab-Tek Permanox, Sigma #C6932-1PAK) were coated with 0.01% poly-L-lysine (Sigma #P4707) for 15 minutes, then washed twice with PBS, left dry for 5-30 minutes, and then either stored under PBS or used immediately. HCT116 cells were plated at <20% confluency and grown at least 24 hours before staining. Cells were washed 1-2 times with PBS, then fixed for 10 minutes in 4% formaldehyde (in PBS) at room temperature, rinsed three times with PBS, and then permeabilized with PBS containing 0.5% Triton X-100 and 10% goat serum. After permeabilization, cells were stained for 1 hour at room temperature with HA Tag Monoclonal Antibody 16B12 conjugated to Alexa Fluor 488, ThermoFisher #A21287 at 1:200 dilution in PBS containing 0.05% Triton X-100 and 1% goat serum. After staining, cells were washed three times with PBS containing 0.05% Triton X-100, then 2-3 times in PBS without detergent, and the slide chamber removed. After letting the cells dry for a few minutes, one drop of DAPI mounting solution was added to each well and a coverslip was added and sealed with acetone.

AAVS1 integration
~2 µg repair template and ~1 µg Cas9/guide RNA plasmid were transfected using lipofectamine into 6-well plates containing ~300,000 cells each. Two days later, puromycin was added to 1 µg/mL and selection continued for at least 10 total days. To determine expression levels, 10 µg to 80 µg of clarified lysate in 1-8 µL of CLIP lysis buffer (typically 4 µL) was combined with 16 µL 1.6X LB (NuPAGE) and run on an SDS-PAGE gel. hnRNP C was immunoblotted using labelled anti-hnRNP C antibody (Santa Cruz, 798-conjugated) at 3 µL in 5-7 mL PBS blocking buffer (LI-COR), incubating for 30 minutes and washing with PBS for 20 minutes. To immunoblot for the HA tag, ~3 µL

Rabbit anti-HA (COVANCE) in 5-7 mL blocking buffer, followed by ~3 µL IR680 or IR800 labeled Goat anti-Rabbit (LI-COR) in 5-7 mL were used.

AAVS1 integrated FHH-tagged protein purification
15 µL anti-HA magnetic beads and 2-4 mg clarified lysate were used per immunopurification. Immunopurifications were carried out at 4° for 1 hour in 1 mL of CLIP lysis buffer.

GST-tagged protein constructs
pGEX-6P-1 vector was digested with BamHI and CSRP1-FLAG-HA was cloned in using In-Fusion (Takara). Amplification primers for CSRP1-FLAG-HA were:

Left primer	GGGGCCCCTGGGATCCATG CCGAACTGGGGAG
Right primer	GATGCGGCCGCTCGAGTCATGAACCTGCAGCATAGTCAGGCACATC

The GST moiety (and protease site) is 231 amino acids (26.8 kDa), and CSRP1-FLAG-HA is 217 amino acids (23.2 kDa), for a 448 amino acid (50 kDa) construct. This resulting sequence is given below, with CSRP1-FLAG-HA underlined (* denotes stop):

MSPILGYWKIKGLVQPTRLLLEYLEEKYEEHLYERDEGDKWRNKKFELGLEFPNLPYYI
DGDVKLTQSMAIIRYIADKHNMLGGCPKERAEISMLEGAVLDIRYGVSRIAYSKDFETLK
VDFLSKLPEMLKMFEDRLCHKTYLNGDHVTHPDFMLYDALDVVLYMDPMCLDAFPKL
VCFKKRIEAIPQIDKYLKSSKYIAWPLQGWQATFGGGDHPPKSDLEVLFQGPLGSMPN
WGGGKKCGVCQKTVYFAEEVQCEGNSFHKSCFLCMVCKKNLDSTTVAVHGEEIYCK
SCYGKKYGPKGYGYGQGAGTLSTDKGESLGIKHEEAPGHRPTTNPNASKFAQKIGGS
ERCPRCSQAVYAAEKVIGAGKSWHKACFRCAKCGKGLESTTLADKDGEIYCKGCYAK
NFGPKGFGFGQGAGALVHSELEDYKDDDDKAGYPYDVPDYAAGS*

A second construct, GST-FLAG-HA-HIS-CSRP1 (GST-FHH-CSRP1) was created in order to move the HA tag into the interior of the protein so that degradation of the protein at the ends could not lead to confusion. The resulting 461 amino acid (51585 Da) construct is below, with the FHH tag in bold and CSRP1 underlined:

MSPILGYWKIKGLVQPTRLLLEYLEEKYEEHLYERDEGDKWRNKKFELGLEFPNLPYYI
DGDVKLTQSMAIIRYIADKHNMLGGCPKERAEISMLEGAVLDIRYGVSRIAYSKDFETLK
VDFLSKLPEMLKMFEDRLCHKTYLNGDHVTHPDFMLYDALDVVLYMDPMCLDAFPKL
VCFKKRIEAIPQIDKYLKSSKYIAWPLQGWQATFGGGDHPPKSDLEVLFQGPLGS**DYK**
DDDDKAGYPYDVPDYAAGSHHHHHHGSMPNWGGGKKCGVCQKTVYFAEEVQCEG
NSFHKSCFLCMVCKKNLDSTTVAVHGEEIYCKSCYGKKYGPKGYGYGQGAGTLSTDK
GESLGIKHEEAPGHRPTTNPNASKFAQKIGGSERCPRCSQAVYAAEKVIGAGKSWHK

ACFRCAKCGKGLESTTLADKDGEIYCKGCYAKNFGPKGFGFGQGAGALVHSELERPH
RD*

GST-FHH-CSRP1 was characterized and employed the same as GST-CSRP1-FLAG-HA.

The GST-hnRNP C construct (54 kDa) was cloned into the same vector but did not include HA or FLAG tags. The resulting sequence is below:

MSPILGYWKIKGLVQPTRLLLEYLEEKYEEHLYERDEGDKWRNKKFELGLEFPNLPYYI
DGDVKLTQSMAIIRYIADKHNMLGGCPKERAEISMLEGAVLDIRYGVSRIAYSKDFETLK
VDFLSKLPEMLKMFEDRLCHKTYLNGDHVTHPDFMLYDALDVVLYMDPMCLDAFPKL
VCFKKRIEAIPQIDKYLKSSKYIAWPLQGWQATFGGGDHPPKSDLEVLFQGPLGSMAS
NVTNKTDPRSMNSRVFIGNLNTLVVKKSDVEAIFSKYGKIVGCSVHKGFAFVQYVNER
NARAAVAGEDGRMIAGQVLDINLAAEPKVNRGKAGVKRSAAEMYGSVTEHPSPSPLL
SSSFDLDYDFQRDYYDRMYSYPARVPPPPPIARAVVPSKRQRVSGNTSRRGKSGFNS
KSGQRGSSKSGKLKGDDLQAIKKELTQIKQKVDSLLENLEKIEKEQSKQAVEMKNDKS
EEEQSSSSVKKDETNVKMESEGGADDSAEEGDLLDDDDNEDRGDDQLELIKDDEKEA
EEGEDDRDSANGEDDS*

GST-tagged protein purification
E. coli BL21 cultures transformed with pGEX-6P-1 were grown in 500 mL at 37° until OD600 ~0.8, at which time Isopropyl-1-thio-β-D-galactopyranoside (IPTG) was added to a final concentration of 0.5 mM, and cultures were grown for another ~1.5 h before harvesting. Cells were harvested by the method of S. Harper *et al.*[8], namely centrifuging at 4,000 rcf for 20 min at 4°, resuspending in ~50 mL LB, and centrifuging again at 4,000 rcf for 20 min at 4°. Cell pellets were frozen in dry ice until purification. When thawed, the cell pellet was resuspended in 20 mL of lysis buffer (50 mM Tris pH 8.0, 10 mM β–mercaptoethanol, 50 mM NaCl, 5 mM EDTA, 1% Triton X-100, Roche protease inhibitor, 5% glycerol). Lysozyme was added very approximately to ~1 mg/mL, pellet was frozen again in dry ice, then thawed in a water bath and lyzed by sonication. The lysate was clarified by centrifugation at ~21,000 rcf, 4°, for 15 min. 4 mL of 50% glutathione-agarose (Pierce) was washed with resin wash buffer (Dulbecco PBS with 10 mM β–mercaptoethanol), and then incubated at 4° in a 50 mL Falcon tube with clarified lysate for ~30 min before loading on a column. The column was washed with 50 mL of 4° wash buffer (Dulbecco PBS with 10 mM β–mercaptoethanol, 5% glycerol and Roche protease inhibitor). Samples were eluted in batch with three incubations at 4° with 1.5-2 mL elution buffer (100 mM Tris pH 8.0, 150 mM NaCl, 10 mM β–mercaptoethanol, 5% glycerol, 10 mM glutathione).

GST-tagged protein quantification
Following the method of K. Janes[9], BSA standards were run on a gel at 10, 5, 2.5, 1.3, 0.6, 0.3, and 0.15 µg, along with purified protein. Following the method of S. Luo *et al.*[10], gels were washed for 10 minutes in water, stained for 10 minutes with staining buffer (50% methanol, 10% acetic acid, 0.02% Coomaisse R250) at room temperature, followed by destaining for 10 minutes with destaining buffer (40% methanol, 7% acetic acid), and

washing twice for 10 minutes with water. A third wash was performed overnight. Protein was then visualized by scanning the 700 nm channel on a LI-COR Odyssey scanner. A hyperbolic curve of band fluorescence *vs* input protein weight was fit to BSA standards. Specifically, the parameters ‘a’ and ‘b’ in the equation $y = a*x/(b+x)$, where ‘x’ is protein weight and ‘y’ is fluorescence, were fit using least-squares regression. This curve was used to determine the concentration of purified protein.

BCA

For BSA standards, 105 µL PBS was combined with 20 µL BSA (2 mg/mL stock) and 3 µL lysis buffer for the highest concentration of BSA, and 115 µL PBS, 10 µL BSA, and 3 µL lysis buffer for the second highest concentration. For lysate samples, 3 µL lysate was combined with 125 µL PBS. For both standards and samples, serial dilutions were made by a factor of three into PBS with 0.024% lysis buffer. Duplicate wells were used for each sample. 25 µL of each well was transferred to a second 96-well plate and combined with 200 µL working reagent (Pierce BCA kit, 50:1 A:B). Plate was incubated for 20-30 minutes at 37°. Absorbance was measured at 562 nm.

FHH-hnRNP C F54A comparison

Tagged FHH-hnRNP C F54A could only be compared with FHH-hnRNP C by minimal region RNA because both purify the endogenous hnRNP C, which is heavily cross-linked in either case.

Histograms of binding frequency

For each protein, RNAs with no reads were removed before determining the histogram (hence the leftmost bin varies by dataset size). RNAs with no reads were not included in the histogram. RNAs that would be placed outside the rightmost bin were placed in the rightmost bin.

Fluorescence loss

20 µL of Streptavidin Dynabeads (ThermoFisher) per purification were washed three times with BIB, then combined with 2 µL of 5 µM biotin-anti-L5 RNA (10 pmol, ordered as /5BiosG/rUrArCrCrCrUrUrCrGrCrUrUrCrArCrArCrArCrArCrArArG from IDT, with an RNAse free HPLC purification). The oligonucleotide was captured for 20 minutes in 1 mL BIB, then washed with BIB, NT2, PBS (1X each) and resuspended in 50 µL BIB.

6.4 µL 2 M KCl was added to proteinase K-digested samples, and SDS was precipitated on ice for 15 minutes. SDS was spun out at 16 krcf for 10 minutes. The prepared Streptavidin Dynabeads with 10 pmol biotin-anti-L5 RNA oligonucleotide in 50 µL BIB were then added to PK reactions and diluted to a total volume of 1 mL with BIB. The purification was carried out at 4° for 20 minutes. Beads were washed three times with BIB, twice with PBS, and eluted for 2 minutes at 95° in 15-20 µL water with 100 nM biotin.

10X NT2 was added to 1X final concentration, and PEG to 16% final concentration. 1 µL 100 U/µL RNAse ONE was added and samples incubated for 40 minutes at 37°. RNAse ONE was inactivated by adding 10% SDS to 0.1%. Shift buffer was added to 1X (25 mM Tris pH 7.5, 10 mM $MgCl_2$, and 16% PEG400). 300-400 fmol labelled antisense oligos

were added and samples were processed further as described for the ligation efficiency test by anti-sense oligo shift.

Shift oligos:

αL5	/5AzideN/TACCCTTCGCTTCACACACACAAG	24 nt
αL3	/5AzideN/TTTTTCTGAACCGCTCTTCCGATCTCAG	28 nt

300-400 fmol labelled antisense oligonucleotides were added (max is ~500 fmol before signal cannot be quantified). The relative amount of shift oligonucleotide to input is important, as excessive oligonucleotide will create artifacts. Heat at 75° for 2 minutes, then let sample sit at room temperature for at least a minute. Create samples for two lanes of shift oligonucleotides at 300 fmol per lane (or however much was used to shift). Running the shift oligonucleotides at the same concentration used to shift is required to subtract background. Add 6X Ficoll/BPB buffer (15% Ficoll 400, 0.03% Bromophenol blue, 50 mM Tris pH 7.5) to 1X, but do not heat. For gel running buffer, add NaCl to 25 mM in 4° 0.5X TBE buffer. Samples were loaded on a 20% TBE gel and run gel 180V at 4° for one hour, replacing running buffer with 4° buffer every ~40 minutes. Finally, samples were transferred to nylon in 0.5X TBE buffer at 250 mA for 30 minutes.

Ligation efficiency test by RNA shift.
Samples of hnRNP C were prepared as normal for easyCLIP (Supplementary Data 1), and as described for the protein shift ligation efficiency test, up to the proteinase K extraction from nitrocellulose. To inactivate proteinase K, 6.4 µL 2M KCl per 400 µL of proteinase K extract was added, samples incubated at 4° for 15 minutes, and precipitated SDS removed by centrifugation at 16,000 krcf for 10 minutes at 4°.

Two sets of MyOne C1 Streptavidin beads were prepared, each using 13-20 µL MyOne C1 streptavidin beads per sample: one set for biotin purification and one for antisense oligonucleotide purification. Beads were washed three times with Biotin IP Buffer (BIB: 100 mM Tris pH 7.5, 1 M NaCl, 0.1% Tween-20, 1 mM EDTA). Those to be used for the biotin purification were then set aside until use. The set for anti-sense oligonucleotide purification were then incubated with 30 pmol anti-sense biotinylated oligonucleotide per µL resin in 1 mL BIB and rotated for 20 minutes at room temperature. Solution was removed and a second incubation with 15 pmol biotinylated oligonucleotide per µL resin was performed to ensure saturation. After incubation, anti-sense oligonucleotide beads were washed with BIB, NT2, PBS, and resuspended in 750 µL BIB. 50 µL of this bead solution was added to 400 µL BIB containing 20 nmol biotin and mixed. This solution was allowed to sit at room temperature for at least 5 minutes.

Proteinase K extract was bound to beads and incubated for 20 minutes at 4°. Supernatant was removed and beads were resuspended in 200 µL BIB, transferred to a PCR tube, rinsed with 200 µL NT2, washed with 200 µL PBS, and allowed to at least briefly reach

20-25°. After reaching room temperature, supernatant was removed and libraries eluted in 18 µL formamide at 65° for 2 minutes.

Ligation efficiency test by anti-sense oligonucleotide shift.
Beads were washed three times with BIB, twice with PBS, and eluted for 2 minutes at 95° in 15-20 µL water with 100 nM biotin. To this was added 10X NT2 to 1X final concentration, and PEG to 16% final concentration, then finally 1 µL 100 U/µL RNAse ONE. Mixture was incubated for 40 minutes at 37°. 10% SDS was added to a final concentration of 0.1% to inactivate RNAse ONE. Shift buffer was added to 1X (25 mM Tris pH 7.5, 10 mM $MgCl_2$, and 16% PEG400) final concentration. The volume was split in three or four if doing separate shifts.

300-400 fmol labelled antisense oligos were added (max is 500 fmol before signal cannot be quantified). The relative amount of shift oligo to input was important, as excessive oligo would create artifacts. Samples were heated to 75° for 2 minutes, then cooled to room temperature at -0.1°/s. 6X Ficoll/BPB buffer (15% Ficoll 400, 0.03% Bromophenol blue, 50 mM Tris pH 7.5) was added to 1X before loading on a gel. For gel running buffer, NaCl to was added to 25 mM in 4° 0.5X TBE buffer. Samples were loaded on a 20% TBE gel and run at 180V at 4° for ~1-3 hours, replacing running buffer with 4° buffer every ~40 minutes. Finally, samples were transferred to nylon in 0.5X TBE buffer at 250 mA for 30 minutes.

Recurrent missense mutations in RBPs
A few proteins were left off Fig 1D because we did not obtain data on them (e.g., BCLAF1).

CLIP analysis: genomes
The GRCh38 genome Gencode release 29 and features were obtained from:
ftp://ftp.ebi.ac.uk/pub/databases/gencode/Gencode_human/release_29/GRCh38.primary_assembly.genome.fa.gz
ftp://ftp.ebi.ac.uk/pub/databases/gencode/Gencode_human/gencode.v29.primary_assembly.annotation.gtf.gz.
The STAR index was built using --sjdbOverhang 75. When assigning reads to genes after STAR mapping, only GTF features with transcript support level tsl1 or tslNA were included.

Repetitive elements were handled in two ways: “repeats-first” or “separate”. The details of each approach are described in github.com/dfporter/easyCLIP/README_genome.md.

For “repeats-first” mapping, an alignment file was downloaded from http://www.repeatmasker.org/. This was parsed to extract representatives, which were placed in an artificial chromosome separated by poly(N), and a gtf file for each representative was generated. A STAR index was built with --genomeSAindexNbases 5. The parameter genomeSAindexNbases must be set well below the default of 14 or building will be very slow. When mapping to the repeats chromosome, --alignIntronMax 1 was used to prevent the insertion of introns by STAR. For “repeats-first mapping”, reads

were first mapped to a custom-built chromosome of repetitive elements using STAR and "--alignEndsType EndToEnd". Unmapped reads from this stage were then mapped to the regular genome using default parameters. Reads mapping the genome were filtered to remove multimapping reads and MAPQ < 10 reads.

For "separate" mapping, the method from RepEnrich2 was used[11], specifically RepEnrich2 from github.com/nerettilab/RepEnrich2. The RepEnrich2 method maps every read to a bowtie2 genome comprised of the genomic instances of each type of repeat. All reads were mapped using the RepEnrich2 method and, separately, using STAR to the genome in the same manner as "repeats-first". Reads mapping the genome were filtered to remove multimapping reads and MAPQ < 10 reads. After mapping, reads that mapped, *via* RepEnrich2, to rRNA, scRNA, snRNA, or tRNA were assigned to those elements (in that priority order). Reads not mapping to those elements, if they mapped uniquely to the genome by STAR, were assigned to the genome. Those reads not mapping uniquely to the genome, but which mapped *via* RepEnrich2 to an element other than the priority ncRNA (rRNA/scRNA/snRNA/tRNA), were then assigned to a repetitive element in a priority based on the number of instances of the given repeat element class in the genome. The "separate" mapping was used in general, with the some exceptions, including Fig. 2J-K, 6C-D, 7G and biotype analysis.

CLIP analysis: read processing
Custom Python scripts (github.com/dfporter/easyCLIP) were used for all analysis. Raw fastq files were split by L5 and L3 barcodes allowing one nucleotide mismatches to the expected barcodes. Mapping results from repetitive elements and the genome were combined, read mates removed, results converted to BED format, and PCR duplicates removed using the random hexamer UMI on the L5 adapter. Software packages samtools (v 1.1) and bedtools (v 2.27.1) were used during CLIP analysis.

CLIP analysis: read assignment
If reads mapped to multiple RNAs, but only one was an exon, reads were assigned to the exon. If reads overlapped with the exons of multiple RNAs, the reads were considered ambiguous. The strand was ignored for repetitive elements. Only transcripts with a "transcript_support_level" tag of "1" or "NA" (the latter is used for ncRNA) in the genomic annotation GTF was used. If a gene had multiple transcripts after filtering, the longest transcript (as in the longest genomic distance between the beginning of the first exon and the end of the last exon) was used.

CLIP analysis: EdgeR
EdgeR (v. 3.30.0) was run to compare the wild-type and mutant forms of RBPs. The design was "model.matrix(~batch+group)", where group denotes wild-type or mutant, and

batch denotes samples processed together. The functions glmQLFit and glmQLFTest were run with the default parameters, and outputs are in Supplementary Data 6.

FBL normalized snoRNA binding
For viewing FBL binding to an average snoRNA (Fig. 2K), cross-link locations were defined as the sites of deletions. Frequencies were given as fractions of the nucleotide in the normalized snoRNA with the highest deletion frequency.

Protein sequences for fluorescence polarization

Yellow: GST
Red: Halo
Green: SBP
Magenta: Gene of interest

hisGST-HALO-KHDRBS2
MSPIHHHHHHSPILGYWKIKGLVQPTRLLLEYLEEKYEEHLYERDEGDKWRNKKFELG
LEFPNLPYYIDGDVKLTQSMAIIRYIADKHNMLGGCPKERAEISMLEGAVLDIRYGVSRI
AYSKDFETLKVDFLSKLPEMLKMFEDRLCHKTYLNGDHVTHPDFMLYDALDVVLYMDP
MCLDAFPKLVCFKKRIEAIPQIDKYLKSSKYIAWPLQGWQATFGGGDHPPKSDLEVLFQ
GPLGLLEMAEIGTGFPFDPHYVEVLGERMHYVDVGPRDGTPVLFLHGNPTSSYVWRN
IIPHVAPTHRCIAPDLIGMGKSDKPDLGYFFDDHVRFMDAFIEALGLEEVVLVIHDWGSA
LGFHWAKRNPERVKGIAFMEFIRPIPTWDEWPEFARETFQAFRTTDVGRKLIIDQNVFI
EGTLPMGVVRPLTEVEMDHYREPFLNPVDREPLWRFPNELPIAGEPANIVALVEEYMD
WLHQSPVPKLLFWGTPGVLIPPAEAARLAKSLPNCKAVDIGPGLNLLQEDNPDLIGSEI
ARWLSTLEISGGSMEEEKYLPELMAEKDSLDPSFVHASRLLAEEIEKFQGSDGKKEDE
EKKYLDVISNKNIKLSERVLIPVKQYPKFNFVGKLLGPRGNSLKRLQEETGAKMSILGKG
SMRDKAKEEELRKSGEAKYAHLSDELHVLIEVFAPPGEAYSRMSHALEEIKKFLVPDYN
DEIRQEQLRELSYLNGSEDSGRGRGIRGRGIRIAPT*

hisGST-A1CF
MSPIHHHHHHSPILGYWKIKGLVQPTRLLLEYLEEKYEEHLYERDEGDKWRNKKFELG
LEFPNLPYYIDGDVKLTQSMAIIRYIADKHNMLGGCPKERAEISMLEGAVLDIRYGVSRI
AYSKDFETLKVDFLSKLPEMLKMFEDRLCHKTYLNGDHVTHPDFMLYDALDVVLYMDP
MCLDAFPKLVCFKKRIEAIPQIDKYLKSSKYIAWPLQGWQATFGGGDHPPKSDLEVLFQ
GPLGLLEMESNHKSGDGLSGTQKEAALRALVQRTGYSLVQENGQRKYGGPPPGWDA
APPERGCEIFIGKLPRDLFEDELIPLCEKIGKIYEMRMMMDFNGNNRGYAFVTFSNKVE
AKNAIKQLNNYEIRNGRLLGVCASVDNCRLFVGGIPKTKKREEILSEMKKVTEGVVDVIV
YPSAADKTKNRGFAFVEYESHRAAAMARRKLLPGRIQLWGHGIAVDWAEPEVEVDED
TMSSVKILYVRNLMLSTSEEMIEKEFNNIKPGAVERVKKIRDYAFVHFSNREDAVEAMK
ALNGKVLDGSPIEVTLAKPVDKDSYVRYTRGTGGRGTMLQG*

Cut PCBP1
GPLGLLEMDAGVTESGLNVTLTIRLLMHGKEVGSIIGKKGESVKRIREESGARINISEGN
CPERIITLTGPTNAIFKAFAMIIDKLEEDINSSMTNSTAASRPPVTLRLVVPATQCGSLIGK
GGCKIKEIRESTGAQVQVAGDMLPNSTERAITIAGVPQSVTECVKQICLVMLETLSQSP

QGRVMTIPYQPMPASSPVICAGGQDRCSDAAGYPHATHDLEGPPLDAYSIQGQHTISP
LDLAKLNQVARQQSHFAMMHGGTGFAGIDSSSPEVKGYWASLDASTQTTHELTIPNN
LIGCIIGRQGANINEIRQMSGAQIKIANPVEGSSGRQVTITGSAASISLAQYLINARLSSEK
GMGCS*

Cut mutPCBP1
GPLGLLEMDAGVTESGLNVTLTIRLLMHGKEVGSIIGKKGESVKRIREESGARINISEGN
CPERIITLTGPTNAIFKAFAMIIDKLEEDINSSMTNSTAASRPPVTPRLVVPATQCGSLIG
KGGCKIKEIRESTGAQVQVAGDMLPNSTERAITIAGVPQSVTECVKQICLVMLETLSQS
PQGRVMTIPYQPMPASSPVICAGGQDRCSDAAGYPHATHDLEGPPLDAYSIQGQHTIS
PLDLAKLNQVARQQSHFAMMHGGTGFAGIDSSSPEVKGYWASLDASTQTTHELTIPN
NLIGCIIGRQGANINEIRQMSGAQIKIANPVEGSSGRQVTITGSAASISLAQYLINARLSSE
KGMGCS*

Supplementary figure 1

RBPs identified by phase separation methods and recurrent RBP missense mutations

a

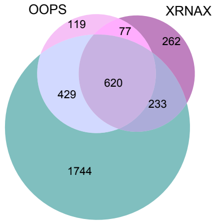

b

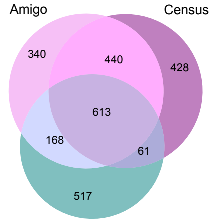

c

Recurrent missense muations in putative RBPs identified only by phase separation methods

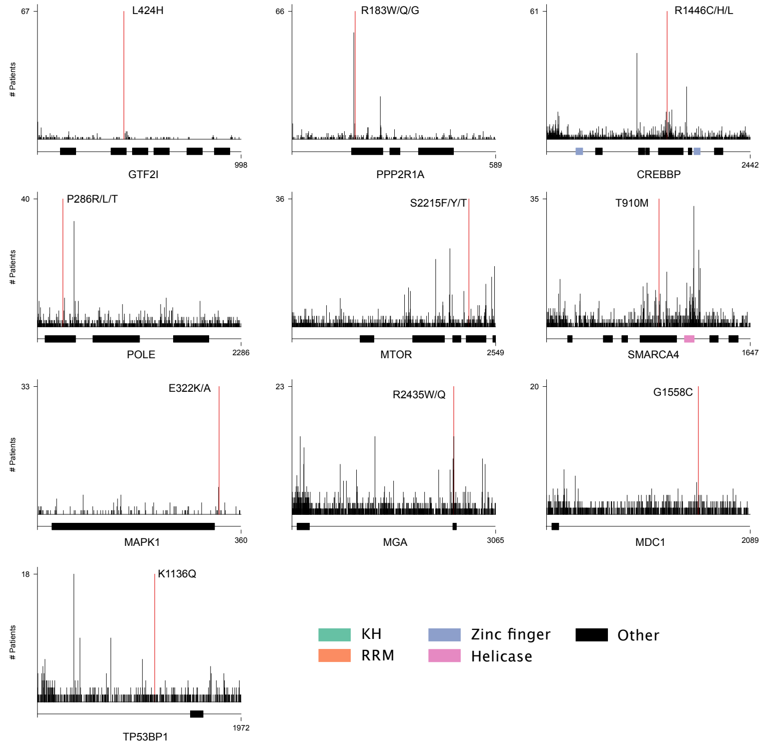

d

	Has RBD?	Direct RBP?	Known RBP?	Function	Cancers with missense mutation
GTF2I	None	Unknown	Potential	General transcription factor	Thymoma/Others
PPP2R1A	None	Unknown	Potential	Protein phosphatase 2A scaffold	ACC/Bowel/Uterine
POLE	None	Unknown	Potential	DNA polymerase catalytic subunit	Uterine/Bowel/Others
MTOR	None	Unknown	Potential	Kinase	Bowel/Uterine/Lung
MAPK1	None	Unknown	Potential	Kinase	HNSCC/Cervical/Others
MGA	None	Unknown	Potential	Transcription factor	Prostate/Bowel/Angiosarcoma
MDC1	None	Unknown	Potential	DNA damage checkpoint	Bladder/Prostate/Others
TP53BP1	None	Unknown	Potential	Double-strand break repair	Leukemia/Others
SMARCA4	Helicase	Unknown	Potential	Transcription activation	Brain/Pancreas/Others
CREBBP	Other	Unknown	Potential	Histone acetyltransferase	Lymphoma/Lung/ACC

e

Recurrent missense muations from the GENIE 8.0 database

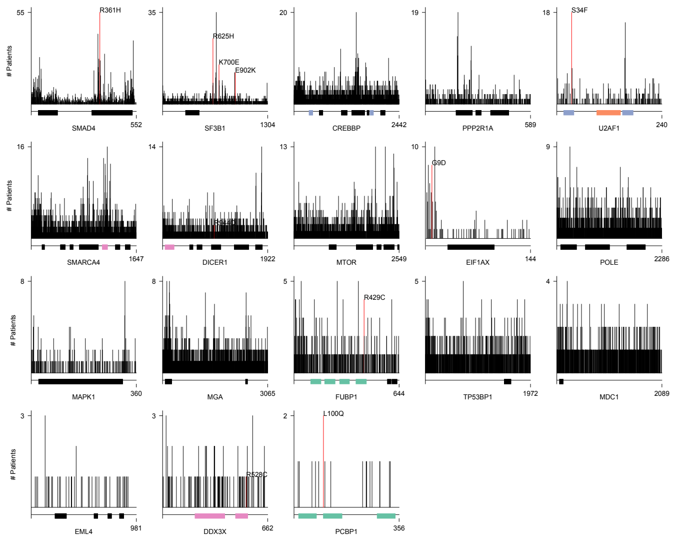

Supplementary figure 1. Additional analysis of recurrent missense mutations in known and putative RBPs. **a)** Comparison of RBPs identified by three different methods using phase separation followed by mass spectrometry. Phase separation methods are agnostic to whether the RNA is poly(A) tails. For OOPS and XRNAX, in which three cell lines were used, RBPs were subset to those identified in at least two of the three cell lines. **b)** Comparison of RBPs identified by phase separation methods - those identified in at least two of the three methods OOPS, XRNAX and PTex - with the RBP census and GO terms. **c)** The most frequent recurrent missense mutations in cancer in RBPs identified by phase separation methods (and not by the AMIGO and census databases). **d)** Features of RBPs identified by phase separation methods with the most frequent missense mutations in cancer. ACC: Adenoid Cystic Carcinoma, HNSCC: Head and Neck Squamous Cell Carcinoma. **e)** Recurrent missense mutations in RBPs and putative RBPs in GENIE data. Most GENIE sequencing was targeted at high-priority cancer-associated genes, resulting in many fewer recurrent missense for RBPs compared to TCGA data. SMAD4, SF3B1, U2AF1, EIF1AX identify the same mutation as the TCGA data as the highest, or one of the highest frequency mutations. Only a handful of PCBP1 missense mutations were identified in GENIE data, but L100 mutations were the only recurrent mutations observed (2 patients). The TCGA recurrent mutations in DICER1, FUBP1 and DDX3X were also observed in GENIE data, but only FUBP1 R429C was recurrent (4 patients) and was not the most prominent FUBP1 missense mutation.

Supplementary figure 2

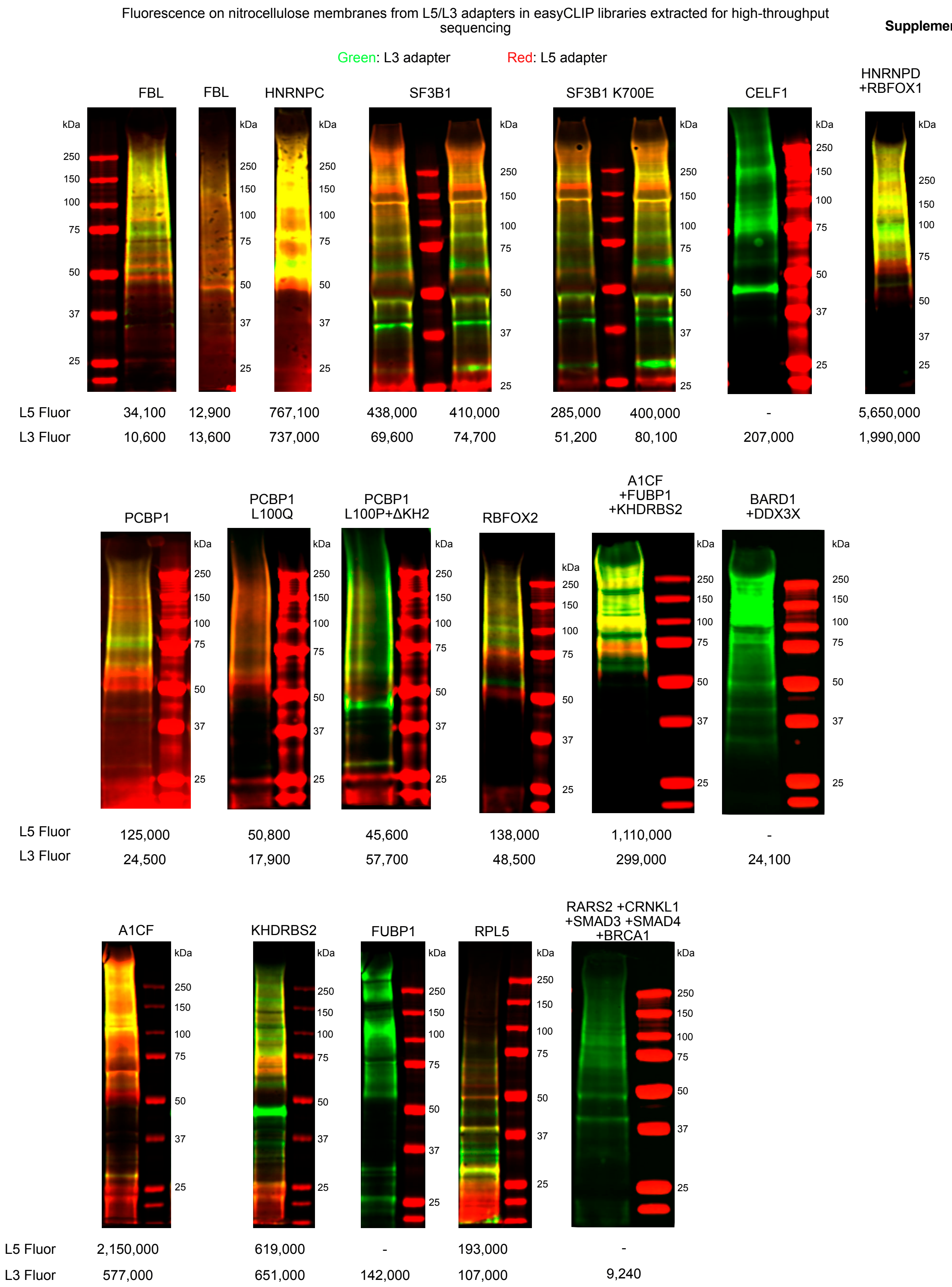

Supplementary figure 2. RNA fluorescence on nitrocelluose membranes after the purification of the indicated HA-tagged RBP or putative RBP while preparing easyCLIP libraries for sequencing. Red represents L5 adapter fluorescence (or protein ladder), and green represents L3 adapter fluorescence. In some cases a non-fluorescent L5 adapter was used, resulting in no L5 fluorescence. Images include all lanes excised for sequencing the indicated proteins except: HNRNPC, A1CF, KHDRBS2 and FUBP1 had 3 lanes, PCBP1, PCBP1 L100Q, PCBP1 ΔKH2, and RPL5 had 2 lanes (images are representative). Sample barcoding allowed multiple samples to be combined in one lane.

a

Method	Fraction of RT input to PCR	PCR cycles	fmols (σ)	fmols, extrapolated to 100% input to PCR (σ)	Fold increase	Fraction mappable	Minimum fold increase in mappable reads	Stated input library size, stated PCR efficiency	Input library size at 86% PCR efficiency	Input library size at 96% PCR efficiency
easyCLIP	16%	16	1,971 (241)	12,800 (1,445)	178	16%	30	NA	1,30,000,000 (2,100 amol)	226,000,000 (370 amol)
eCLIP	100%	16	72 (8)	72 (8)	1	Unknown, gel extracted	1	7,000,000 (12 amol), 86%	7,000,000 (12 amol)	NA

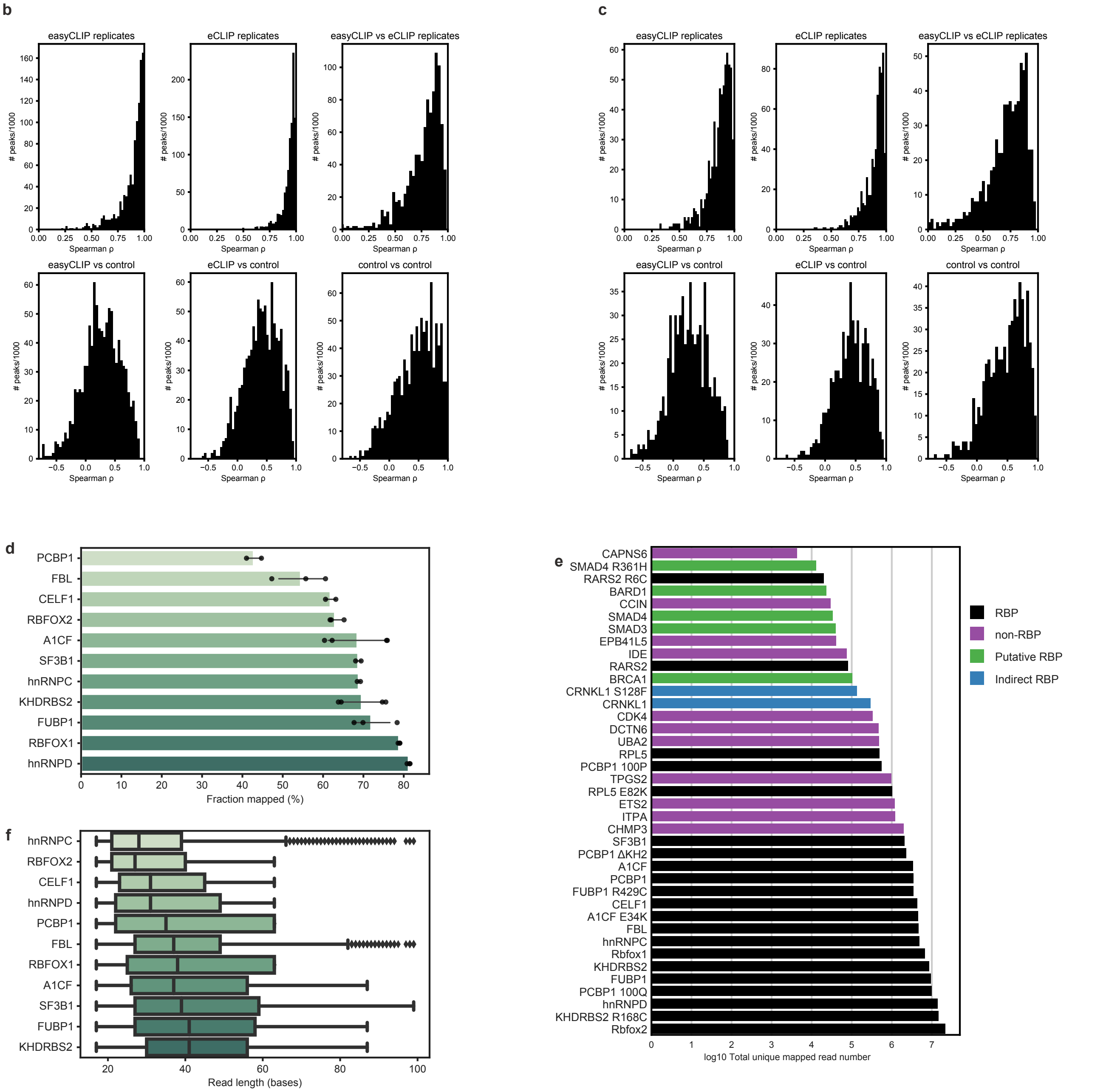

Supplementary figure 3. Comparison of easyCLIP to eCLIP. **a)** The comparison used the same amount of the same anti-RBFOX2 antibody, the same cell line, and the same number of cells to perform easyCLIP on RBFOX2. eCLIP produced 72 fmols of library after 16 PCR cycles per replicate, as reported[1]; easyCLIP produced ~13,000 fmols of library after the same number of cycles per replicate (n=3, extrapolating from PCR amplification of 16% of RT reactions). E.L. Van Nostrand *et al.* note that at 100% PCR efficiency their largest replicate would reach 100 fmol after 13 PCR cycles[1]. Dividing 100 fmol by 2^{13} gives an initial library size of 12 amol for eCLIP (7 million molecules) and a PCR efficiency of 86%. The subsequent information on RBFOX2 mapping in E.L. Van Nostrand *et al.*[1] may not have come from this benchmark sample, as the authors report 85% unique reads at 20 million reads sequencing depth, which appears impossible with a starting library of 7 million. eCLIP performed a size selection on the amplified library before sequencing, so the fraction of the input 12 amol that was usable is unknown. This easyCLIP sample did not undergo size selection before sequencing, resulting in many inserts too small to map, but 16% of reads were mappable. If easyCLIP PCR was 96% efficient (*vs* 86% for eCLIP), the starting pool would still be 370 amols. RBFOX2 data was obtained without substantial optimization (three RNAse concentrations were tried) – suggesting RBFOX2 does not represent an optimal case but a typical case. **b)** Spearman correlations of read density within 1000 nt of an RBFOX2 eCLIP peak for easyCLIP RBFOX2, eCLIP RBFOX2, and eCLIP input controls. **c)** Same as panel B, but for a random 1000 peak subset of easyCLIP RBFOX2 peaks, limiting to one easyCLIP peak per gene, with peaks defined relative to randomly chosen non-RBPs. **d)** The fraction of reads mapping to the genome for each set of CLIP-seq replicates, after short inserts were removed (A1CF and KHDRBS2 n=4, PCBP1, CELF1, SF3B1 and HNRNPC n=2, others n=3). Data, mean ± s.d. **e)** Unique mapped reads. All data was obtained from 293T cells except PCBP1 was obtained from the colon cancer cell line HCT116. Cellular inputs ranged from below 10 million cells (hnRNP C, exact number not recorded), to 10 million (one RBFOX2 replicate), to 20 million (two RBFOX2 replicates), to a maximum of a 15 cm plate. RBFOX2, FBL, and hnRNP C libraries were obtained from antibodies to the endogenous proteins, the others were obtained from FLAG tag purifications from either constructs either integrated at the AAVS1 locus (PCBP1) or transiently over-expressed from a vector (the others). **f)** The average read length for the indicated datasets (n=10,000 reads randomly selected from fastq). HNRNPC and RBFOX2 libraries were digested more than would have been optimal. Boxplots show quartiles, center line shows the median and whiskers show the maxima and minima except if a value is beyond 1.5 times the interquartile range, it is plotted individually.

Supplementary figure 4

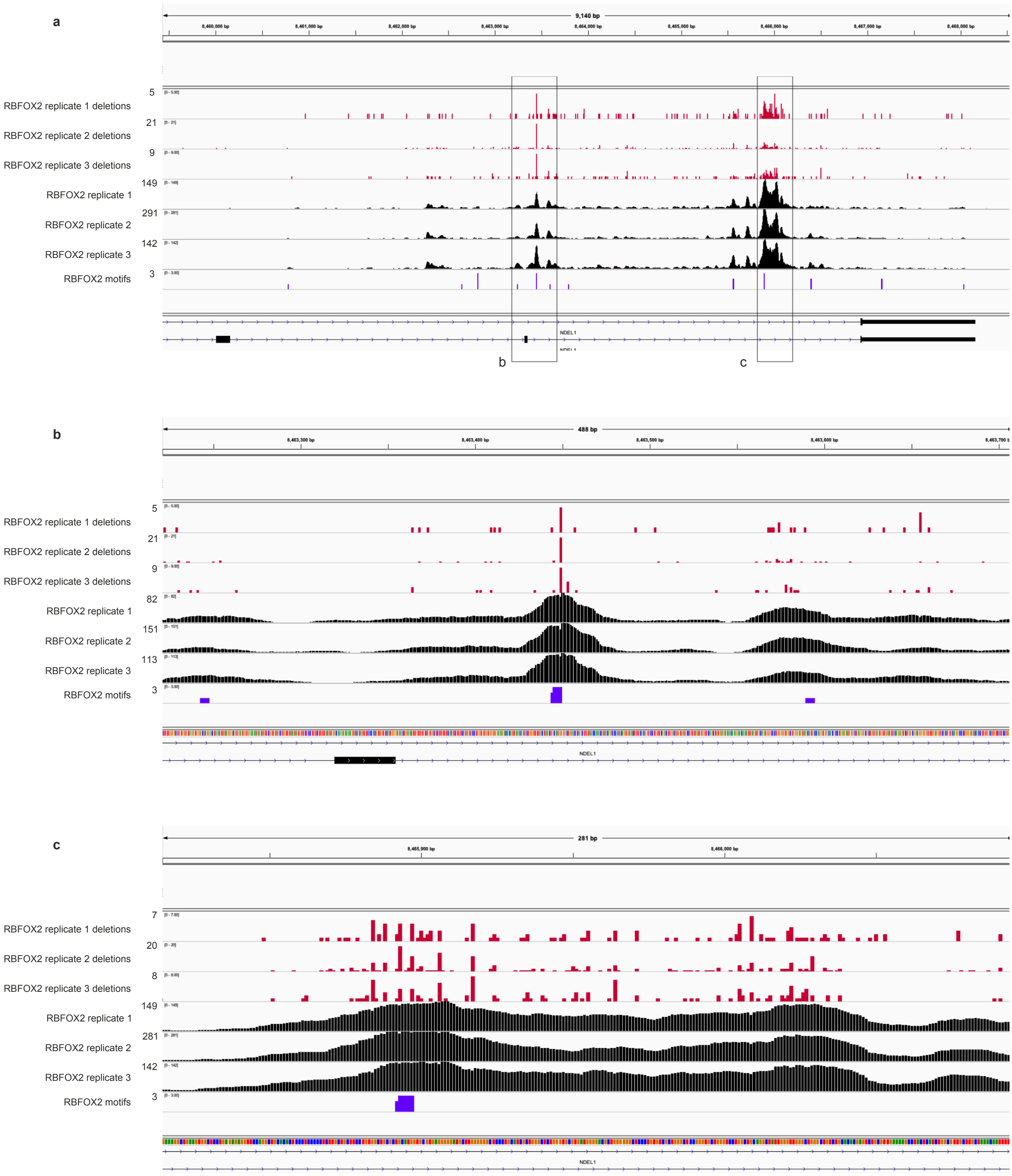

Supplementary figure 4. Comparison of easyCLIP to eCLIP and reproducibility of cross-link-induced deletion positions. Snapshot of the IGV browser viewing easyCLIP RBFOX2 reads at the same NDEL1 locus as shown in E.L. Van Nostrand *et al.*[1] Figure 1D, showing identification of the same binding sites. The middle and bottom panels are closer views of two regions shown in the top panel. Note that the scale bar in E.L. Van Nostrand *et al.* is reads per million; the scale here is simply raw reads. The position of deletions in reads are in red (top tracks), read density is plotted in black (middle tracks), and the RBFOX2 motifs tract in purple (bottom) shows the location of GCAUG motifs (the Rbfox binding site) on the plus strand, with a value of one placed on GCAUG, a value of two placed on UGCAUG (a preferred form of the motif), and allowing values to sum. **a)** View in IGV of RBFOX2 binding near an alternatively spliced exon in *NDEL1* and surrounding introns. Regions zoomed-in for panels B and C are indicated. **b)** Closer zoom-in of the alternatively spliced exon. **c)** Zoom-in of a region of high RBFOX2 binding in the intron downstream of the alternatively spliced exon.

Supplementary figure 5

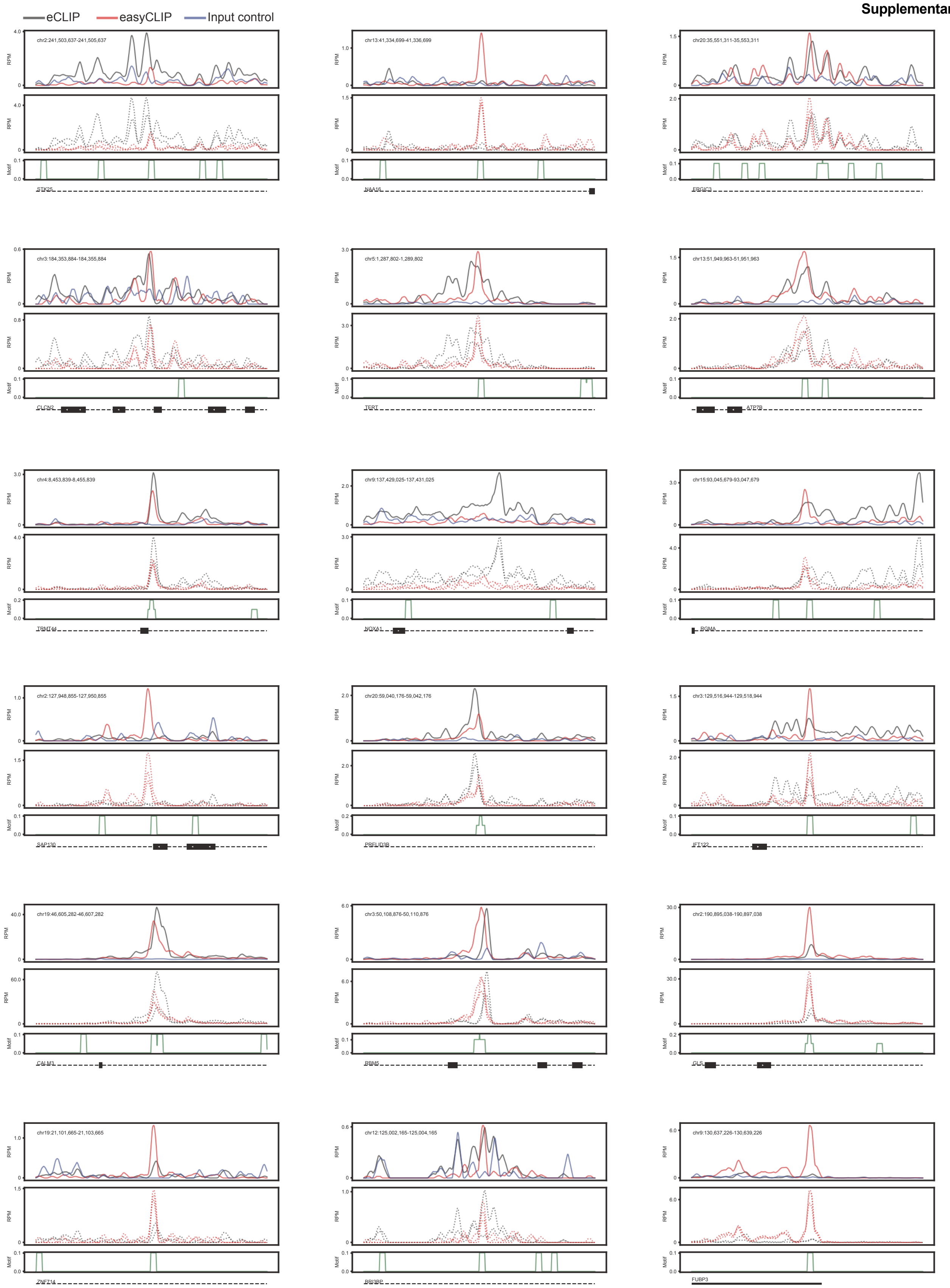

Supplementary figure 5. Genomic regions at randomly selected easyCLIP RBFOX2 peaks show easyCLIP replicates correlate with each-other and with RBFOX2 eCLIP, all done with the same antibody and in HEK293T cells. Four panels are shown for each genomic region. The top panel shows easyCLIP RBFOX2 reads per million in red, eCLIP in black, and the 293T input control for eCLIP in blue. Signal was smoothed with a 50 nt window. The second panel from top shows individual easyCLIP replicates in red, and individual eCLIP replicates in black. The third panel from top shows the occurrence of the RBFOX2 binding site (the shorter GCAUG form), with a 1 value placed at each instance, and then smoothed with a 50 nt window. The bottom panel denotes the local gene body, if one exists, with exons drawn in a thicker line. For clarity, only one gene and isoform is shown.

Supplementary figure 6

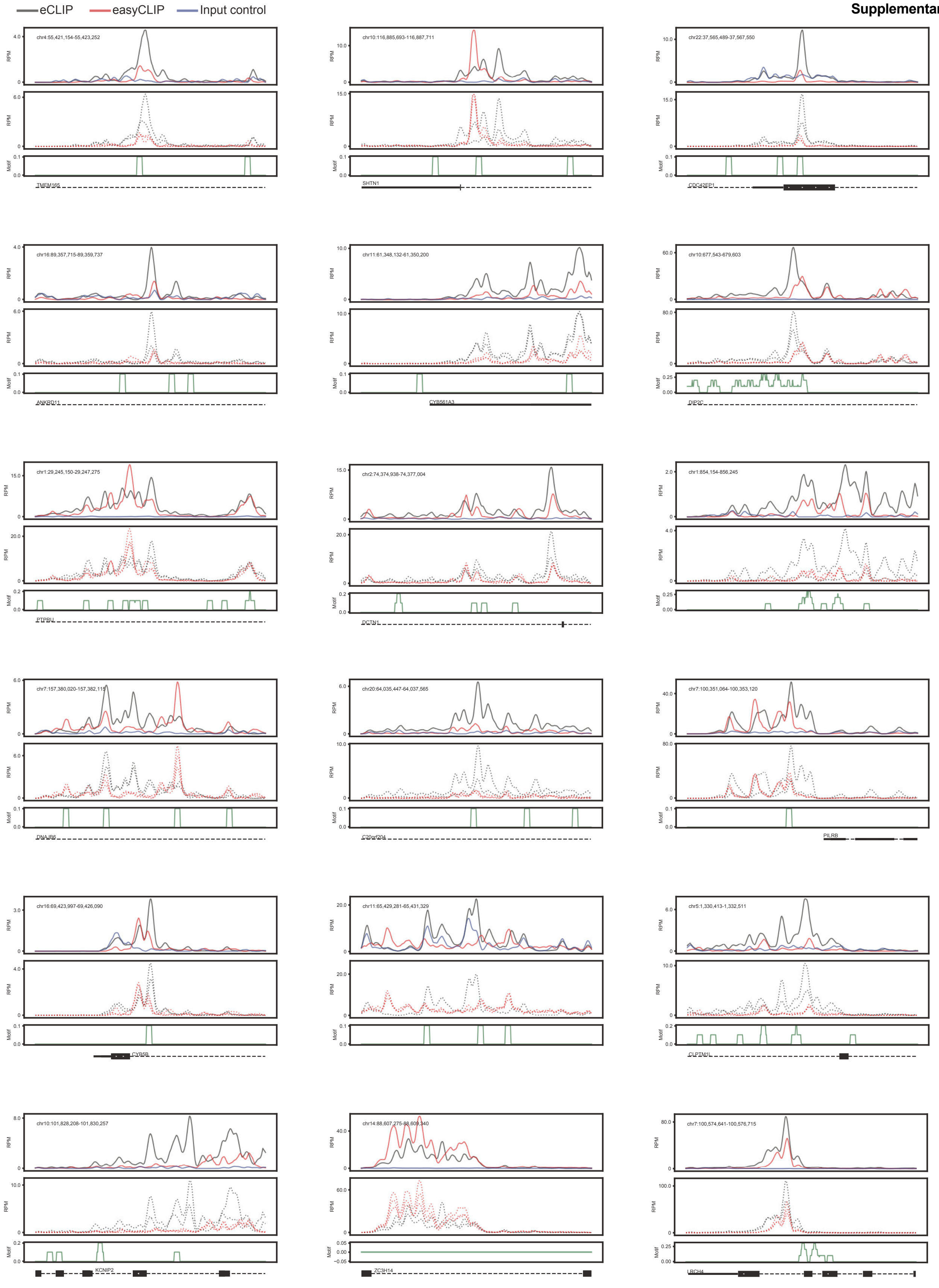

Supplementary figure 6. Genomic regions at randomly selected eCLIP RBFOX2 peaks show easyCLIP replicates correlate with each-other and with RBFOX2 eCLIP, all done with the same antibody and in HEK293T cells. This figure is the same as Fig S5, except random eCLIP peaks were used instead of random easyCLIP peaks.

Transient transfections resulted in low protein abundance **Supplementary figure 7**

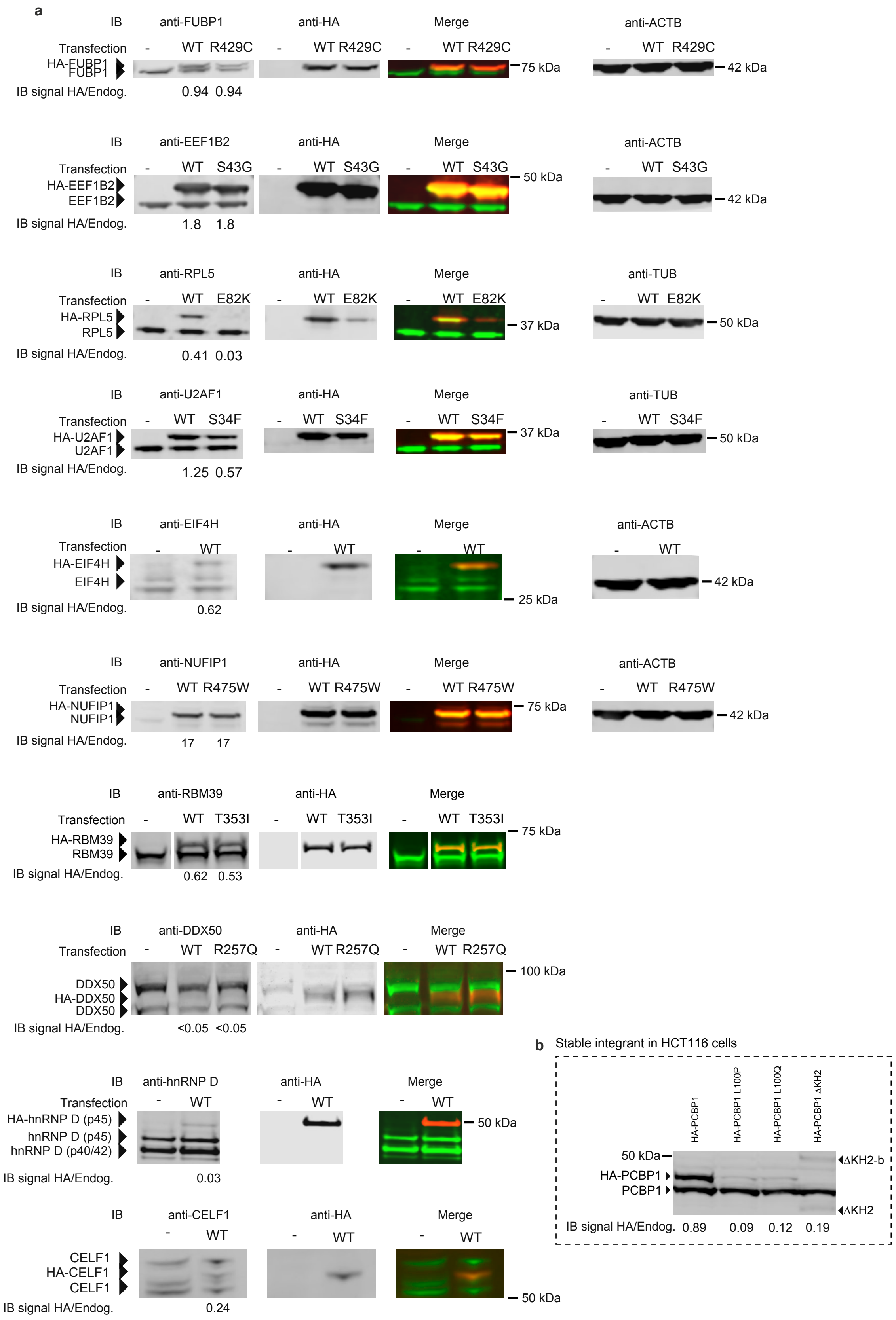

Supplementary figure 7. a) Immunoblots of the relative abundance of transiently expressed, HA-tagged RBPs vs endogenous expression of the same protein in HEK293T cells. The transient protein abundance was <2-fold of endogenous protein in 3/10 cases, within 2-fold in 6/10 cases, and >2-fold of endogenous in 1/10 cases tested. Only RBPs expected to be expressed in HEK293T cells were tested. The p40/42/45 in hnRNP D are known splicing isoforms of the indicated molecular weight; HA-hnRNP D is the p45 isoform. Experiments were performed once. **b)** PCBP1 WT and mutant forms were integrated into HCT116 cells using an AAVS1 safe harbor locus. ΔKH2 PCBP1 lacks the second KH domain, so it runs at a lower molecular weight, but a second form, possibly a dimer, also appears (ΔKH2-b). Experiment was performed twice. IB: immunoblot. Endog.: endogenous protein. HA: hemagglutinin tag.

Supplementary figure 8

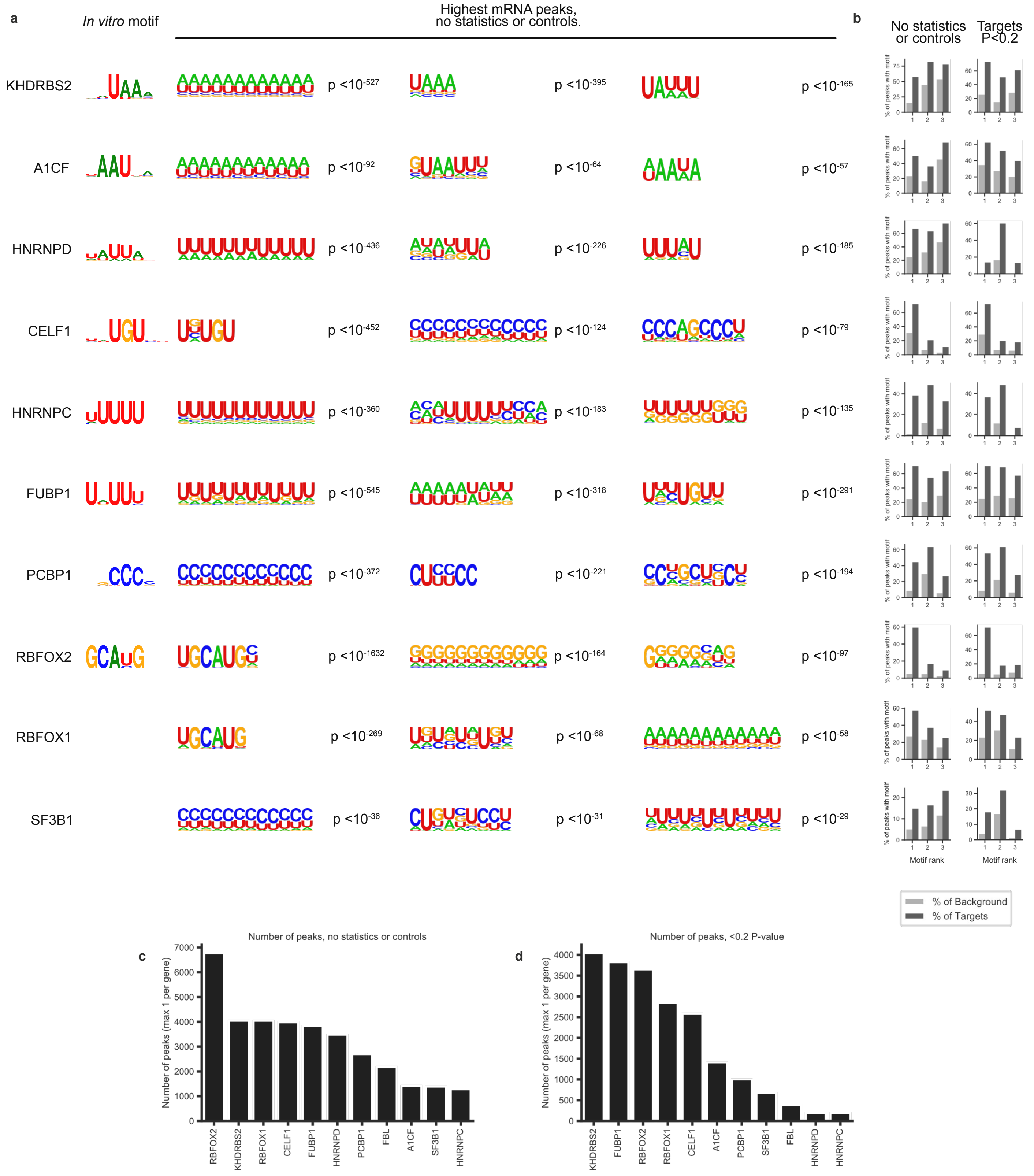

Supplementary figure 8. Comparison between easyCLIP motifs and *in vitro* enriched motifs from RNA Bind-n-Seq[1] shows good agreement between methods for all proteins. **a)** The top motif from RNA Bind-n-Seq is listed on the left, followed by the top three (by P-value enrichment over random sequences) motifs from easyCLIP. Motif enrichment significance computed by HOMER (hypergeometric test with p-value corrected for multiple testing). **b)** The percentage of peaks containing the indicated motif. Proteins are matched by row to panel **a**. The leftmost motif is Rank 1, *ect.* **c)** The number of peaks (maximum one per gene) without controls. **d)** As panel C, but using a P<0.2 cutoff *vs* controls (controls fit to negative binomial and Benjamini-Hochberg method for adjustment, see methods).

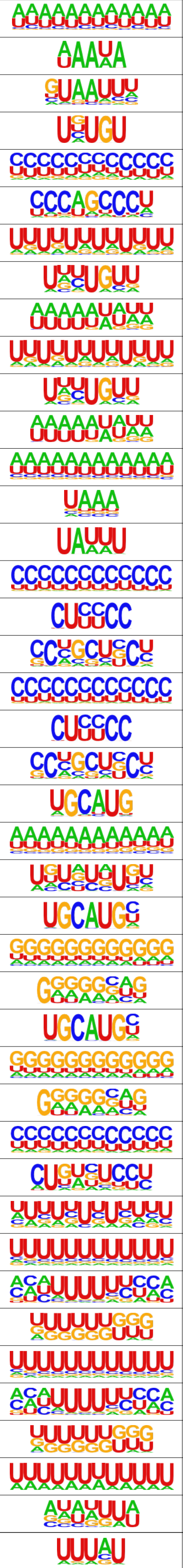

Dataset	Motif	Log10 p value	# Peaks	% targets	% control	Length (bp)
A1CF		-1728	2042	95.15%	34.28%	244
A1CF		-485	2042	98.97%	75.42%	244
A1CF		-876	2042	87.02%	42.65%	244
CELF1		-1686	5159	95.39%	61.12%	231
CELF1		-1396	5159	44.18%	13.86%	231
CELF1		-403	5159	26.28%	11.86%	231
FUBP1		-38629	35642	98.95%	31.66%	278
FUBP1		-17901	35642	97.50%	54.06%	278
FUBP1		-25490	35642	96.57%	41.65%	278
FUBP1 eCLIP		-2775	3955	80.20%	24.02%	200
FUBP1 eCLIP		-1144	3955	80.05%	42.98%	200
FUBP1 eCLIP		-2096	3955	82.15%	32.29%	200
KHDRBS2		-44218	26585	95.14%	14.31%	249
KHDRBS2		-9921	26585	99.38%	66.59%	249
KHDRBS2		-4755	26585	99.38%	81.28%	249
PCBP1		-2038	3335	44.92%	6.00%	226
PCBP1		-693	3335	72.80%	41.05%	226
PCBP1		-784	3335	38.65%	11.87%	226
PCBP1 eCLIP		-1378	2749	41.11%	6.26%	230
PCBP1 eCLIP		-322	2749	67.59%	43.69%	230
PCBP1 eCLIP		-947	2749	43.65%	11.02%	230
RBFOX1		-5532	30712	97.35%	76.22%	304
RBFOX1		-5351	30712	80.12%	51.74%	304
RBFOX1		-9042	30712	93.11%	59.55%	304
RBFOX2		-31032	38563	70.24%	14.27%	264
RBFOX2		-4885	38563	32.58%	13.05%	264
RBFOX2		-2178	38563	24.38%	12.16%	264
RBFOX2 eCLIP		-3294	15034	40.29%	13.52%	241
RBFOX2 eCLIP		-5760	15034	47.36%	11.91%	241
RBFOX2 eCLIP		-4910	15034	42.82%	11.08%	241
SF3B1		-157	1670	33.71%	16.17%	229
SF3B1		-114	1670	42.75%	25.93%	229
SF3B1		-694	1670	75.15%	30.90%	229
hnRNPC		-904	1030	34.56%	1.26%	231
hnRNPC		-541	1030	57.77%	13.88%	231
hnRNPC		-237	1030	41.75%	14.08%	231
hnRNPC eCLIP		-58657	49039	39.22%	0.87%	246
hnRNPC eCLIP		-25514	49039	58.40%	14.32%	246
hnRNPC eCLIP		-14843	49039	45.54%	13.47%	246
hnRNPD		-9404	8346	97.63%	28.35%	255
hnRNPD		-4394	8346	97.11%	51.97%	255
hnRNPD		-2074	8346	98.86%	74.20%	255

Supplementary figure 9. MACS2 peak calling of easyCLIP and eCLIP peaks shows good motif coverage, motif enrichment, and peak numbers. Length (bp) denotes the median peak length in base pairs. The presence of motifs and significance were calculated by HOMER (hypergeometric test).

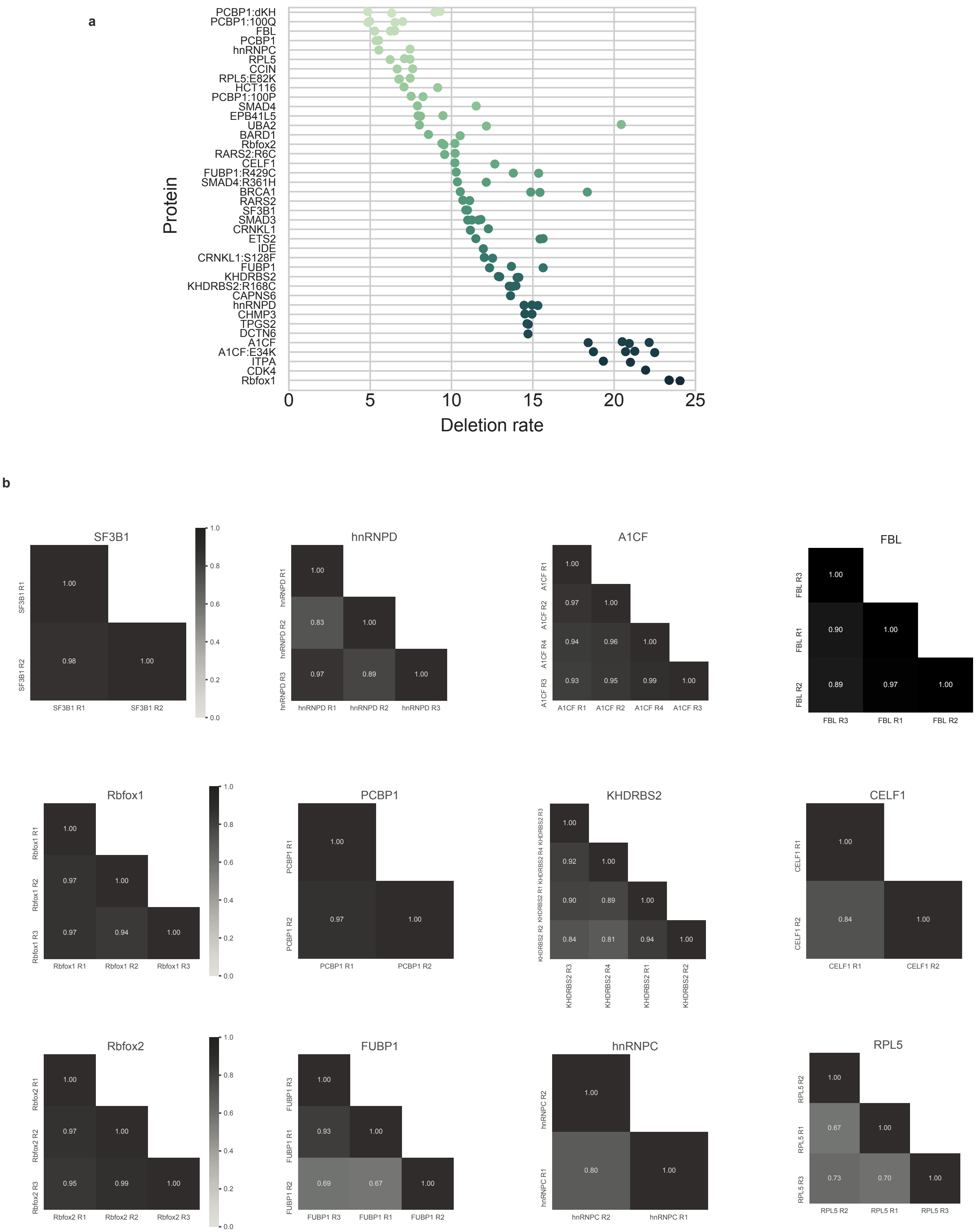

Supplementary figure 10. Deletion rates and reads-in-peak replicate correlations. **a)** The percentage of mapped reads with deletions. Previous work has found 8-20% of HITS-CLIP reads mapping to mRNA possessed a deletion, based on Nova and Ago. Our range of 5-25%, with a larger protein set, is consistent with this general frequency of cross-link-induced deletions. Note non-RBPs have a similar rate of deletions. **b)** Pearson correlations for reads in MACS2-called peaks. The color scale, from 0 to 1, is the same for all panels.

Supplementary figure 11

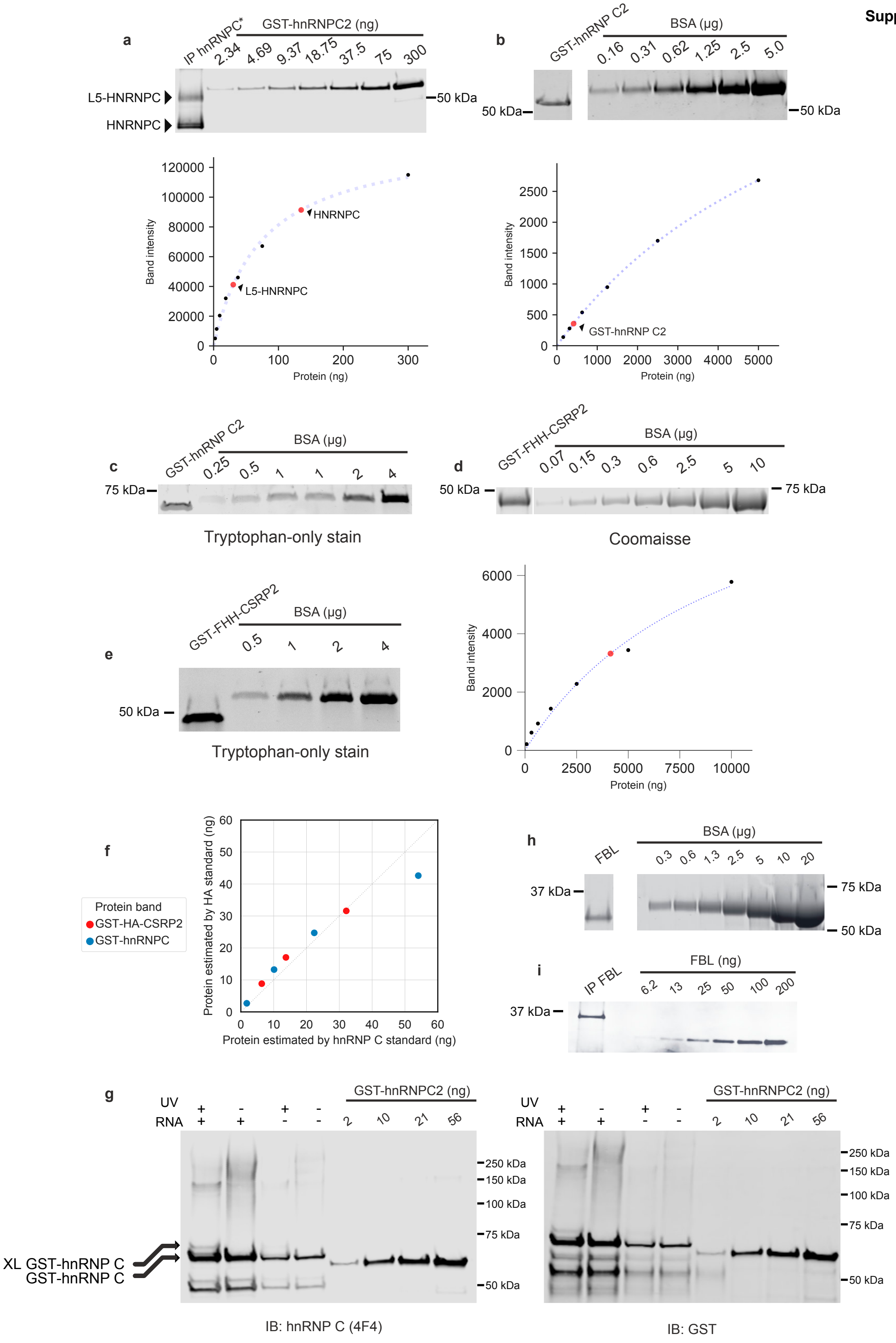

Supplementary figure 11. Quantification of purified recombinant protein and its application to absolute quantitation of immunopurified protein in CLIP. FHH: Flag-HA-His tag. IB: immunoblot. **a)** Quantification of immunopurified endogenous hnRNP C using a GST-hnRNP C standard. The gel is a western blot probed with antibodies to hnRNP C. Endogenous hnRNP C is smaller than GST-hnRNP C but is shown at the same vertical position in this panel as GST-hnRNP C for visualization. In the graph, black dots represent GST-hnRNP C standards, the blue line is a best fit hyperbolic curve, and the red dot is immunopurified endogenous hnRNP C. **b)** Quantification of purified GST-hnRNP C expressed in *E. coli*. GST-tagged hnRNP C was purified from *E. coli* using glutathione resin, and then run next to a standard curve of BSA protein on an SDS-PAGE gel. Gel was stained with Coomaisse and fluorescence measured at 700 nm. In the graph, black dots represent BSA standards, the dotted line is a fit hyperbolic curve, and the red dot represents the purified GST-hnRNP C, its position on the y-axis determined from the standard curve. The larger graph is focused on the lower quantities of GST-hnRNP C, while the larger graph is the same graph zoomed out to include all standards. **c)** Quantification of GST-hnRNP C using a tryptophan-reactive dye (Bio-Rad Stain-Free Gel). Gel was subsequently stained with Coomaisse to determine Coomaisse staining of GST-hnRNP C and BSA was not biased. **d)** Coomaisse quantification of purified, recombinant GST-FLAG-HA-His-CSRP2 (GST-FHH-CSRP2), the HA standard. CSRP2 was used in this construct because this fusion protein purifies in very high quantities. The hyperbolic curve fit is as in panel B. For panels a-d, experiments were performed at least twice. **e)** Quantification of GST-FHH-CSRP2 using a tryptophan reactive-dye to test for a bias in Coomaisse-staining of the HA standard. No bias was observed. **f)** Comparison of the quantification standards for HA and hnRNP C. Dilutions of each standard were run on the same gel and western blotted for GST. The standard curve of each protein stock was used to estimate the quantities of the other stock. The proximity of the dots to the 45° line indicate a good agreement. Experiment was performed once. **g)** The 4F4 anti-hnRNP C antibody shows little bias between cross-linked and non-cross-linked hnRNP C. Recombinant GST-hnRNP C (made in-house) was incubated with a poly(U)$_{10}$ RNA oligonucleotide (IDT) and UV cross-linked. The resulting mixture, along with GST-hnRNP C (Abnova) standards was run on a denaturing SDS-PAGE gel and transferred to a nitrocellulose membrane for immunoblotting against hnRNP C (4F4) or GST. No significant difference between anti-GST and anti-hnRNP C antibodies in the ratio of cross-linked to non-cross-linked hnRNP C was observed. Experiment was performed once. **h)** Coomaisse quantification of purified, recombinant FBL. Purified FBL protein (Prospec, enz-566) was comprised of FBL amino acids 83-321 with an added 23 amino acid tag added, and the FBL antibody (Bethyl, A303-891A) was made against an immunogen between amino acids 271-321 of FBL. As a result, the purified FBL runs faster than endogenous FBL, but both share the entire immunogen used for immunoblotting. Experiment was performed once. **i)** Immunoblot quantification of immunopurified FBL using the recombinant FBL visualized in panel H. Experiment was performed three times.

Supplementary figure 12

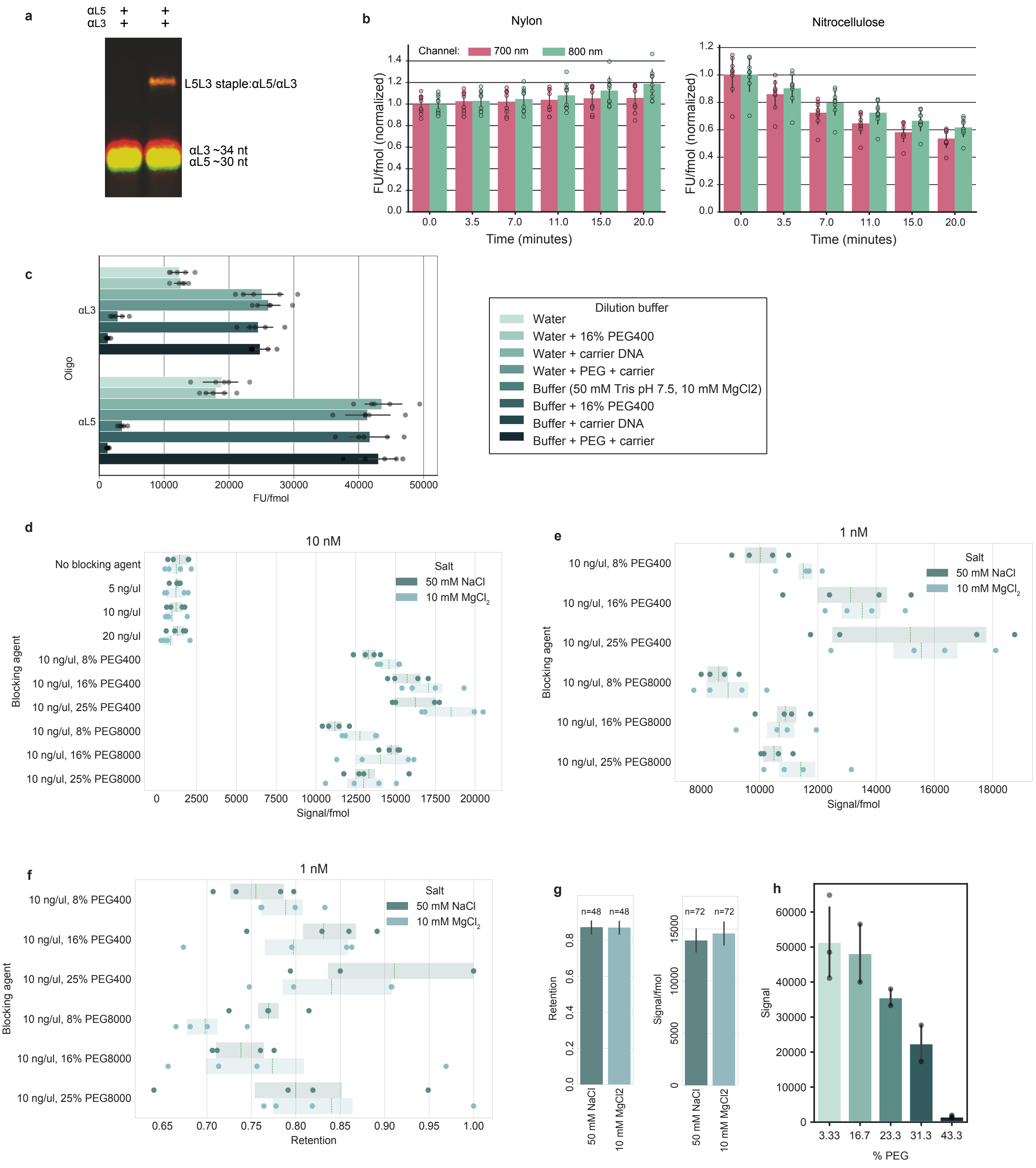

Supplementary figure 12. Developing a method to quantify low fmol amounts of adapter. **a)** A staple oligonucleotide may be used to shift the antisense oligonucleotides in Fig 3D in a single molecule to determine relative fluorescence and control both adapter quantifications to a single complex. Experiment was performed >3 times. **b)** Fluorescence on nylon and nitrocellulose for dot blots of αL3 and αL5 labelled respectively with IR680RD and IR800CW. Signal remains high on nylon, but decays on nitrocellulose**.** Data is mean ± s.d. for n=9 independent samples. **c)** The choice of dilution solution has a large effect on fluorescence. An equimolar mixture of αL3 and αL5 was dilute to 1 nM in the indicated solutions. 2 μL (2 fmols) of diluted oligonucleotide were then dot blotted on nylon and fluorescence measured on a Li-Cor scanner. Carrier DNA was an equimolar solution of 10, 15, and 35 nucleotide poly(A) oligonucleotides. Data is mean ± s.d. for n=5 independent samples. **d)** Fluorescence per fmol of αL3 oligonucleotide after diluting to 10 nM in 50 mM Tris pH 7.5 with the indicated salts and blocking agents. Carrier DNA was an equimolar solution of 10, 15, and 35 nucleotide poly(A) oligonucleotides at the indicated ng/μL concentrations. All PEG solutions had 10 ng/μL carrier DNA. Carrier DNA is not sufficient to block signal loss upon dilution. Both monovalent and divalent salts had similar effects. PEG400 and PEG8000 both preserved signal, and higher concentrations generally worked better. For D-F, boxplots represent the interquartile range and the central bar the mean for n=4 independent samples. **e)** The 10 nM solution in panel B was diluted to 1 nM. PEG400 leads to slightly higher fluorescence than PEG8000. Solutions lacking PEG are not depicted due to low signal to noise ratios. **f)** Retention of signal during a 10-fold dilution. Retention is the fluorescence per fmol of the 1 nM solution divided by the fluorescence per fmol of the 10 nM. The choice of salt has no consistent effect. Higher PEG concentrations are better blocking agents. PEG400 and PEG8000 have a similar performance as blocking agents. **g)** The choice of 50 mM NaCl or 10 mM $MgCl_2$ has no effect on oligonucleotide loss during dilution (retention) or on signal per fmol. Data is the mean ± 95% confidence interval for n=48 (left) or n=72 (right) samples. Retention samples are 24 samples serially diluted twice for 48 measurements. Using only one dilution for either panel does not affect the conclusion. **h)** It is safe to run DNA duplexes on 20% polyacrylamide TBE gels (NuPAGE, 12 well, ThermoFisher) at 16.7% PEG400, but higher concentrations lead to fluorescence loss in the duplex, probably due to unfolding of the DNA duplex. Data is mean ± s.d for n=3 (3.3% and 43.3 %PEG) or n=2 (others) across two experiments.

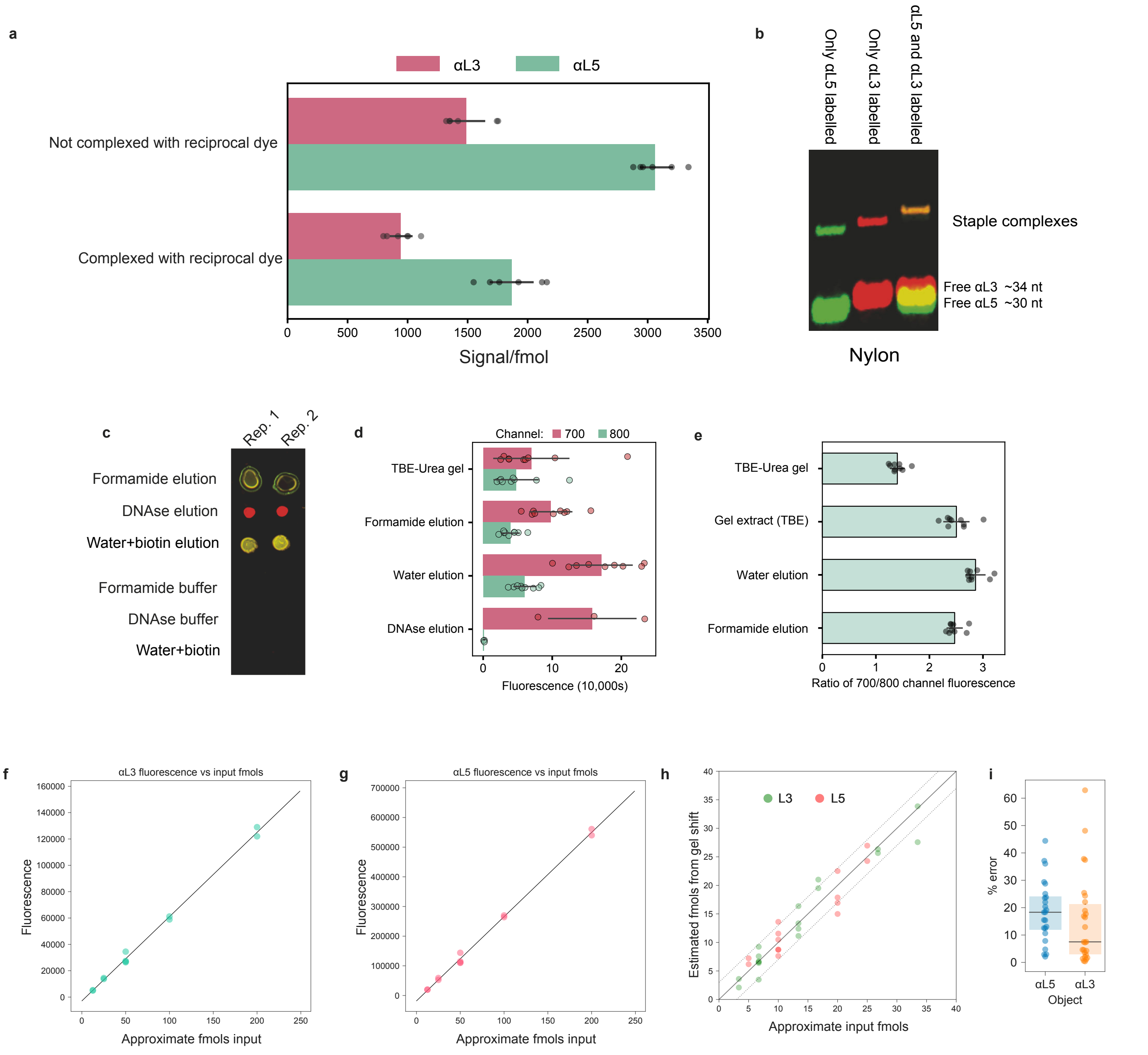

Supplementary figure 13. Signal interference between IR800CW and IR680RD dyes (A-B), performance of streptavidin elution methods (C-E), and the model-fitting and testing of an anti-sense oligonucleotide shift method of adapter concentration (F-I). **a)** The IR800CW and IR680RD dyes decrease in fluorescence when tethered to the same complex. An excess of αL5 and αL3 were mixed with 50 fmol of an oligonucleotide bearing one copy each of the L5 and L3 sequences, termed the staple oligonucleotide. αL5 was paired with either labelled or unlabeled αL3 to determine the effect of tethering αL3 near αL5, and the reciprocal case was applied to αL3. Complexes were run on a TBE gel in TBEN buffer (0.5X TBE plus 50 mM NaCl) and transferred to a nylon membrane for quantification. Data is the mean ± 95% confidence interval from n=6 independent samples over 2 experiments. **b)** Labelled complexes always traveled higher on the gel (right panel). Each dye shifts ~6 nucleotides higher on a TBE gel. Experiment was repeated twice. **c)** L5 and L3 adapters were ligated together *in* vitro, run on a TBE-urea gel, gel extracted, purified using streptavidin beads (MyOne C1, ThermoFisher), and then eluted by the indicated method. This image shows an example of eluates dot blotted on nitrocellulose. Note the peculiar shape of formamide dots. No fluorescence is observed in buffer alone. Water+biotin elution used 100 nM biotin. Formamide elution was 95% formamide with 10 mM EDTA (as suggested by ThermoFisher, who state elution is >95% by this method). DNAse elution used an excess of DNAse I (Ambion) in the buffer supplied by the manufacturer. **d)** Fluorescence quantification of the same linker-linker dimers depicted in panel A after each elution method. "TBE-urea gel" indicates fluorescence in the TBE-urea gel before extraction and streptavidin purification. Heating in water with 100 µM biotin was effectively complete, as it yielded similar L5 (700 nm) fluorescence as DNAse elution, which is likely to be complete, and similar fluorescence overall as formamide elution, which is complete according to the manufacturer (ThermoFisher). Data, mean ± s.d. for n=9 independent samples, except DNAse I n=3. **e)** Water, formamide and TBE-urea gels all affect relative L5/L3 fluorescence (IR680RD/IR800CW). The ratio of dye molecules is 1:1 in all cases, as all cases represent linker-linker dimers. Data, mean ± s.d. for n=9 independent samples. **f)** Fluorescence of the αL5 oligonucleotide in the staple-αL5-αL3 complex as a function of staple oligonucleotide quantity. Signal fits to a linear model (solid line). **g)** Fluorescence of the αL3 oligonucleotide in the same complexes as A. Signal is again highly linear (solid line is a linear fit). **h)** Known concentrations of L5 and L3 adapters and staple oligonucleotide were shifted by αL5 and αL3 and a fit to a linear model. As with staple oligonucleotides, data is linear: the solid line represents a perfect fit, dashed lines represent + or – 3 fmols. **i)** Error in the estimates made in panel C. The method is reasonably accurate, with average errors around 20%. The parameters (slope and intercept) from panel C were then used to estimate oligonucleotide concentrations for ligation efficiency determinations, after applying a scaling factor based on the fluorescence of αL5/ αL3 oligonucleotides in 50 fmol staple complexes. The calculation is described in github.com/dfporter/easyCLIP/doc/ in the README_fluorescence.md file.

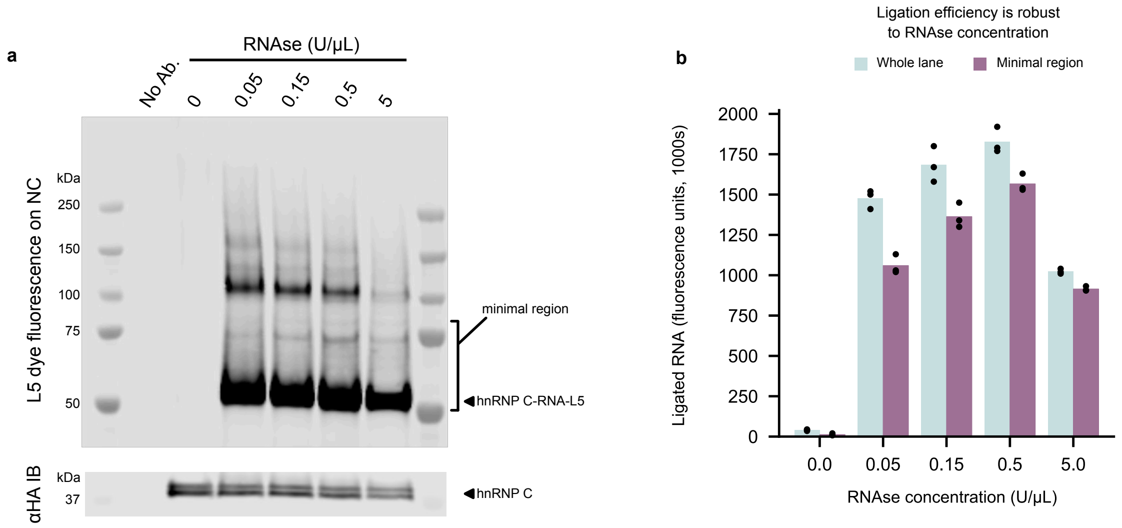

Supplementary figure 14. The effeect of RNAse concentration on ligation eficiency. **a)** Visualization of ligated RNA and purified protein for a gradient of 0.05-5 U/µL RNAse ONE. **b)** Quantification of the L5 fluorescence signal in panel A, which represents successful ligations, without dividing by the amount of purified, un-cross-linked protein (n=3). Dividing by the amount of purified protein (as in Fig 4H) is very similar; in both cases, there is less than a 2-fold change in ligation efficiency between any two concentrations. Bars represent the mean.

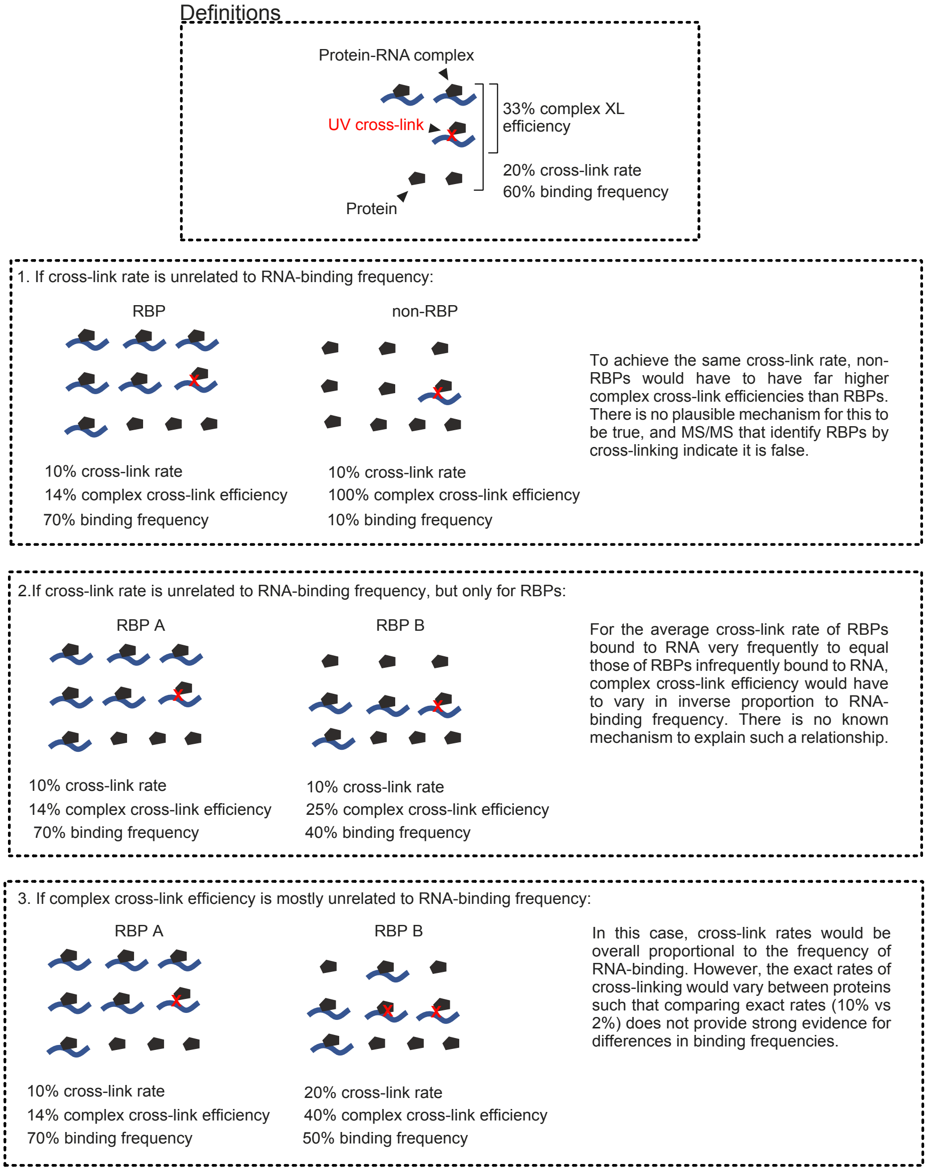

Supplementary figure 15. Definition of terms and theoretical basis for study. Three possible statements are given and illustrated; we suggest only the third is likely. Cross-link rate is the binding frequency multiplied by the complex cross-link efficiency. We argue that cross-link rate is proportional to binding frequency as long as the complex cross-link rate is not exactly inversely related to binding frequency. There is no known mechanism to support the possibility of complex cross-link efficiency being inversely proportional to binding frequency, and we suggest it is implausible. As a result, cross-link rates are proportional to binding frequency overall, but the largely unknown nature of complex cross-link efficiencies suggests that exact levels of cross-linking are probably not to be taken as proportionally exact measures of binding frequency.

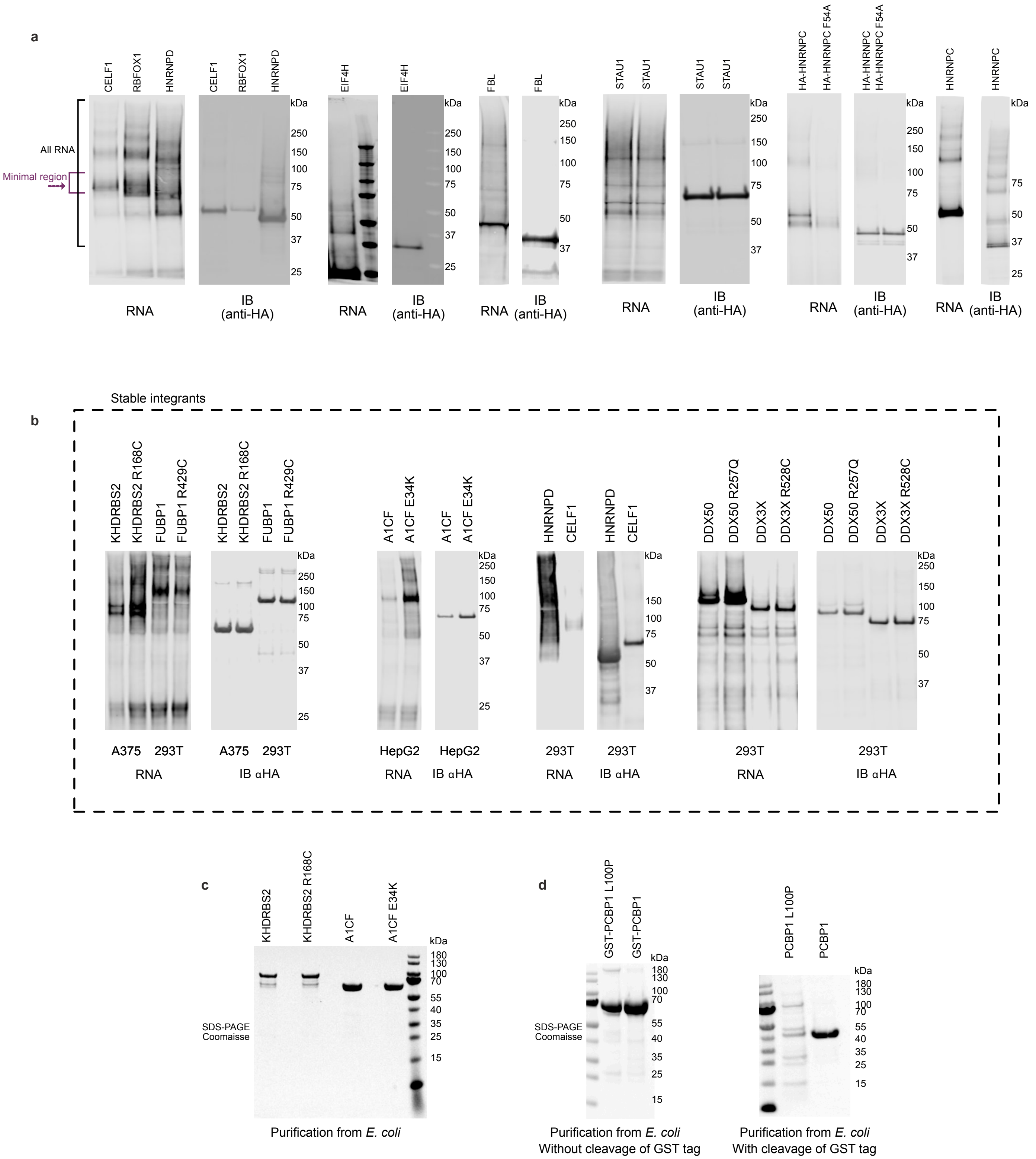

Supplementary figure 16. a) RNA fluorescence (L5 adapter) and immunoblot signal (anti-HA) for nitrocellulose membranes used to calculate cross-link efficiencies for the indicated proteins. Experiments repeated 2 times (RBFOX1 and STAU1) or 3 times (CELF1, HNRNPD, EIF4H, FBL, HNRNPC and HA-HNRNPC). **b)** RNA fluorescence (L5 adapter) and immunoblot signal (anti-HA) for nitrocellulose membranes used to calculate cross-link efficiencies for the indicated proteins. Experiments repeated 3 times except DDX50 repeated 2 times. **c)** Purified proteins expressed in *E. coli* used for fluorescence polarization. Experiment performed twice. **d)** Purified PCBP1 WT/L100P proteins expressed in *E. coli* used for fluorescence polarization with (*left*) and without (*right*) cleavage of the GST tag. After cutting, only a small amount of PCBP1 L100P could be recovered, with most of the protein being contaminating bands. Experiment performed twice.

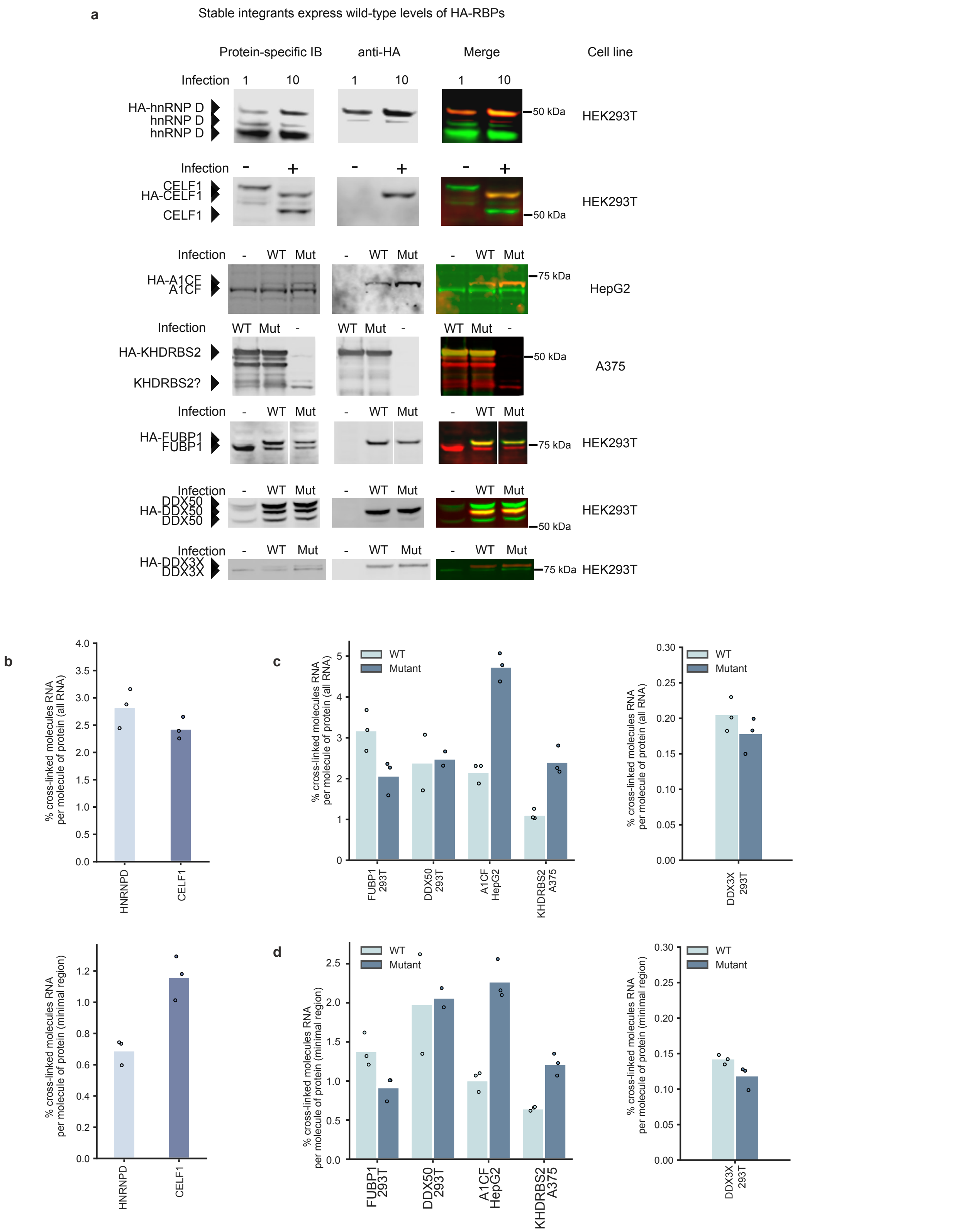

Supplementary figure 17. Viral integration of the indicated RBPs reproduces results from transient transfection in 293T cells. Experiment performed once. **a)** Stable integrants express protein levels similar to the endogenous protein. A375 cells are a melanoma line, which we used because the KHDRBS2 R168C mutation occurs mostly in melanoma. We could not validate that the endogenous KHDRBS2 band was actually KHDRBS2. **b)** Cross-link rates for the example RBPs CELF1 and HNRNPD after integration (n=3). Both cross-link at a high rate. **c)** Cross-link rates (all RNA) for the indicated RBPs with or without their recurrent missense mutations (n=3 except n=2 for DDX50). **d)** As panel C, but for minimal region RNA (n=3 except n=2 for DDX50). Bars, mean values.

Supplementary figure 18

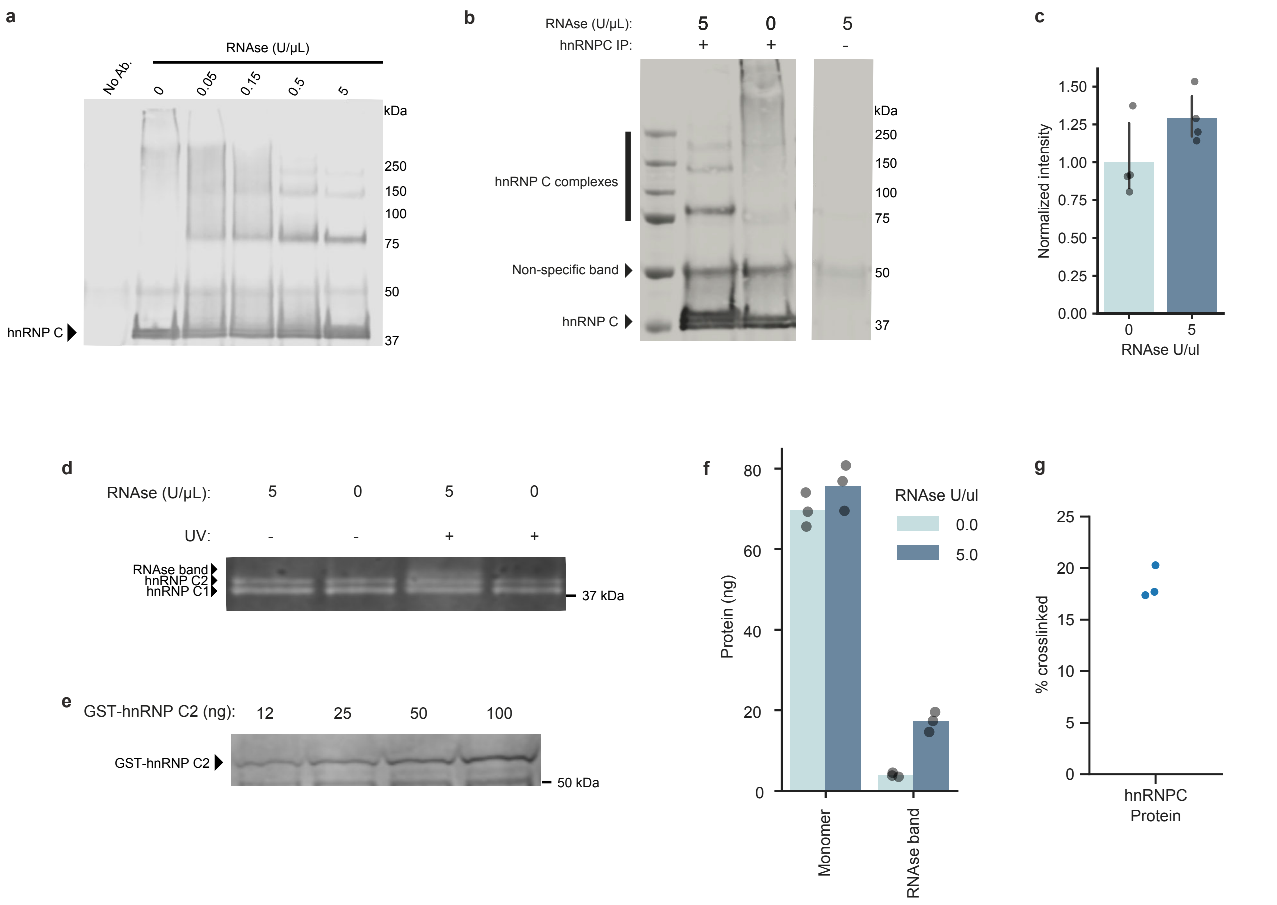

Supplementary figure 18. Quantification of cross-link rates for endogenous hnRNP C by immunoblot shift. Cells were UV cross-linked cells then hnRNP C was immunopurified. The change in western blot signal corresponding to monomeric hnRNP C was compared between RNAse concentrations (panels A-C). Because this change in signal is specifically for what can be collapsed with RNAse to monomeric hnRNP C, not for the un-collapsible higher molecular weight complexes spread throughout the lane, it should agree with the cross-linking number derived from dividing the RNA quantified in the minimal region by the monomeric hnRNP C signal (**Figure 4C**) and be lower than that derived from all RNA across the gel. Western blot quantification is complicated by the fact that absolute quantification requires protein in single bands of at least 5 ng, the narrow region of linear signal in immunoblots, and the fact that protein cross-linked to an over-digested 1-3 base fragment of RNA (~0.3-1 kDa) will run so close to un-cross-linked protein that it would not be distinct for a ~70 kDa protein[2]. **a)** RNAse digestion series of immunopurified hnRNP C (immunoblot, anti-hnRNP C). Experiment performed twice. **b)** Example replicate of +/- RNAse gels used to quantify the amount of shifted hnRNP C. Experiment performed twice. **c)** Quantification of the amount of shifted immunoblot signal comparing +/- RNAse gel lanes, as in panel B. The change in western blot signal was ~20%, close to the 22% cross-link number from Figure 4C. A more exact comparison was then performed, deriving the amount of hnRNP C protein dependent on both UV cross-linking and RNAse-digestion by absolute quantification of a western blot (panels D-F). Data is mean ± 95% CI for n=4 samples from two experiments. **d)** Gel used for absolute quantification of UV- and RNAse- depending monomeric hnRNP C signal. Experiment performed once with 3 replicates. **e)** Standards used for absolute quantification of gel data as in panel D. **f)** Quantification of the absolute amount of protein present in the bands in replicates like that in panel D. Bars represent the mean (n=3). **g)** The amount of hnRNP C cross-linked to RNA that is collapsible into the monomeric hnRNP C band, as determined by the absolute quantification data in panel F (n=3). This method also gave a cross-link rate of ~20%, again similar to the 22% observed in Figure 4C. It was concluded that this method of determining cross-link rates using absolute quantification of RNA and protein (Figures 2 and 3) was reasonably accurate. This verification was only possible for hnRNP C because of its very high cross-link rate and small size.

Supplementary figure 19

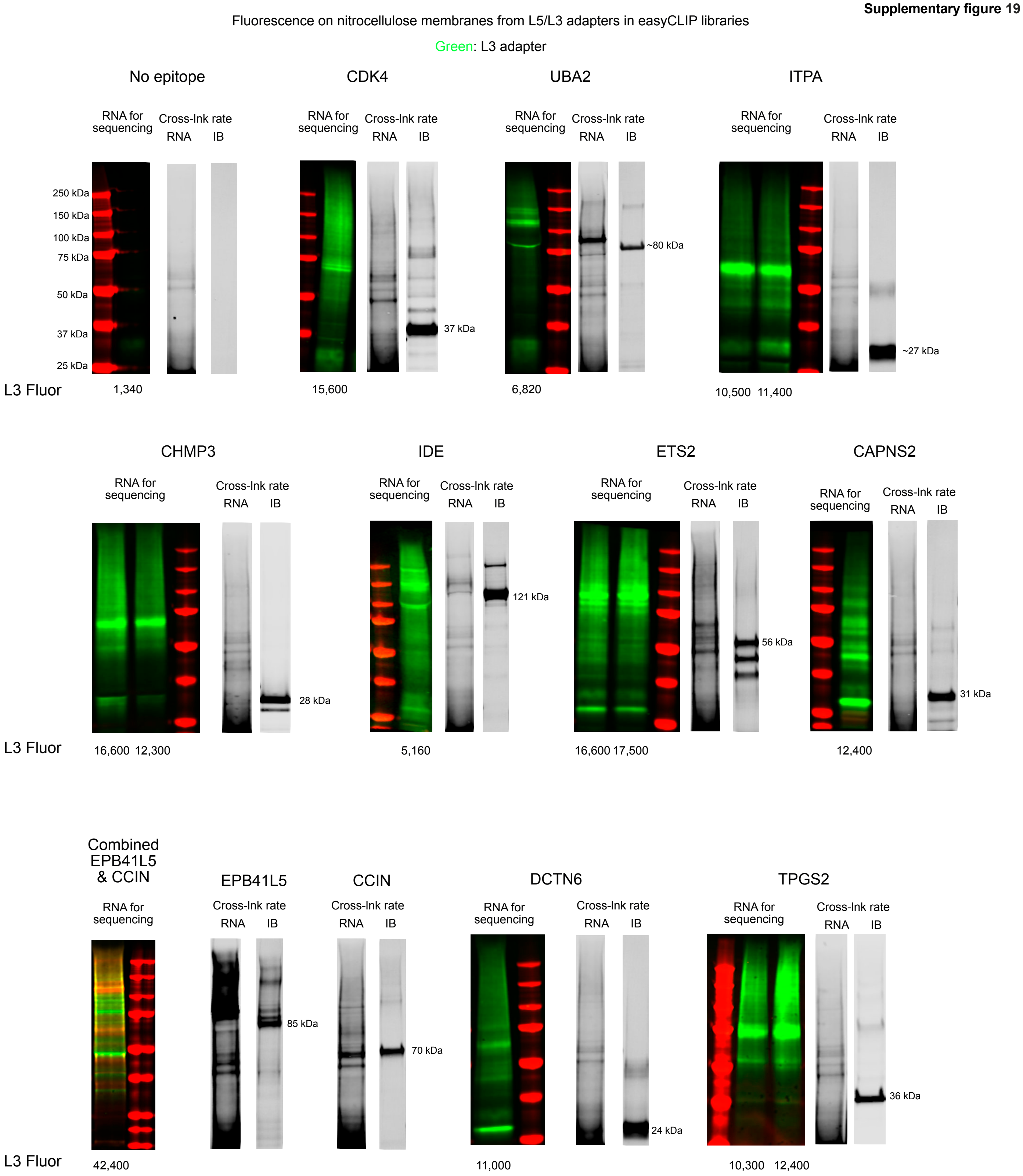

Supplementary figure 19. Images representing the purification of randomly selected HA-tagged non-RBPs, determination of cross-link rates and preparation of easyCLIP libraries for sequencing. Red represents L5 adapter fluorescence (or protein ladder), and green L3 adapter fluorescence. For RNA cross-link rate determination, experiment was performed 3 times (UBA2, ETS2, EPB41L5, CCIN) or 2 times (the others); for library preparation, experiment was performed four times (no epitope), three times (CDK4), twice (UBA2, ITPA, CHMP3, ETS2, CAPNS2, TPGS2), or once (IDE, EPB41L5/CCIN, DCTN6).

Supplementary figure 20

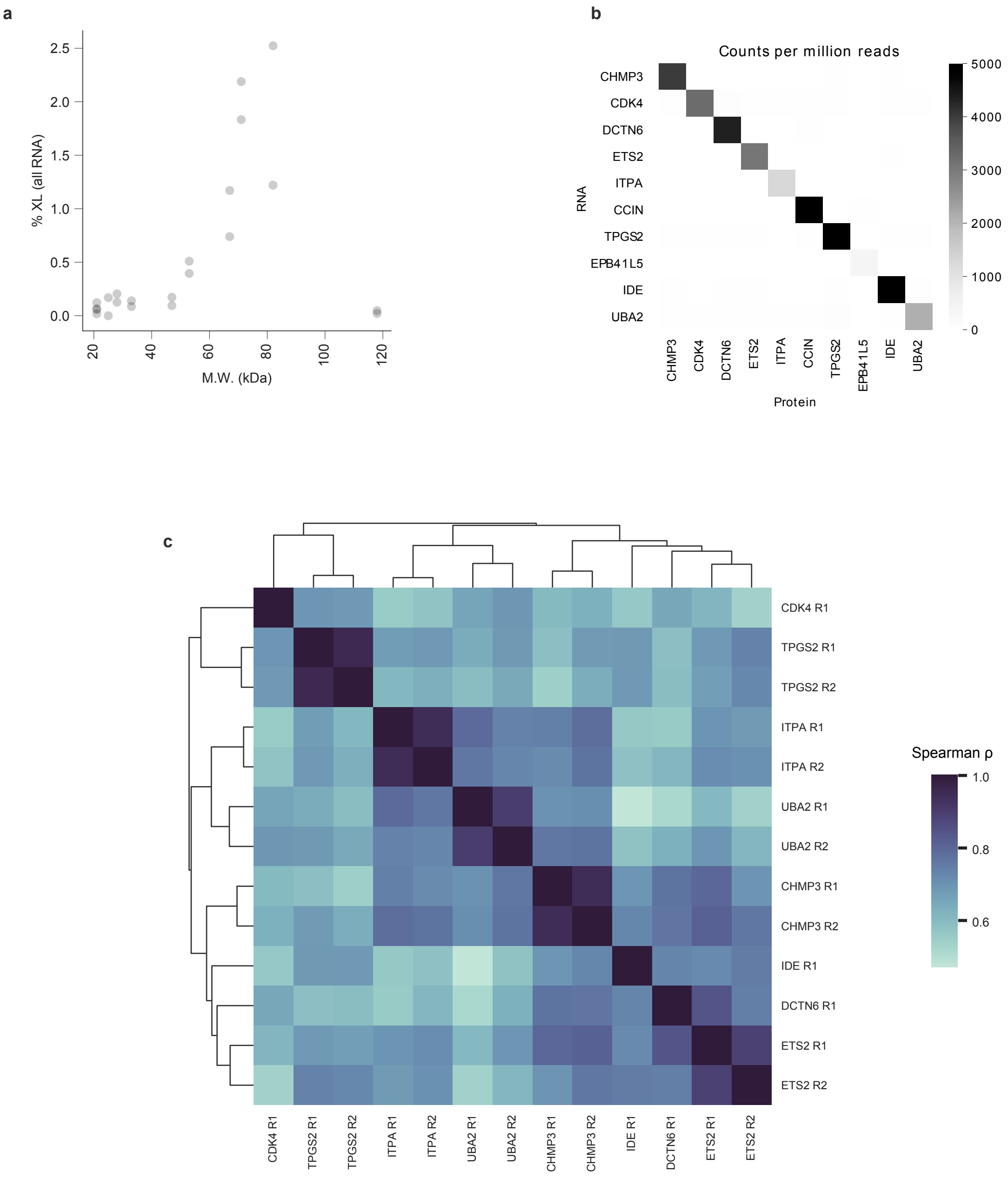

Supplementary figure 20. a) Total purified cross-linked RNA positively correlates with protein size for randomly selected non-RBPs. **b)** Read counts (per million reads) of the non-RBPs *vs* their own RNAs shows each non-RBP enriches for its respective RNA, a consequence of each non-RBP being expressed from a plasmid. This shows each library was generated from cells over-expressing the respective protein-of-interest, despite the fact that barcodes for multiple over-expression experiments were combined after each ligation. It also shows that if you express an RNA highly, it will show up in CLIP data, regardless of the purified protein. Counts were capped at 5,000 reads-per-million for visualization. Libraries for CAPNS6 were extremely small and were not included. **c)** Spearman correlations of easyCLIP binding in reads-per-gene (counting exons and introns separately) for non-RBPs. Four extremely small datasets (CAPNS6, CCIN, UBA2 Rep. 3, ETS2 Rep. 3) were not included. Clustering was based on the 498 RNAs with the most reads summed across all datasets.

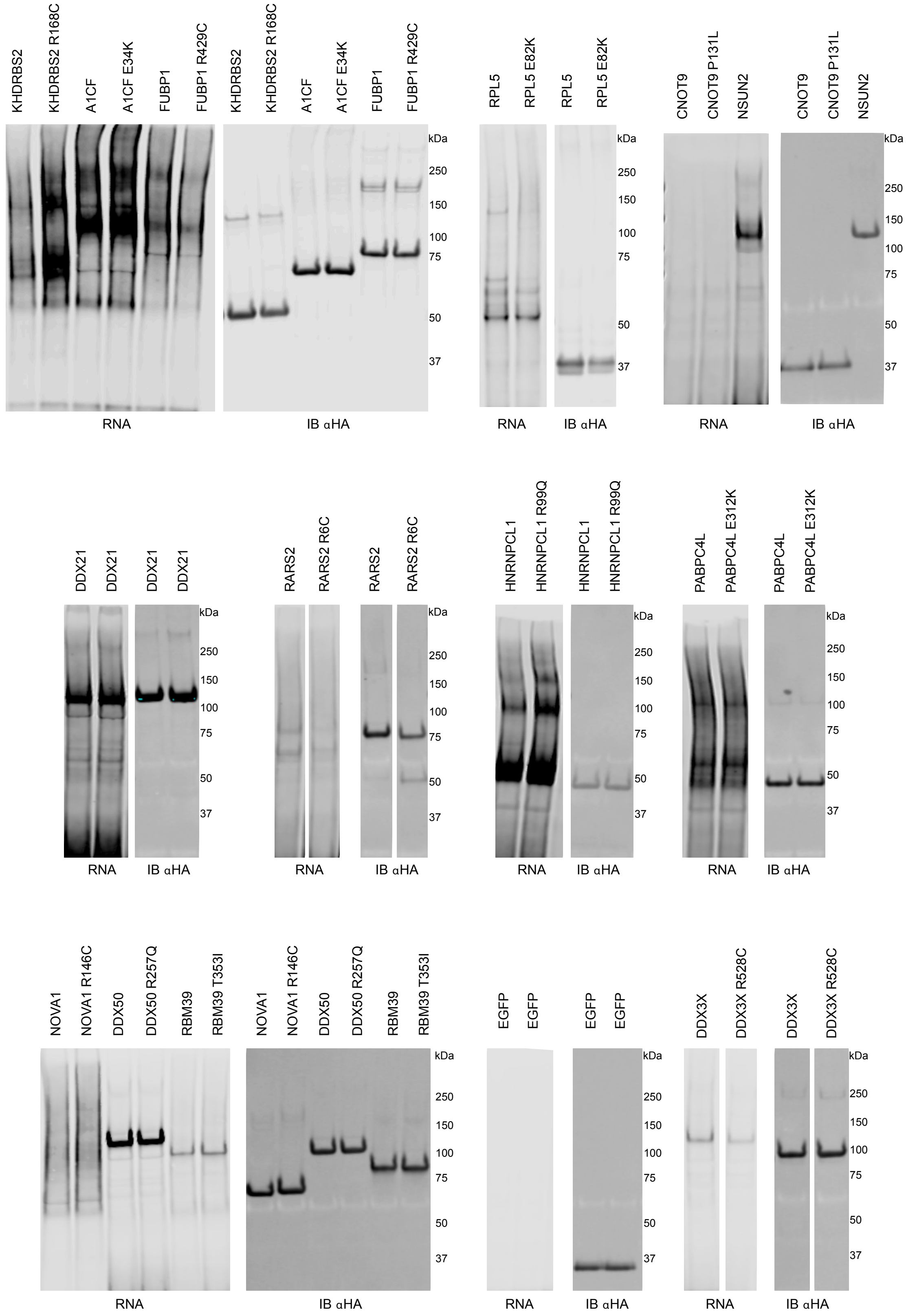

Supplementary figure 21. RNA fluorescence (L5 adapter) and immunoblot signal (anti-HA) for nitrocellulose membranes used to calculate cross-link efficiencies for the indicated proteins. Experiments were performed four times (KHDRBS2, A1CF, DDX50), three times (FUBP1), twice (RPL5, CNOT9, DDX21, RARS2, HNRNPCL1, PABPCL1, NOVA1, RBM39, EGFP, DDX3X), or once (NSUN2).

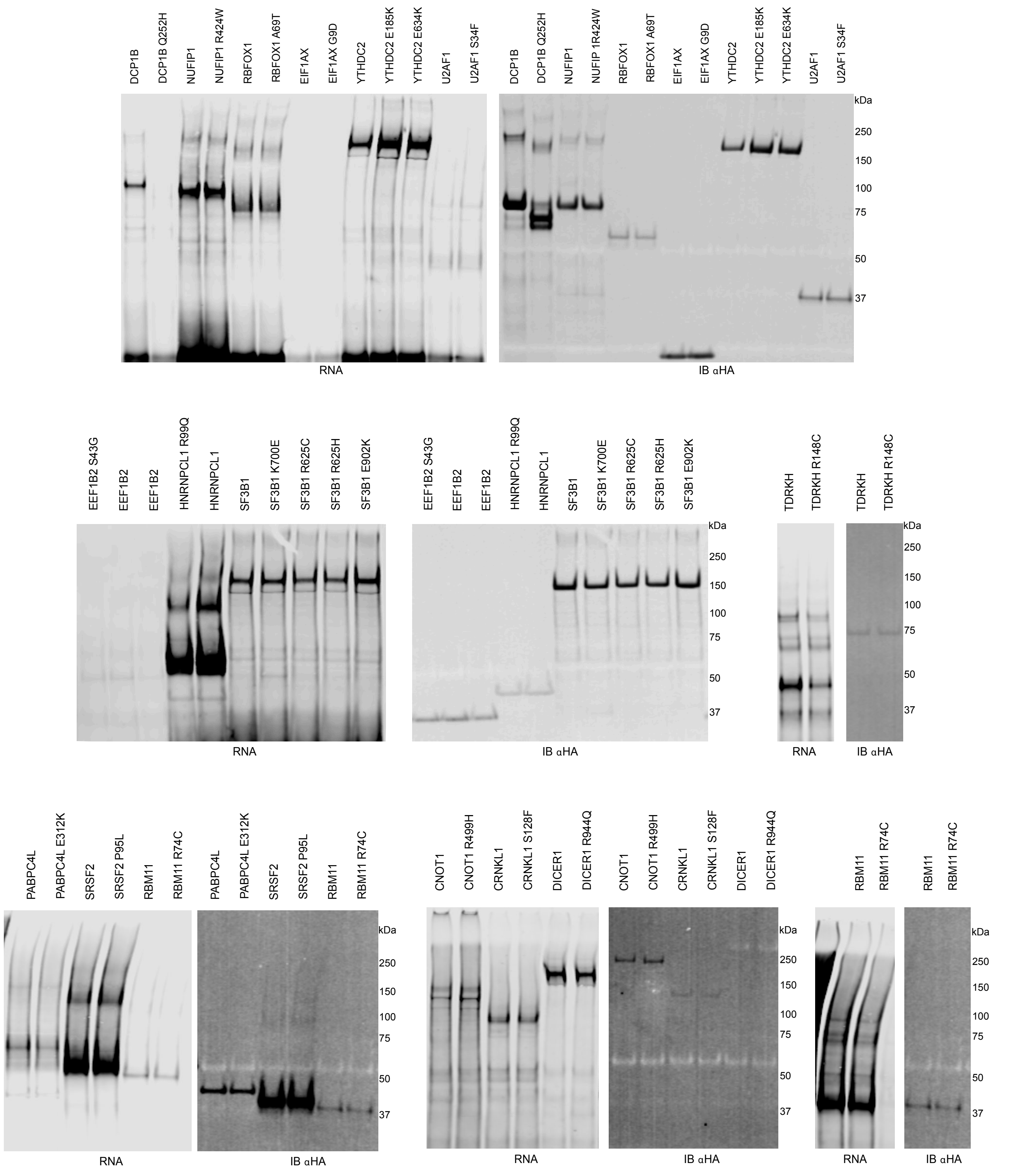

Supplementary figure 22. RNA fluorescence (L5 adapter) and immunoblot signal (anti-HA) for nitrocellulose membranes used to calculate cross-link efficiencies for the indicated proteins. Experiments were performed three times (EEF1B2), once (DICER1) or twice (the others).

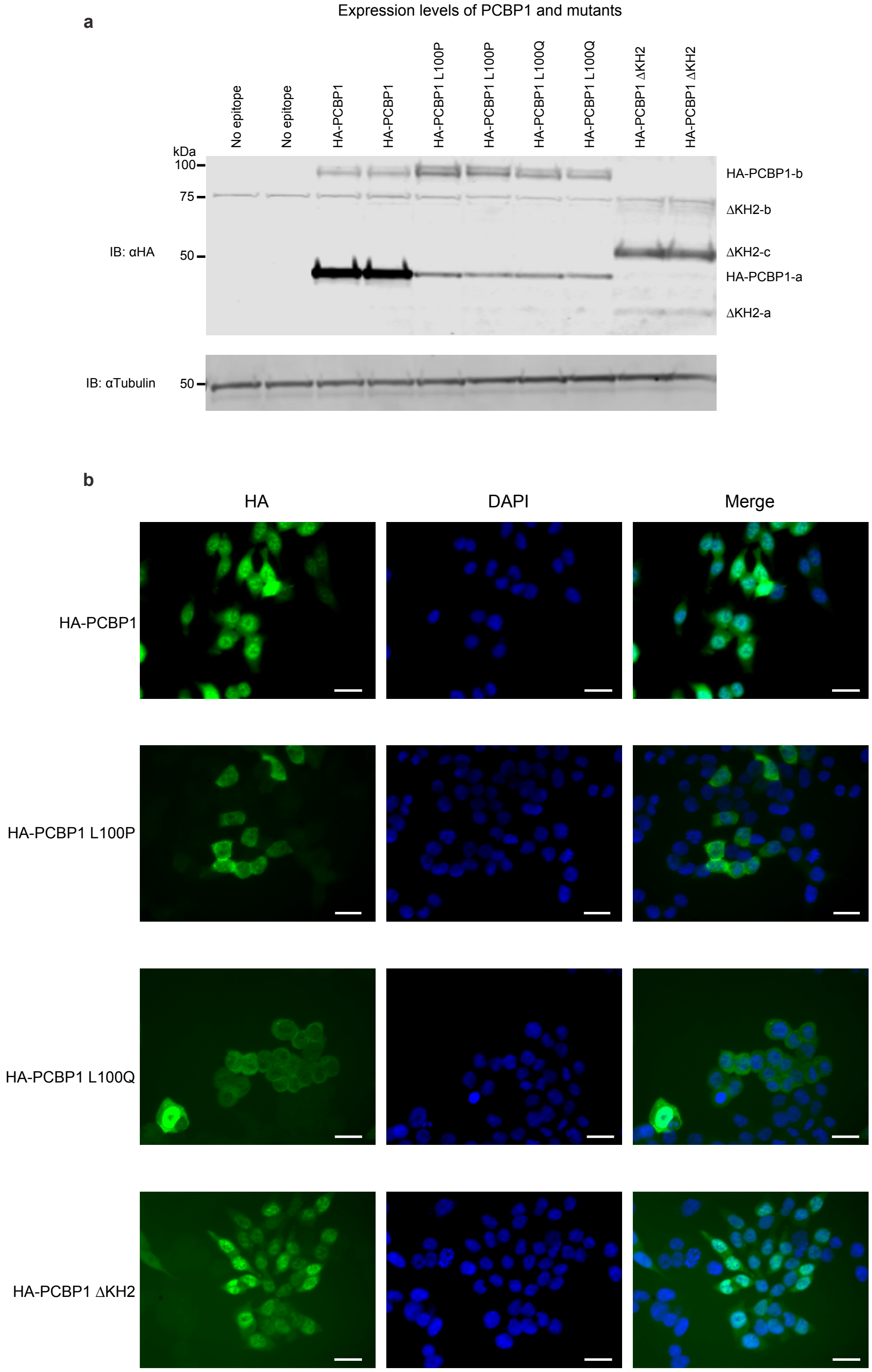

Supplementary figure 23. a) Expression levels of FH-PCBP1 and mutants in HCT116 cell lysate after UV cross-linking. The nature of the additional, higher molecular weight bands (b, c) is unknown. Experiment was performed 3 times. **b)** Microscopy of wild-type and mutant FHH-PCBP1 in HCT116 cells showing that L100P/Q mutants are less nuclear than wild-type or ΔKH2 PCBP1. All images were taken with the same settings (exposure time, *ect.*), on the same slide and day. Scale bar: 20 μm (approximate). Experiment was performed twice.

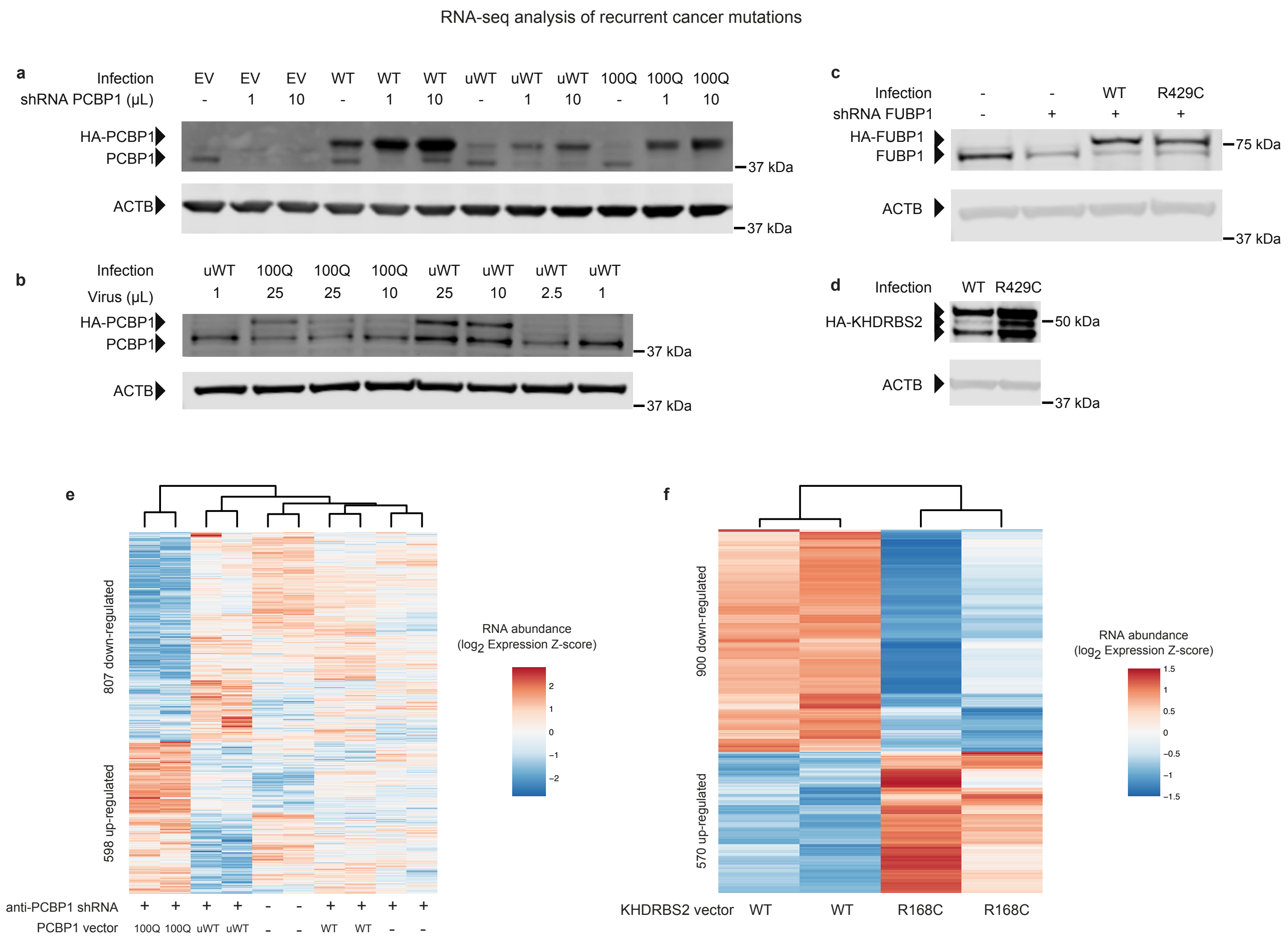

Supplementary figure 24. The effect of recurrent cancer mutations on the transcriptome as measured by RNA-seq. **a)** Immunoblot against PCBP1 in cells harvested for RNA-seq. Because shRNA against PCBP1 is toxic - PCBP1 is an essential gene - two levels of shRNA virus were tried. The sequenced libraries were had 1 µL virus. Note that more shRNA virus led to higher expression of integrated HA-PCBP1, suggesting a post-transcriptional feedback mechanism. Immunoblot experiments (panels A-D) were performed once. **b)** Immunoblot against PCBP1 in cells harvested for RNA-seq. These cells, harvested as a second control against protein abundance, are included in GSE162366, but there was insufficient space in this paper to analyze them. **c)** Immunoblot against FUBP1 in cells harvested for RNA-seq. RNA-seq libraries from these cells are included in GSE162366, but there was insufficient space in this paper to analyze them. **d)** Immunoblot against KHDRBS2 in cells harvested for RNA-seq. **e)** Transcriptomic changes from expressing wild-type or R168C KHDRBS2 in A375 cells *via* lentiviral integration. RNA abundance as log2 fold changes for RNAs with at least a 1.41-fold difference (e.g., $log_2(0.5)$) between wild-type and mutant samples are shown as a clustered heatmap. Each column is a replicate and there are two replicates per condition. **f)** Transcriptomic effects from knocking down endogenous PCBP1 and expressing wild-type PCBP1 (“WT”) or L100Q PCBP1 (“100Q”), wild-type PCBP1 with a uORF to lower expression (“uWT”), or empty vector (“-”) *via* viral integration in HCT116 cells and Puromycin selection. The heatmap depicts RNAs with at least a 1.41-fold difference in abundance between integrated uORF-WT and L100Q PCBP1 cells, both in cells treated with anti-PCBP1 shRNA to knock-down endogenous PCBP1. Samples were treated with either shRNA against the 3’UTR of PCBP1 (“+”) or non-targeting shRNA (“-”). Each column is a replicate and there are two replicates per condition.

Supplementary figure 25

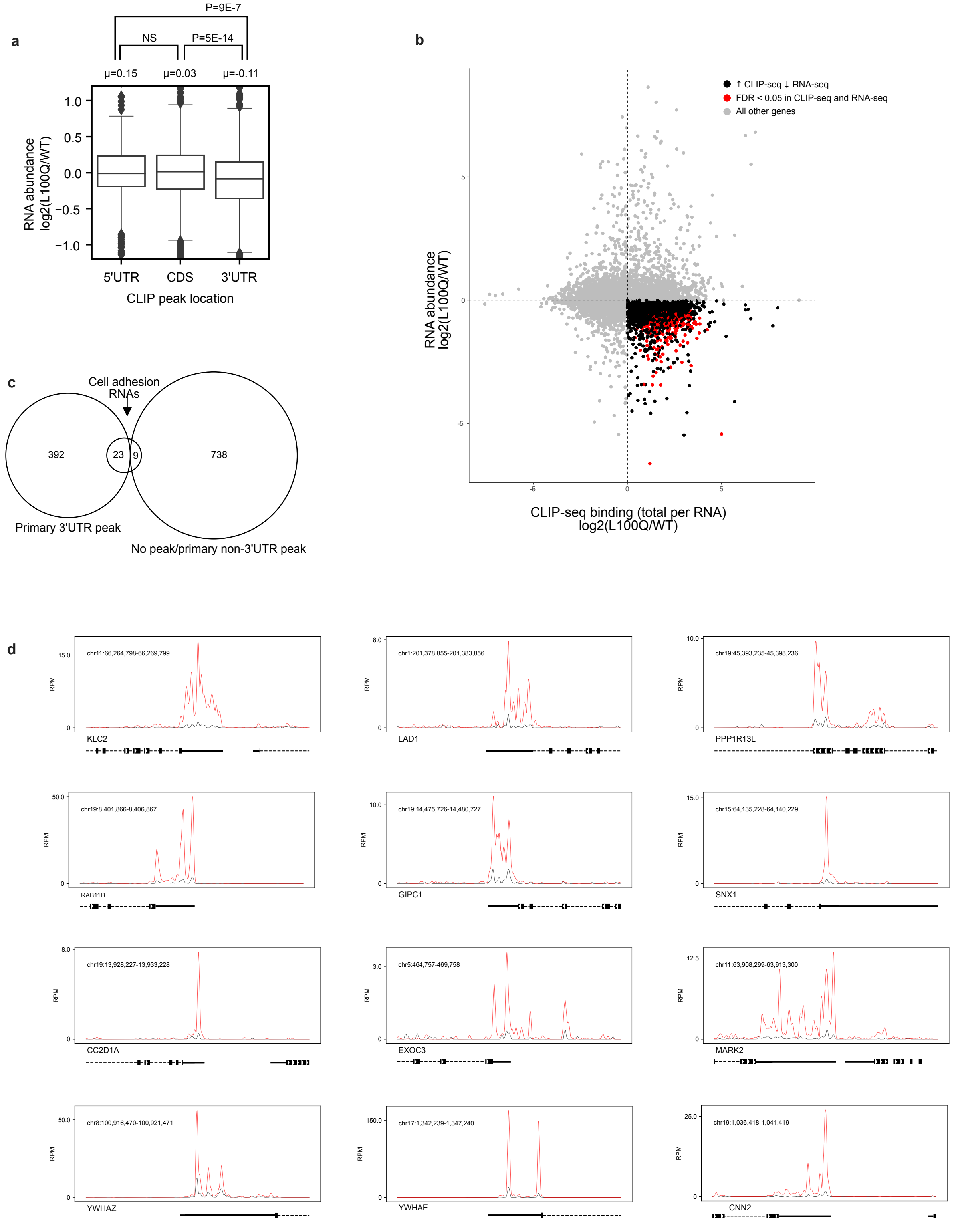

Supplementary figure 25. The effect of recurrent cancer mutations on the transcriptome as measured by RNA-seq. **a)** RNAs targeted by PCBP1 in the 3'UTR are more likely to be destabilized by L100Q PCBP1. RNA-abundance changes are between uORF-WT and L100Q PCBP1 HCT116 cells. 5'UTR, n=479; CDS, n=1858; 3'UTR, n=2013. Two-sided t-test. Boxplots show quartiles, center line shows the median and whiskers show maximum and minimum, except for those points beyond 1.5 * interquartile range, which are plotted individually. NS: not significant. **b)** Scatterplot of the relation between differential binding in CLIP-seq (*via* EdgeR) and RNA abundance (*via* Deseq2) for wild-type and L100Q PCBP1. CLIP binding was determined in HCT116 cells with WT and L100Q PCBP1 integrated into the genome. Red dots represent RNAs with FDR <0.05 for both a decrease in abundance and an increase in binding by L100Q PCBP1. **c)** Among RNAs with FDR <0.05 for both a decrease in abundance and an increase in binding by L100Q PCBP1, a majority had their primary peak location (by maximum signal density) placed outside the 3'UTR, but 23/32 with the cell-cell adhesion GO term had a primary peak in the 3'UTR (P<4E-5 for enrichment by Fisher's exact test). This rises to 23/27 of those with a peak assigned at all (in some cases, if signal was diffuse enough, the RNA was not assigned a peak). Visual inspection showed nearly all 32 had a 3'UTR peak of some kind. **d)** Examples of cell-cell adhesion genes with PCBP1 CLIP-seq reads-per-million plotted. L100Q binding is in red, WT PCBP1 in black. Introns are dashed lines, CDS thick lines, and UTRs thin lines. The top three rows are the RNAs with the largest fold decrease, in descending order of effect size. The bottom row are three additional examples.

References

1. Dominguez, D. *et al.* Sequence, Structure, and Context Preferences of Human RNA Binding Proteins. *Mol. Cell* **70**, 854-867.e9 (2018).
2. Dobin, A. *et al.* STAR: ultrafast universal RNA-seq aligner. *Bioinformatics* **29**, 15–21 (2013).
3. Li, B. & Dewey, C. N. RSEM: accurate transcript quantification from RNA-Seq data with or without a reference genome. *BMC Bioinformatics* **12**, 323 (2011).
4. Love, M. I., Huber, W. & Anders, S. Moderated estimation of fold change and dispersion for RNA-seq data with DESeq2. *Genome Biol.* **15**, 550 (2014).
5. Reyes, A. *et al.* Drift and conservation of differential exon usage across tissues in primate species. *Proc. Natl. Acad. Sci.* **110**, 15377 LP – 15382 (2013).
6. Anders, S., Reyes, A. & Huber, W. Detecting differential usage of exons from RNA-seq data. *Genome Res.* **22**, 2008–2017 (2012).
7. Huang, D. W., Sherman, B. T. & Lempicki, R. A. Systematic and integrative analysis of large gene lists using DAVID bioinformatics resources. *Nat. Protoc.* **4**, 44–57 (2009).
8. Harper, S. & Speicher, D. W. Purification of proteins fused to glutathione S-transferase. *Methods Mol. Biol.* **681**, 259–280 (2011).
9. Janes, K. A. An analysis of critical factors for quantitative immunoblotting. *Sci. Signal.* **8**, rs2 LP-rs2 (2015).
10. Luo, S., Wehr, N. B. & Levine, R. L. Quantitation of protein on gels and blots by infrared fluorescence of Coomassie blue and Fast Green. *Anal. Biochem.* **350**, 233–238 (2006).
11. Criscione, S. W., Zhang, Y., Thompson, W., Sedivy, J. M. & Neretti, N. Transcriptional landscape of repetitive elements in normal and cancer human cells. *BMC Genomics* **15**, 583 (2014).
