# Supplementary material for: easyCLIP analysis of RNA-protein interactions incorporating absolute quantification
Source: Nat Commun. 2021 Mar 10;12:1569. doi: 10.1038/s41467-021-21623-4 (PMC7946914; doi:10.1038/s41467-021-21623-4)
Supplement: Supplementary file 2 — Reporting Summary [file 41467_2021_21623_MOESM2_ESM.pdf]

nature research

Corresponding author(s): Paul A. Khavari

Last updated by author(s): Jan 11, 2021

Reporting Summary

Nature Research wishes to improve the reproducibility of the work that we publish. This form provides structure for consistency and transparency in reporting. For further information on Nature Research policies, see our Editorial Policies and the Editorial Policy Checklist.

Statistics

For all statistical analyses, confirm that the following items are present in the figure legend, table legend, main text, or Methods section.

n/a	Confirmed	
x		The exact sample size (*n*) for each experimental group/condition, given as a discrete number and unit of measurement
x		A statement on whether measurements were taken from distinct samples or whether the same sample was measured repeatedly
	x	The statistical test(s) used AND whether they are one- or two-sided<br>*Only common tests should be described solely by name; describe more complex techniques in the Methods section.*
x		A description of all covariates tested
	x	A description of any assumptions or corrections, such as tests of normality and adjustment for multiple comparisons
	x	A full description of the statistical parameters including central tendency (e.g. means) or other basic estimates (e.g. regression coefficient) AND variation (e.g. standard deviation) or associated estimates of uncertainty (e.g. confidence intervals)
	x	For null hypothesis testing, the test statistic (e.g. *F*, *t*, *r*) with confidence intervals, effect sizes, degrees of freedom and *P* value noted<br>*Give P values as exact values whenever suitable.*
x		For Bayesian analysis, information on the choice of priors and Markov chain Monte Carlo settings
x		For hierarchical and complex designs, identification of the appropriate level for tests and full reporting of outcomes
x		Estimates of effect sizes (e.g. Cohen's *d*, Pearson's *r*), indicating how they were calculated

Our web collection on statistics for biologists contains articles on many of the points above.

Software and code

Policy information about availability of computer code

Data collection	High-throughput sequencing was collected on Illumina HiSeq 2500, HiSeq 4000, and Miseq machines, all paired-end. The sequencing machine and read length for each dataset are in File S2.
Data analysis	Code used for analysis was placed at github.com/dfporter/easyCLIP. Code made use of cutadapt v. 2.9, STAR v. 2.7.4, bowtie2 v. 2.4.1, samtools v. 1.1, bedtools v. 2.27.1, Python v. 3.8, R v. 4.0.0, edgeR v. 3.30.0, Rsubread v. 1.32.4, DESeq2 v 1.24.0, DEXSeq v 1.30.0, pheatmap v. 1.0.12, RSEM v. 1.3.0, DAVID v. 6.8, and homer v. 4.11.

For manuscripts utilizing custom algorithms or software that are central to the research but not yet described in published literature, software must be made available to editors and reviewers. We strongly encourage code deposition in a community repository (e.g. GitHub). See the Nature Research guidelines for submitting code & software for further information.

Data

Policy information about availability of data

All manuscripts must include a data availability statement. This statement should provide the following information, where applicable:

- Accession codes, unique identifiers, or web links for publicly available datasets
- A list of figures that have associated raw data
- A description of any restrictions on data availability

HITS datasets have been deposited at Gene Expression Omnibus (GEO) under accessions GSE154168, GSE162366 and GSE131210.

GRCh38 genome Gencode release 29 and features were obtained from:
ftp://ftp.ebi.ac.uk/pub/databases/gencode/Gencode_human/release_29/GRCh38.primary_assembly.genome.fa.gz
ftp://ftp.ebi.ac.uk/pub/databases/gencode/Gencode_human/gencode.v29.primary_assembly.annotation.gtf.gz.

Field-specific reporting

Please select the one below that is the best fit for your research. If you are not sure, read the appropriate sections before making your selection.

- [x] Life sciences
- [ ] Behavioural & social sciences
- [ ] Ecological, evolutionary & environmental sciences

For a reference copy of the document with all sections, see nature.com/documents/nr-reporting-summary-flat.pdf

Life sciences study design

All studies must disclose on these points even when the disclosure is negative.

Sample size	Sample sizes for CLIP and immunoblots were determined by financial and time limitations.
Data exclusions	No data was excluded. A few CLIP replicates failed due to contamination or for unknown reasons, and were not included in the GEO repository or analysis.
Replication	Replicate numbers varied by experiment and sample. At least two independent replicates were used in all cases except some supplementary immunoblots and CLIP datasets (some of the non-RBP negative control datasets), where financial and time limitations precluded a second replicate.
Randomization	No randomization was required because millions of isogenic cells were used.
Blinding	No blinding was required because experiments were immunoblots, technical in vitro experiments with oligonucleotides or CLIP/ChIP experiments from common cell lines.

Reporting for specific materials, systems and methods

We require information from authors about some types of materials, experimental systems and methods used in many studies. Here, indicate whether each material, system or method listed is relevant to your study. If you are not sure if a list item applies to your research, read the appropriate section before selecting a response.

Materials & experimental systems

n/a	Involved in the study
[ ]	[x] Antibodies
[ ]	[x] Eukaryotic cell lines
[x]	[ ] Palaeontology and archaeology
[x]	[ ] Animals and other organisms
[x]	[ ] Human research participants
[x]	[ ] Clinical data
[x]	[ ] Dual use research of concern

Methods

n/a	Involved in the study
[x]	[ ] ChIP-seq
[x]	[ ] Flow cytometry
[x]	[ ] MRI-based neuroimaging

Antibodies

Antibodies used

Mouse monoclonal HA Tag antibody (16B12), conjugated to Alexa Fluor 488 (ThermoFisher #A21287)
Mouse monoclonal anti-hnRNP C antibody (4F4), Alexa 790-conjugated (Santa Cruz Biotechnology sc-32308 AF790)
Mouse monoclonal Anti-hnRNP C antibody (4F4) (Santa Cruz Biotechnology sc-32308)
Mouse monoclonal anti-HA tag antibody (6E2) (Cell Signalling, #2367)
Rabbit polyclonal anti-HA tag antibody (Abcam, ab137838)
Mouse monoclonal anti-Actin antibody (Sigma, A2228)
Rabbit polyclonal anti-Fibrillarin antibody (Bethyl A303-891A)
Rabbit polyclonal anti-RBFOX2 antibody (Bethyl, A300-864A)
Rabbit polyclonal anti-RBFOX1 antibody (Abclonal A7811)
Rabbit polyclonal anti-A1CF antibody (Aviva systems biology OACD01340)
Rabbit polyclonal anti-KHDRBS2 antibody (Sigma HPA037779)
Rabbit polyclonal anti-RBFOX1 antibody (Proteintech 22647-1-AP)
Rabbit polyclonal anti-RBM11 antibody (Proteintech 17220-1-AP)
Rabbit polyclonal anti-DDX50 antibody (Proteintech 10358-1-AP)
Rabbit polyclonal anti-RBM39 antibody (Proteintech 21339-1-AP)
Rabbit polyclonal anti-HNRNPD antibody (Proteintech 12770-1-AP)
Rabbit polyclonal anti-FUBP1 antibody (Proteintech 24864-1-AP)
Rabbit polyclonal anti-EEF1B2 antibody (Proteintech 10095-2-AP)

	Rabbit polyclonal anti-RPL5 antibody (Proteintech 15430-1-AP)<br>Rabbit polyclonal anti-EIF4H antibody (Proteintech 11012-1-AP)<br>Rabbit polyclonal anti-CELF1 antibody (Proteintech 13002-1-AP)<br>Mouse monoclonal anti-DDX3X antibody (Santa Cruz Biotechnology, sc-81247)<br>Rabbit polyclonal anti-PCBP1 antibody (MBL, RN024P)<br>Rabbit polyclonal anti-NUFP1 antibody (Proteintech 12515-1-AP)<br>Rabbit polyclonal anti-U2AF1 antibody (Proteintech 10334-1-AP)<br>Goat polyclonal IRDye 800CW anti-Rabbit IgG Secondary Antibody (LI-COR P/N: 926-32211)<br>Goat polyclonal IRDye 680RD anti-Rabbit IgG Secondary Antibody (LI-COR P/N: 926-68071)
Validation	IB: immunoblot. WCL: whole cell lysate. endog.: endognous<br><br>HA Tag antibody (16B12), conjugated to Alexa Fluor 488 (ThermoFisher #A21287): IB signal at expected size observed in WCL for recombinant protein.<br>anti-hnRNP C (4F4), Alexa 790-conjugated (sc-32308 AF790): IB signal at expected size observed in WCL for endog. protein.<br>anti-hnRNP C (4F4) (sc-32308): IB signal at expected size observed in WCL for endog. protein.<br>anti-HA tag (6E2) (Cell Signalling, #2367): IB signal at expected size observed in WCL for recombinant protein.<br>anti-HA tag (Abcam, ab137838): IB signal at expected size observed in WCL for recombinant protein.<br>anti-Actin (Sigma, A2228): IB signal at expected size observed in WCL for endog. protein.<br>anti-Fibrillarin (Bethyl A303-891A): IB signal at expected size observed in WCL for endog. protein.<br>anti-RBFOX2 (Bethyl, A300-864A): IB signal at expected size observed in WCL for endog. protein.<br>anti-RBFOX1 (Abclonal A7811): IB signal at expected size observed in WCL for recombinant protein.<br>anti-A1CF (Aviva OACD01340): IB signal at expected size observed in WCL for endog. and recombinant protein.<br>anti-KHDRBS2 (Sigma HPA037779): IB signal at expected size observed in WCL for endog. and recombinant protein.<br>anti-RBFOX1 (Proteintech 22647-1): IB signal at expected size observed in WCL for recombinant protein.<br>anti-RBM11 (Proteintech 17220-1): IB signal at expected size observed in WCL for endog. and recombinant protein.<br>anti-DDX50 (Proteintech 10358-1): IB signal at expected size observed in WCL for endog. and recombinant protein.<br>anti-RBM39 (Proteintech 21339-1): IB signal at expected size observed in WCL for endog. and recombinant protein.<br>anti-HNRNPD (Proteintech 12770-1): IB signal at expected size observed in WCL for endog. and recombinant protein.<br>anti-FUBP1 (Proteintech 24864-1): IB signal at expected size observed in WCL for endog. and recombinant protein.<br>anti-EEF1B2 (Proteintech 10095-2): IB signal at expected size observed in WCL for endog. and recombinant protein.<br>anti-RPL5 (Proteintech 15430-1): IB signal at expected size observed in WCL for endog. and recombinant protein.<br>anti-EIF4H (Proteintech 11012-1): IB signal at expected size observed in WCL for endog. and recombinant protein.<br>anti-CELF1 (Proteintech 13002-1): IB signal at expected size observed in WCL for endog. and recombinant protein.<br>anti-DDX3X (Santa Cruz, sc-81247): IB signal at expected size observed in WCL for endog. and recombinant protein.<br>anti-PCBP1 (MBL, RN024P): IB signal at expected size observed in WCL for endog. and recombinant protein.<br>anti-NUFP1 (Proteintech 12515-1): IB signal at expected size observed in WCL for endog. and recombinant protein.<br>anti-U2AF1 (Proteintech 10334-1): IB signal at expected size observed in WCL for endog. and recombinant protein.

Eukaryotic cell lines

Policy information about cell lines

Cell line source(s)	HEK293T Lenti-X cells were obtained from Takara Bio. HCT116, A375 and HepG2 cells were obtained from ATCC.
Authentication	Morphology and behavior matched expectations; CLIP sequencing showed the expected inclusion/exclusion of the Y chromosome.
Mycoplasma contamination	All cell lines tested negative for mycoplasma.
Commonly misidentified lines<br>(See ICLAC register)	No commonly misidentified cell lines were employed.
