# Supplementary material for: easyCLIP analysis of RNA-protein interactions incorporating absolute quantification
Source: Nat Commun. 2021 Mar 10;12:1569. doi: 10.1038/s41467-021-21623-4 (PMC7946914; doi:10.1038/s41467-021-21623-4)
Supplement: Supplementary file 11 — Description of Additional Supplementary Files [file 41467_2021_21623_MOESM11_ESM.pdf]

Title: Supplementary Data 1
Description: The full easyCLIP protocol and oligonucleotide sequences.

Title: Supplementary Data 2
Description: Description of high-throughput sequencing datasets included in this study.

Title: Supplementary Data 3
Description: Raw counts, counts per million reads, and counts per ten billion proteins for all proteins.

Title: Supplementary Data 4
Description: P values for all proteins across all RNAs, determined by negative binomial fits to random non-RBPs in all cases.

Title: Supplementary Data 5
Description: Peak locations for all proteins in all RNAs.

Title: Supplementary Data 6
Description: Cross-linking molecule number estimates.

Title: Supplementary Data 7
Description: EdgeR analysis of easyCLIP data for wild-type and mutant forms of recurrently mutated RBPs. P values were calculated with the EdgeR function glmQLFTest.

Title: Supplementary Data 8
Description: RNA-seq analysis of recurrent missense mutations. Adjusted P values calculated by DESeq2 (see methods).
